# Genomic Diversification in Strains of *Rickettsia felis* Isolated from Different Arthropods

**DOI:** 10.1093/gbe/evu262

**Published:** 2014-12-04

**Authors:** Joseph J. Gillespie, Timothy P. Driscoll, Victoria I. Verhoeve, Tadanobu Utsuki, Claudia Husseneder, Vladimir N. Chouljenko, Abdu F. Azad, Kevin R. Macaluso

**Affiliations:** ^1^Department of Microbiology and Immunology, University of Maryland School of Medicine, Baltimore, Maryland; ^2^Department of Biology, Western Carolina University; ^3^Department of Pathobiological Sciences, Louisiana State University, School of Veterinary Medicine; ^4^Department of Entomology, Louisiana State University Agricultural Center, Baton Rouge, Louisiana

**Keywords:** flea, booklouse, genomes, plasmid, phylogeny, transitional group rickettsiae, Rickettsiales amplified genetic element, RTX-type I secretion system

## Abstract

*Rickettsia felis* (*Alphaproteobacteria*: Rickettsiales) is the causative agent of an emerging flea-borne rickettsiosis with worldwide occurrence. Originally described from the cat flea, *Ctenocephalides felis*, recent reports have identified *R*. *felis* from other flea species, as well as other insects and ticks. This diverse host range for *R*. *felis* may indicate an underlying genetic variability associated with host-specific strains. Accordingly, to determine a potential genetic basis for host specialization, we sequenced the genome of *R*. *felis* str. LSU-Lb, which is an obligate mutualist of the parthenogenic booklouse *Liposcelis bostrychophila* (Insecta: Psocoptera). We also sequenced the genome of *R*. *felis* str. LSU, the second genome sequence for cat flea-associated strains (cf. *R*. *felis* str. URRWXCal2), which are presumably facultative parasites of fleas. Phylogenomics analysis revealed *R*. *felis* str. LSU-Lb diverged from the flea-associated strains. Unexpectedly, *R*. *felis* str. LSU was found to be divergent from *R*. *felis* str. URRWXCal2, despite sharing similar hosts. Although all three *R*. *felis* genomes contain the pRF plasmid, *R*. *felis* str. LSU-Lb carries an additional unique plasmid, pLbaR (plasmid of *L*. *bostrychophila* associated *Rickettsia*), nearly half of which encodes a unique 23-gene integrative conjugative element. Remarkably, pLbaR also encodes a repeats-in-toxin-like type I secretion system and associated toxin, heretofore unknown from other Rickettsiales genomes, which likely originated from lateral gene transfer with another obligate intracellular parasite of arthropods, *Cardinium* (Bacteroidetes). Collectively, our study reveals unexpected genomic diversity across three *R*. *felis* strains and identifies several diversifying factors that differentiate facultative parasites of fleas from obligate mutualists of booklice.

## Introduction

Included in the *Alphaproteobacteria*, all known species of Rickettsiales (within the families Holosporaceae, Rickettsiaceae, Anaplasmataceae, and Midichloriaceae) are Gram-negative obligate intracellular bacteria associated with a wide range of eukaryotes (Gillespie, Nordberg, et al. 2012). The genus *Rickettsia* (Rickettsiaceae) is an extraordinarily diverse assemblage of species known from plants, amoebae, ciliates, arthropods, annelids, leeches, and vertebrates (Driscoll et al. 2013; Kang 2014). Although early-branching lineages of *Rickettsia* remain poorly characterized (Perlman et al. 2006), the derived lineage contains over 20 described species, many of which are pathogenic to their eukaryotic host and/or vector (Sahni et al. 2013). These described species, which are all associated with arthropods (e.g., ticks, mites, fleas, and body lice), and most with vertebrates (e.g., humans, cats and rodents), are currently organized into four groups: Ancestral Group (AG), Typhus Group (TG), Transitional Group (TRG), and Spotted Fever Group (SFG) (Gillespie et al. 2008). Collectively, this clade of rickettsial species transmitted by hematophagous arthropods, which contains several agents of human disease (e.g., Epidemic typhus, Rocky Mountain spotted fever, murine typhus, Mediterranean spotted fever), provides an opportunity to identify rickettsial determinants of transmission and pathogenicity.

Among the more recently recognized emerging rickettsial agents is *Rickettsia felis*, a member of TRG rickettsiae (Gillespie et al. 2007) and the causative agent of a typhus-like flea-borne rickettsiosis (Azad et al. 1997). Since initially identified in the cat flea, *Ctenocephalides felis* (Adams et al. 1990), *R*. *felis* has been acknowledged as an increasingly important emerging pathogen (Richards et al. 2010; Socolovschi et al. 2010; Parola 2011; Maina et al. 2012). Although the genome sequence of *R*. *felis* str. URRWXCal2 revealed a coding repertoire distinct from other rickettsial genomes (Ogata, Renesto, et al. 2005), little is known regarding intraspecific genetic diversity. Several *R*. *felis* strains have been identified in a broad range of invertebrate hosts (reviewed in Reif and Macaluso [2009]), including a novel isolate of *R*. *felis* from the nonhematophagous, parthenogenic booklouse *Liposcelis bostrychophila* (Insecta: Psocoptera) (Yusuf and Turner 2004; Behar et al. 2010; Thepparit et al. 2011). In its booklouse host, *R*. *felis* is an obligate mutualist required for the early development of the oocyte and is maintained 100% transovarially (Yusuf and Turner 2004; Perotti et al. 2006). Clearance of the organism from larvae by increased temperature (Perotti et al. 2006) and adult insects through antibiotics (Yusuf and Turner 2004) results in decreased longevity and fecundity, as well as nonviable egg production. Conversely, no overt fitness effect on cat fleas infected with *R*. *felis* has been identified, and transovarial transmission is highly variable, likely due to differences in protocols for the maintenance of laboratory-infected fleas (Higgins et al. 1994; Reif and Macaluso 2009). Thus, *R*. *felis* strains infecting cat fleas can be considered facultative parasites (as opposed to “symbionts” because all *Rickettsia* species demand considerable metabolites from host cells). Accordingly, the potential exists that genetic diversity between *R*. *felis* isolates contributes to the observed variation in host fitness effects as well as the divergent lifecycles of these bacteria.

In an effort to determine whether genetic variability underlays *R*. *felis* host specialization, we sequenced the genomes of two strains: 1) *R*. *felis* (str. LSU-Lb), which is 100% present within an all-female *L. bostrychophila* colony (Thepparit et al. 2011), and 2) *R*. *felis* str. LSU, which has a variable infection rate within an established *C*. *felis* colony (Pornwiroon et al. 2006). These genomes were compared extensively with the complete genome of *R*. *felis* str. URRWXCal2, which was also isolated from *C*. *felis* (Ogata, Robert, et al. 2005). Surprisingly, genetic variation was found to underlie all three strains, revealing heretofore unknown diversity associated with not only host specialization (flea vs. booklouse) but also spatial isolation (str. URRWXCal2 vs. str. LSU). This genomic comparison of host-dependent *R*. *felis* strains, that is, hematophagous (cat flea) versus nonhematophagous (booklice) arthropod hosts, provides novel insight into the molecular basis for rickettsial virulence and horizontal transmission. Our work also underscores the potential of the mobilome for orchestrating *Rickettsia* host adaptations.

## Materials and Methods

### *R*. *felis* Cultivation, Bacterial Purification, and DNA Isolation

*Rickettsia felis* str. LSU (Pornwiroon et al. 2006) and *R*. *felis* str. LSU-Lb (Thepparit et al. 2011), which were isolated from LSU colonies of *C. felis* (cat fleas) and *L. bostrychophila* (booklice), respectively, were propagated in the tick-derived ISE6 cell line in modified L15B growth medium (Pornwiroon et al. 2006). Cultures (passage 4) were partially purified from ISE6 cells as previously described (Sunyakumthorn et al. 2008). Briefly, *R. felis*-infected ISE6 were needle lysed and passed through a 2-μM syringe filter. Rickettsiae were collected by centrifugation (21,000 × g) and rinsed with NaCl solution (0.85 w/v). Concentrated bacteria was resuspended in 200 μM of manufacturer’s PBS buffer and total DNA was extracted using the PureLink Genomic DNA mini Kit (Invitrogen) as described by the manufacturer’s instructions.

### Genome Sequencing

The Ion Torrent Next Generation Sequencing technology was utilized for sequencing the genomes of *R*. *felis* str. LSU and *R*. *felis* str. LSU-Lb. Ion Xpress Plus Fragment Library Kit (Life Technologies) was used to prepare high-quality fragment libraries from total gDNA for sequencing on the Ion Personal Genome Machine (PGM) System. This kit includes: Ion Shear Plus Reagents for enzymatic fragmentation of genomic DNA and Ion Plus Fragment Library Kit for library preparations from the enzymatically fragmented DNA (a total of 1 μg of DNA was used for library construction). Template-positive Ion Sphere Particles (ISPs) containing clonally amplified DNA were produced using the Ion OneTouch 200 Template Kit v2 (for 200 base-read libraries) with the Ion OneTouch Instrument. The Ion OneTouch ES instrument was used to enrich ISPs intended for the Ion PGM System using the Ion PGM 200 Sequencing Kit and an Ion 316 sequencing chip. A total sequence output of Q20 quality, derived from the predicted per-base quality scores and corresponding to an error rate of 1%, is approximately 600 Mb for the Ion 316 chip.

### Single Nucleotide Polymorphism Analysis

#### Single Nucleotide Polymorphism Estimation

For *R. felis* str. LSU and *R. felis* str. LSU-Lb genomes, single nucleotide polymorphisms (SNPs) were determined as follows. Raw sequence reads from *R. felis* str. LSU and *R. felis* str. LSU-Lb libraries were independently mapped to the *R*. *felis* str. URRWXCal2 genome (National Center for Biotechnology Information [NCBI] taxonomy ID 315456) using the long-read option of the Burrows–Wheeler Aligner (BWA) v0.5.9 and a minimum score threshold of 200. Variants were called with mpileup and bcftools, part of the samtools v0.1.19 package, using the following settings: A maximum allowed depth of 10,000 reads per position (-d); indels called only when the average per-sample depth does not exceed 1,000 (-L); a minimum base quality of 7 (-Q); a coefficient for modeling homopolymer errors of 50 (-h); a gap-opening sequencing error probability of 10 (-o); a gap extension error probability of 17 (-e); and a minimum of four gapped reads required to consider indel candidates (-m). Genomic context (intergenic, repeat, or coding region) and substitution impact (synonymous, nonsynonymous, or silent) were determined using the *R. felis* str. URRWXCal2 chromosome (GenBank ID CP000053) and plasmid pRF (CP000054) annotations.

#### SNP Validation

For gene variant confirmation, all 23 SNPs predicted from the *R*. *felis* str. LSU reads, as well as 15 selected SNPs predicted from the *R*. *felis* str. LSU-Lb reads, were selected for sequencing. Genomic and plasmid DNA was extracted using Qiagen DNEasy Tissue Kit (QIAGEN, Valencia, CA). Primers for each variant were designed from flanking regions on contigs using Primer3Plus (Untergasser et al. 2007) and synthesized by Integrated DNA Technologies. Primers are listed in supplementary table S1, Supplementary Material online. Gene variants were polymerase chain reaction (PCR)-amplified using FastStart PCR Master Mix (Roche) 50 ng of DNA template, and 300 μM of each primer under standard thermocycling conditions on a PTC-200 PCR machine (MJ Research, Waltham, MA). The amplified PCR products were gel purified using QIAquick Gel Extraction Kit (QIAGEN) and bidirectionally sequenced with corresponding primers on an ABI PRISM 3130 Automated sequencer (Applied Biosystems) at the GeneLab core facility in the School of Veterinary Medicine at LSU. In several cases where direct sequencing failed, purified PCR products were cloned into pCR2.1-TOPO (InvitrogenTM, Life Technologies) according to manufacturer’s instructions. Plasmid inserts were sequenced by the LSU Gene Lab using the M13 forward (-20) and M13 reverse (-24) primer pairs.

### Genome Assembly

Raw reads from each strain were subsequently assembled de novo to the level of contig using Mira v3.4.1.1 under default settings, except the number of passes was set to 4, “skim each pass” was enabled, and minimum read length was set to 50. Homology searches (vs. NCBI’s NR database) were used to partition the contigs into rickettsial and nonrickettsial sequences. Additionally, reads from each LSU strain were mapped to full-length 16S rDNA sequences in the GreenGenes database from May 2013 (v. 13_5) using the long-read option of BWA v0.5.9, and binned by taxonomy to assess the microbial diversity in each sample. Rickettsial sequences were subsequently ordered against *R. felis* str. URRWXCal2 (CP000053 and CP000054) using ABACAS 1.3.1, and overlapping contigs merged into supercontigs. Additional contigs from *R. felis* str. LSU that matched to *Wolbachia* spp. were similarly ordered against *Wolbachia* endosymbiont of *Cimex lectularius* (AP013028), a close relative identified by 16S rDNA sequence analysis (data not shown). The assemblies and related information for the *R*. *felis* str. LSU and *R*. *felis* str. LSU-Lb genomes were deposited in GenBank and assigned the Bioproject IDs PRJNA258182 and PRJNA258188, respectively.

### Gene Prediction and Annotation

Gene models were predicted on rickettsial supercontigs from each LSU strain using two approaches: 1) The ab initio gene prediction program fgenesb (Tyson et al. 2004), trained on coding sequence (CDS) from *R. felis* str. URRWXCal2, and 2) the Rapid Annotation using Subsystem Technology (RAST) v2.0 server (Aziz et al. 2008). The fgenesb-based gene models were used for all comparisons among *R*. *felis* strains, whereas the RAST-based gene models were used for analyses across all Rickettsiaceae genomes. RAST-based annotations were transferred to the fgenes gene models using BLASTP to identify common gene predictions across both approaches. Gene models were also predicted on *Wolbachia*-like supercontigs within the *R. felis* str. LSU assembly, using RAST as described above. The gene models and annotations for the *R*. *felis* str. LSU and *R*. *felis* str. LSU-Lb genomes were deposited in GenBank and assigned accession numbers JSEM00000000 and JSEL00000000, respectively. All data for the uncharacterized *Wolbachia* species will be published elsewhere.

### Comparative Genomic Analysis

#### Protein Family Clustering

Utilizing FastOrtho, an in-house modified version of OrthoMCL (Li et al. 2003), four separate sets of orthologous groups (OGs) of proteins were generated: 1) 62 Rickettsiaceae genomes (supplementary table S2, Supplementary Material online); 2) the chromosomes of *R*. *felis* strains URRWXCal2 (NC_007109), LSU, and LSU-Lb; 3) 20 *Rickettsia* plasmids (supplementary table S3, Supplementary Material online); and 4) the pRF plasmid sequences of *R*. *felis* strains URRWXCal2 (NC_007110), LSU, “AUS-Lb” (GQ329881), and LSU-Lb. AUS-Lb refers to an unnamed *R*. *felis* strain collected from *L*. *bostrychophila* in the Darling Downs region of Queensland, Australia (Behar et al. 2010). Generated OGs were used in various analyses, as further outlined below.

#### Genome Phylogeny Estimation

To estimate a robust phylogeny of Rickettsiaceae, 310 perfect OGs (exactly one CDS from each taxon of Rickettsiaceae) were selected for analysis. Multiple sequence alignment of each OG was performed using MUSCLE (default parameters) (Edgar 2004), with regions of poor alignment (length heterogeneous regions) masked using Gblocks (Castresana 2000; Talavera and Castresana 2007). All modified alignments were concatenated into a single data set for phylogeny estimation. Using PhyloBayes v3.3 (Lartillot et al. 2009), we analyzed the data set with the CAT model of substitution, a nonparametric method for modeling site-specific features of sequence evolution (Lartillot and Philippe 2004, 2006). Given the strong base compositional bias of Rickettsiaceae genomes (∼30 %GC), the ability of the CAT model to accommodate saturation due to convergences and reversions (Lartillot et al. 2007) is of substantial importance for estimating rickettsial phylogeny, as demonstrated by us (Driscoll et al. 2013) and others (Rodriguez-Ezpeleta and Embley 2012; Viklund et al. 2012). Two independent Markov chains were run in parallel using PhyloBayes MPI v.1.2e (Lartillot et al. 2013) under the CAT–general time reversible (GTR) model, with the bipartition frequencies analyzed at various time points using the bpcomp program. For tree-building, appropriate burn-in values were determined by plotting the log likelihoods for each chain over sampled generations (time). Analyses were considered complete when the maximum difference in bipartition frequencies between the two chains was less than 0.1. Ultimately, a burn-in value of 1,000, with sampling every two trees, was used to build a consensus tree.

#### Chromosomes

Comparative analysis of the three *R*. *felis* genomes alone was carried out to identify a core genome and determine the composition of the accessory genome. To provide consistency in gene predictions across all three assemblies, we implemented the bacterial gene prediction program fgenesb (Tyson et al. 2004), using the “generic BACTERIAL” model. These annotated assemblies were used for generating OGs, as described above. Results from OG clustering were fit to a three-way Venn diagram to determine the conservation of protein families across all three genomes. Results were further processed manually to identify split open reading frames (ORFs) containing fragments that were not binned into the appropriate OG; left unchecked, this problem inflates the number of predicted ORFs. Adjusted OGs were further subdivided into several categories to facilitate the identification of other artifacts likely stemming from the sequencing technology, particularly poor coverage through repeat regions. To identify extended regions of synteny, a genome sequence alignment was performed using Mauve v.2.3.1, utilizing the “progressiveMauve” option (Darling et al. 2010). The results from OG generation and synteny analysis were used together to evaluate the major differences across all three *R*. *felis* genomes.

The final alignment of the “Rickettsiaceae” OG data set was processed to include only the three *R*. *felis* genomes and the *R*. *akari* genome. This allowed for retention of more sites of the alignment (less sites culled with Gblocks), providing a more robust determination of the divergence pattern of all three *R*. *felis* strains from their closest relative (*R*. *akari*). A phylogeny was estimated under maximum likelihood using RAxML v.7.2.8 (Stamatakis et al. 2008), implementing a gamma model of rate heterogeneity and estimation of the proportion of invariable sites. Branch support was assessed with 1,000 bootstrap pseudoreplications. To determine the degree of divergence of all three strains, only variable positions of the alignment were retained (no. sites = 116 aa) for calculation of an approximation of genome divergence. The program DIVEIN (Deng et al. 2010) was used to estimate percent protein divergence using both the Blosum62 and WAG (Whelan and Goldman) amino acid substitution models.

To determine whether the variation observed in *R*. *felis* str. LSU-Lb might be characteristic of host specialization in booklice, we compared flea-associated strains (strains Cal2 and LSU) with additional booklouse-associated strains. Despite no other sequenced genomes from booklouse-associated *R*. *felis* strains being available for comparison, we utilized several conserved genes (16S rDNA, *gltA*, *sca0*, *sca4*, and *sca5*) sequenced from various *L*. *bostrychophila*-associated *R*. *felis* strains from geographically isolated regions (Arizona, USA and Queensland, Australia) (Behar et al. 2010). Nucleotide alignments for each data set were performed using MUSCLE v3.6 (default parameters), with variable positions identified from the consensus sequence. SNPs were only considered within regions of alignments in which all sequences were represented.

#### Plasmids

Comparative analysis of the four *R*. *felis* pRF plasmid sequences was carried out to evaluate the conservation of genes. The 68 CDS of pRF from *R*. *felis* str. URRWXCal2 were used as a reference, with OGs from all strains mapped on a circular plot using Circos (Krzywinski et al. 2009). An alignment of all four plasmids was performed using Mauve as described above. To determine the relationship of pRF plasmids to other *Rickettsia* plasmids, the sole protein encoded across all sequenced *Rickettsia* plasmids, DnaA-like protein (DALP), was utilized for phylogeny estimation. Rickettsia DALP proteins are extremely variable across homologs except for the C-terminal regions, which encode a DnaA_N domain (pfam11638) typical of chromosome and plasmid replication initiation proteins. The OG including DALP sequences was combined with a sequence from *Orientia tsutsugamushi* encoding a DnaA_N domain (YP_001937581) that was closest to *Rickettsia* DALP sequences in BLASTP analyses. All sequences were aligned using MUSCLE v3.6 (default parameters). A phylogeny was estimated under maximum likelihood using RAxML v.7.2.8 (Stamatakis et al. 2008). A gamma model of rate heterogeneity was used with estimation of the proportion of invariable sites. Branch support was assessed with 1,000 bootstrap pseudoreplications.

An additional contig, c6440, within the assembly of *R*. *felis* str. LSU-Lb was further evaluated as a second divergent plasmid carried by this species. We used fgenesb to predict 38 CDS on c6440, subsequently using these CDS as queries in BLASTP searches. The NR (All GenBank+RefSeq Nucleotides+EMBL+DDBJ+PDB) database was used, coupled with a search against the Conserved Domains Database (Marchler-Bauer et al. 2011). Searches were performed with composition-based statistics across “all organisms, excluding Rickettsiales,” as well as “Rickettsiales.” No filter was used. Default matrix parameters (BLOSUM62) and gap costs (Existence: 11 Extension: 1) were implemented, with an inclusion threshold of 0.005. The predicted 38 CDS, results from BLASTP searches, and GC skew, (G − C)/(G + C), calculated on a fixed 50-nt window, were all mapped on a circular plot using Circos (Krzywinski et al. 2009). An alignment of c6440 with several diverse *Rickettsia* plasmids was performed using Mauve as described above.

Several proteins encoded on c6440 were selected for further analysis. Within a Rickettsiales amplified genetic element (RAGE), 14 proteins encoding components of an F-like type IV secretion system (F-T4SS), were combined with homologs from other rickettsial RAGEs and an F-T4SS from “*Candidatus* Protochlamydia amoebophila” UWE25 and aligned using MUSCLE v3.6 (default parameters). Alignments were concatenated into one data set and used in phylogeny estimation with RAxML as described above. An additional protein (DUF1016) encoded within the RAGE was combined with homologs retrieved from BLASTP searches against several taxon-specific databases: 1) “Rickettsiales,” 2) “*Alphaproteobacteria* (minus Rickettsiales),” 3) “*Proteobacteria* (minus *Alphaproteobacteria*),” 4) “Bacteria (minus *Proteobacteria*),” and 5) “minus Bacteria.” The top 20–50 (query-dependent) subjects from each search resulting in significant (>40 bits) alignments were all compiled, aligned using MUSCLE v3.6 (default parameters), and analyzed with RAxML as described above.

ORFs 38–41 of c6440 were predicted to encode an operon of a large toxin and adjacent repeats-in-toxin (RTX)-like type I secretion system (T1SS). Two proteins homologous to ABC transporters of RTX-like T1SSs were combined with homologs retrieved from BLASTP analyses as described for DUF1016, with a similar protocol for phylogeny estimation. Finally, the large toxin encoded immediately upstream of the RTX-like T1SS (ORF 38) was analyzed using BLASTP searches against the NR database. The Conserved Domains Database was used to identify modular domains at the N- and C-termini, which were collectively used to characterize the protein as a putative toxin.

## Results

### SNP Analysis

Polymorphism across *R. felis* strains was estimated prior to genome assembly. Relative to strain URRWXCal2, 23 and 92 SNPs were calculated for strains LSU and LSU-Lb, respectively (table 1). Estimated SNPs (all 23 for strain LSU, 15 selected SNPs for strain LSU-Lb) were confirmed through sequencing (supplementary table S4, Supplementary Material online). Inspection of the genomic positions of SNPs revealed different patterns of divergence within strains LSU and LSU-Lb relative to str. URRWXCal2. For strain LSU-Lb, 15 SNPs were detected in repeat regions, either within or near *Rickettsia* palindromic elements (RPEs). RPEs are short (∼100–150 nt), selfish elements that insert in intergenic regions and functional RNAs, as well as CDS, wherein ORFs are not disrupted (Ogata et al. 2000). Under a dozen classes of RPEs have been identified in *Rickettsia* genomes (Ogata et al. 2002; Matsutani et al. 2013). In the *R*. *felis* str. LSU-Lb genome, several RPEs are divergent as compared with those from the genomes of flea-associated *R*. *felis* strains, suggesting that RPE insertion and decay are active processes that continue to impact *Rickettsia* genomes. Most of the 71 SNPs occurring in CDS of the *R*. *felis* str. LSU-Lb were found in genes highly conserved across *Rickettsia* genomes (supplementary table S4, Supplementary Material online). Notable exceptions include SNPs in genes encoding two putative effector proteins (RalF, Sca7), a member of the proliferated ProP osmoregulatory transporters, and two proteins on the pRF plasmid.

**Table 1:** Polymorphism across *Rickettsia felis* Strains

	CDS					RNA	IGR	RR	Total
URRWXCal2	FS	Sy	Ns	E	T				
Versus LSU	3	0	9	2	1	0	8	0	23
Versus LSU-Lb	0	28	41	2	0	1	5	15	92

Note.—RNA, ribosomal RNA; IGR, intergenic region; RR, repeat region; FS, frameshift mutation; Sy, synonymous substitution; Ns, nonsynonymous substitution; E, mutation extending CDS; T, nonsense mutation.

For *R*. *felis* str. LSU, half of the estimated SNPs occurred in either one intergenic region or within the ORF encoding an NACHT domain-containing ATPase (pfam05729) that is pseudogenized in all other *Rickettsia* genomes (supplementary table S4, Supplementary Material online). Of the other half of estimated SNPs, five were found in four genes that we previously determined to be commonly associated with mobile elements (Gillespie, Joardar, et al. 2012). These encode a member of the ProP transporter family, the ATP-dependent RNA helicase RhlE, the ribonucleotide translocase Tlc3, and a large (1,179 aa) multidomain protein containing a SpoT-like ppGpp synthetase (SpoT_S) and ankyrin (ANK) repeat domain. The latter protein appears to be a gene fusion unique to *R*. *felis* genomes, as the recently sequenced genome of *R*. *monacensis* str. IrR/Munich has adjacent genes encoding the SpoT (CDI29635) and ANK (CDI29634) domains. Like *R*. *felis* str. LSU-Lb, an SNP was determined in the gene encoding the autotransporter Sca7, although it introduced a frameshift. As Sca7 proteins are not conserved across *Rickettsia* genomes (Blanc et al. 2005), this may indicate a loss of function of this uncharacterized protein relative to other well-studied proteins of the Sca superfamily.

Collectively, the number of SNPs estimated from the sequencing reads generated for *R*. *felis* str. LSU and *R*. *felis* str. LSU-Lb reveal different patterns of divergence relative to the utilized reference genome, *R*. *felis* str. URRWXCal2. As can be expected by their association with the cat flea, *R*. *felis* strains URRWXCal2 and LSU are minimally divergent, with estimated SNPs (all confirmed by sequencing) more prevalent in genes that are derived from lateral gene transfer (LGT). *Rickettsia felis* str. LSU-Lb is more divergent when compared with strain URRWXCal2, with SNPs found predominantly in intergenic regions, repeat regions, and conserved *Rickettsia* genes. The greater divergence in *R*. *felis* str. LSU-Lb is more reflective of genetic drift, likely due to its presumed obligate symbiosis with *L*. *bostrychophila*.

### Genome Architecture

The *R. felis* str. LSU sequencing run yielded 3,036,844 reads which assembled into 10,578 total contigs (737 > 1,000 nt); 7,827 contigs (258 > 1,000 nt) with a top BLASTn match to *Rickettsia* were subsequently ordered and combined into 21 chromosomal and 1 plasmid sequence. In similar fashion, the *R. felis* str. LSU-Lb run yielded 5,463,632 reads which assembled into 59,374 total contigs (1,028 > 1,000 nt); 5,266 *Rickettsia*-like contigs (128 > 1,000 nt) were ordered and combined into 35 chromosomal and 8 plasmid sequences. An additional 52,299 nt sequence, suspected to be a novel plasmid, was identified as a single contig within the initial LSU-Lb assembly and removed prior to ordering. The assembled genomes of *R*. *felis* strains LSU and LSU-Lb are similar to the previously published *R*. *felis* str. URRWXCal2, despite neither assembly resulting in a closed chromosome (table 2). Both LSU and LSU-Lb genomes are comparable to the URRWXCal2 genome in base composition (∼33 %GC) and the presence of the pRF plasmid, as well as encoded stable RNA genes, which have a distinct rickettsial profile (Gillespie, Joardar, et al. 2012). Relative to the URRWXCal2 genome (reannotated with fgenesb, *n* = 1,468), the numbers of predicted ORFs are variable in the LSU (*n* = 1,970) and LSU-Lb (*n* = 1,691) genomes. As described below, these predictions were manually evaluated separately for the chromosomes and pRF plasmids, improving the total numbers of predicted ORFs across all three genomes. The identification of split ORFs that inflated automated ORF predictions, coupled with lack of single contigs despite high coverage for both LSU and LSU-Lb genomes, suggests that the high repeat content of *R*. *felis* genomes is difficult to resolve with current, short-read sequencing technology.

**Table 2:** Sequencing, Assembly, and Annotation Statistics for *Rickettsia felis* Strains LSU and LSU-Lb

Source	Reads		Contigs			Scaffolded	Scaffolds	Final Consensus	%GC	ORFs
	Used	Assembled	No.	Largest	N50					
LSU	3,036,844	2,725,860	10,578	45,231	688	7,827	22	1,546,910	32.82	1,970
chr						6,576	21	1,483,097	32.4	1,859
pRF						1,251	1	63,813	33.24	111
*Wolbachia*						678	438	750,706	35.73	NA
LSU-Lb	5,463,632	2,612,084	59,374	53,712	413	5,266	44	1,579,225	33.15	1,691
chr						2,404	35	1,467,654	32.35	1,576
pRF						2,861	8	59,272	33.76	72
pLbaR						1	1	52,299	33.35	43

Two divergent *Wolbachia* species are known to exist in the LSU *C*. *felis* colony (Pornwiroon et al. 2007), an uncharacterized species and a species closely related to *Wolbachia* spp. previously detected in fleas (Gorham et al. 2003). Infection of fleas with *R*. *felis* str. LSU was associated with the absence of seven other resident microbes identified in *Rickettsia*-uninfected fleas, but not the two *Wolbachia* species (Pornwiroon et al. 2007). Previously, characterization of *R. felis* LSU through the tick-derived ISE6 cell line identified *Wolbachia*-like organisms in the low passage isolate of *R. felis* LSU (Sunyakumthorn et al. 2008). Thus, the generation of *Wolbachia* sequencing data from the ISE6 cell line infected with *R*. *felis* str. LSU was expected. Accordingly, we assembled a complete 16S rDNA sequence that was identical to the 16S rDNA of the uncharacterized *Wolbachia* species (EF121347) previously reported from the LSU colony (supplementary fig. S1, Supplementary Material online). Additionally, we identified 438 contigs that contained genes encoding proteins with top BLASTP hits to *Wolbachia* spp. The characterization of this genome sequence will be reported elsewhere.

No additional microbial genomes were detected in assembling the *R*. *felis* str. LSU-Lb genome; however, one 52.3-kb contig was built that contained mostly plasmid-like genes, yet the profile of these genes, as well as the estimated size of the replicon, did not match plasmid pRF. This putative plasmid is discussed extensively below.

### Phylogenomics

#### Genome Phylogeny Estimation of Rickettsiaceae

**Fig. 1.—:**
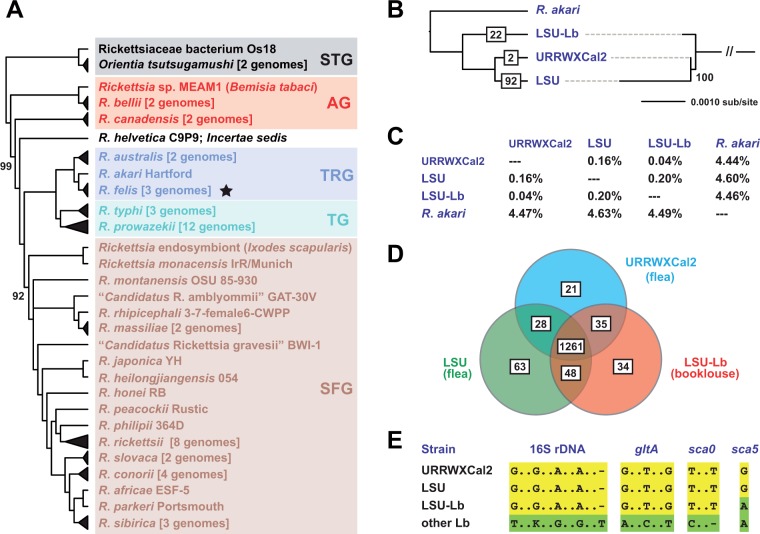
Comparative analysis of *R*. *felis* genomes. (*A*) Genome-based phylogeny estimated for 62 Rickettsiaceae taxa. A total of 310 core proteins, encoded only once in all genomes, were utilized for OG generation, OG alignment (and masking of less conserved positions), and concatenation of aligned OGs (see text). Phylogeny of the final data set (56,920 aa) was estimated using the CAT–GTR model of substitution as implemented in PhyloBayes v3.3 (Lartillot and Philippe 2004, 2006). Tree is a consensus of 3,813 trees (discarding burn-in of 1,000 trees) pooled from two independent Markov chains run in parallel. Branch support (100%, or provided otherwise) was measured through posterior probabilities, which reflect frequencies of clades among the pooled trees. The *R*. *felis* clade is noted with a star. Rickettsiaceae classification scheme follows previous studies (Gillespie et al. 2007; Gillespie, Nordberg, et al. 2012; Driscoll et al. 2013). Complete taxon names, PATRIC genome IDs (Gillespie et al. 2011; Wattam et al. 2014), and genome statistics are provided in supplementary table S2, Supplementary Material online. (*B*) Phylogeny estimated from a second alignment of the abovementioned 310 core proteins, exclusive to the three *R*. *felis* genomes and *R*. *akari* (221,668 aa). Cladogram at left shows the branching pattern with unique positions in boxes. Phylogram at right shows the branch lengths for the three *R*. *felis* lineages. (*C*) Approximation of *R*. *felis* genome divergence. Calculated % divergence (116 informative aa sites from 310 core proteins) with DIVEIN (Deng et al. 2010), using the WAG (top) and Blosum62 (bottom) amino acid substitution models. *Rickettsia akari* str. Hartford was used as the outgroup. (*D*) Distribution of OGs generated across *R*. *felis* genomes. A further description of the breakdown of all input proteins from each genome, as well as results from manual evaluation, is provided in supplementary figure S2, Supplementary Material online. (*E*) Comparison of *R*. *felis* strains URRWXCal2, LSU, and LSU-Lb with other strains of *R*. *felis* identified from booklice. Yellow, SNPs uniting *R*. *felis* str. LSU-Lb with strains URRWXCal2 and LSU; green, SNPs uniting *R*. *felis* str. LSU-Lb with other booklouse-associated strains. Full alignments illustrating the positions of SNPs are provided in supplementary figure S4, Supplementary Material online.

Phylogeny estimation of 310 conserved proteins encoded in 62 Rickettsiaceae genomes illustrates the placement of *R*. *felis* strains, together with *R*. *akari* and *R*. *australis*, within the TRG rickettsiae (fig. 1*A*). In response to earlier reports classifying *R*. *felis* as belonging to either TG (Higgins et al. 1996) or SFG (Bouyer et al. 2001) rickettsiae, we created the TRG to account for the “transitional” lineage between these long standing *Rickettsia* groups (Gillespie et al. 2007). Although standard models of amino acid substitution (e.g., WAG, LG) group TRG and SFG rickettsiae as a monophyletic clade (Gillespie et al. 2008; Gillespie, Joardar, et al. 2012), our recent use of the CAT model demonstrated for the first time the monophyly of TG and TRG rickettsiae (Driscoll et al. 2013). The phylogeny estimation presented here also recovered the monophyly of TG and TRG rickettsiae, with the CAT substitution model accounting for similar base composition shared by TRG and SFG rickettsiae genomes (32.5 %GC) relative to the more AT-rich genomes of TG rickettsiae (29 %GC) (supplementary table S2, Supplementary Material online). Collectively, robust genome-based phylogeny estimation unambiguously supports the derivation of three *Rickettsia* groups (TG, TRG, and SFG) from the poorly understood AG rickettsiae, consistent with unique host specialization and genome characteristics that define each distinct lineage (Gillespie et al. 2008).

#### Genomic Heterogeneity in Strains of R. felis

##### Genome Sequence Evolution

Consistent with the results from SNP analysis, phylogeny estimation recovered the split of *R*. *felis* str. LSU-Lb from the flea-associated strains (fig. 1*B*). Unexpectedly, far more divergence separated the flea-associated strains relative to the SNP analysis; this is partially a consequence of our somewhat conservative SNP calling routine but also may be a reflection of the shorter reads and poorer assembly of *R*. *felis* str. LSU relative to *R*. *felis* str. URRWXCal2, which was assembled into one chromosome (table 2). Estimations of amino acid divergence illustrate that, despite sharing common ancestry relative to *R*. *felis* str. LSU-Lb, the flea-associated strains are highly divergent, with str. LSU evolving more rapidly subsequent to the split from *R*. *felis* str. LSU-Lb (fig. 1*C*). Nonetheless, compared with *R*. *akari*, it is evident that all three *R*. *felis* strains collectively comprise a species that is clearly distinct from other members of TRG rickettsiae (∼4.5% aa divergence).

##### Genome Architecture Conservation

OGs of proteins generated across all three *R*. *felis* genomes resulted in a core genome encoding 1,261 proteins (fig. 1*D*). Despite this, only roughly half (46.8%) of these conserved proteins are of uniform length across all three genomes, reflecting the unequal distribution of RPEs and other insertion elements, as well as minor discrepancies in gene start sites (supplementary fig. S2, Supplementary Material online). Still, a manual assessment of generated OGs revealed that, relative to *R*. *felis* str. URRWXCal2, split and truncated proteins from str. LSU (*n* = 347) and str. LSU-Lb (*n* = 109) grossly contributed to overestimation of the accessory genome (supplementary fig. S2, Supplementary Material online). This likely reflects the shorter reads generated by next-generation sequencing, in conjunction with the high repeat density of *R*. *felis* genomes, preventing a portion of genes to assemble properly. Once adjusted for, the accessory genome revealed similar numbers for proteins unique to each genome, as well as proteins shared by any two combinations (fig. 1*D*).

An approximation of synteny conservation across the closed genome of *R*. *felis* str. URRWXCal2 and the multicontig assemblies of strains LSU and str. LSU-Lb revealed substantial colinearity across all three genomes (supplementary fig. S3, Supplementary Material online). In conjunction with the minimal accessory genome, minor deviations in synteny can be attributed to differential numbers of transposases and repeat regions, which are probable factors preventing closure of the LSU and str. LSU-Lb genomes. It is likely that such duplicate transposases and other insertion sequences have mediated several homologous recombination events resulting in minor synteny breaks across these genomes; such has been observed in *Rickettsia* (Felsheim et al. 2009; Gillespie, Joardar, et al. 2012) and other Rickettsiales (Gillespie, Nordberg, et al. 2012) genomes with elevated genes encoding transposases (and inactive derivatives) and other repeat elements. Notwithstanding, the three *R*. *felis* genomes are highly conserved in relation to other *Rickettsia* genomes, with the defining characteristics of *R*. *felis* str. URRWXCal2 (e.g., split *sca0*, presence of *sca3*, duplicate *pat1*, elevated toxin–antitoxin modules, split *ppcE*; presence of both *rickA* and *ralF*, etc.) conserved in the LSU and str. LSU-Lb genomes (data not shown).

##### Lack of Booklouse-Associated SNPs

Five highly conserved *Rickettsia* genes (16S rDNA, *gltA*, *sca0*, *sca4*, and *sca5*) were utilized for comparing *R*. *felis* flea-associated strains (URRWXCal2 and LSU) with *R*. *felis* str. LSU-Lb and other *L*. *bostrychophila*-associated strains (supplementary fig. S4, Supplementary Material online). Importantly, these other booklouse-associated strains are from regions (Arizona, USA and Queensland, Australia) geographically isolated from the LSU *L*. *bostrychophila* colony (Behar et al. 2010). Surprisingly, of 11 SNPs identified from four genes (*sca4* was invariant), all but one (in *sca5*) united *R*. *felis* str. LSU-Lb with the flea-associated genomes to the exclusion of the other *L*. *bostrychophila*-associated *R*. *felis* strains (fig. 1*E*), indicating a potential host shift to *L*. *bostrychophila* that occurred independent of the geographically isolated *L*. *bostrychophila*-associated *R*. *felis* strains.

##### pRF Plasmids

**Fig. 2.—:**
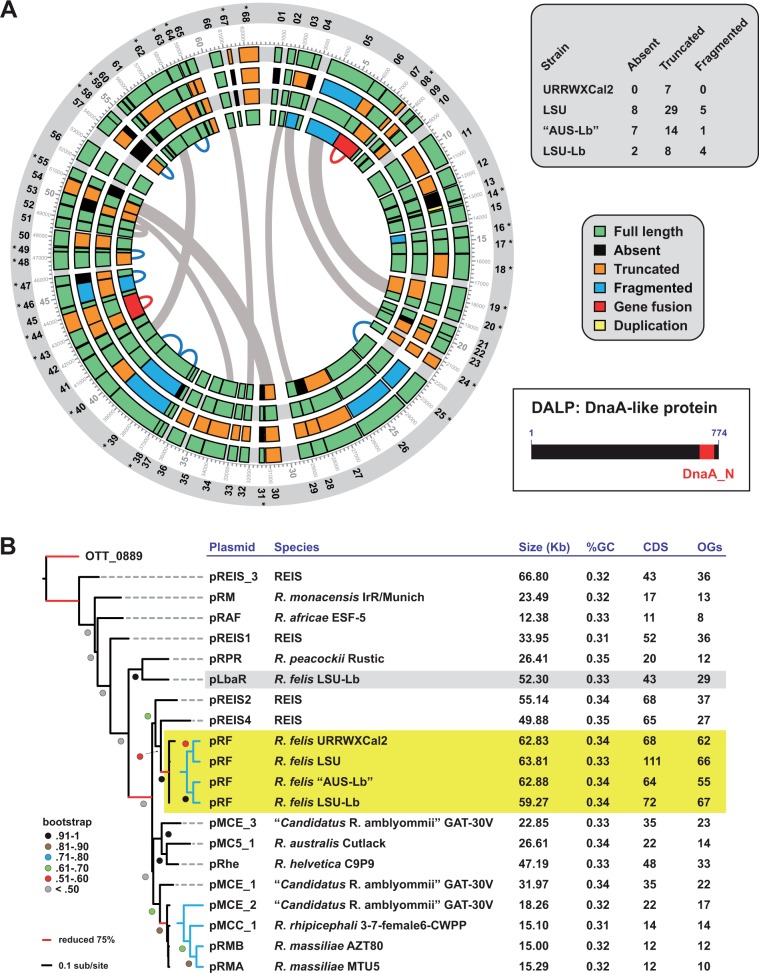
Phylogenomic analysis of diverse pRF plasmids. (*A*) Comparative analysis of four diverse pRF plasmids. pRF of *R*. *felis* str. URRWXCal2 (NC_007110) was used as a reference to evaluate the conservation of its 68 CDS on the pRF plasmids of strains LSU, “AUS-Lb” (GQ329881), and LSU-Lb. See text for details on OG generation and analysis. Outer ring depicts the 68 CDS beginning at 12 o’clock and continuing clockwise. CDS denoted with asterisks depict truncated ORFs relative to larger homologs from other bacterial genomes. Ring 2: Scale with coordinates (5-kb intervals shown in larger numbers) as originally defined for pRF of *R*. *felis* str. URRWXCal2 (Ogata, Renesto, et al. 2005). Rings 3–6: CDS encoded on plasmid pRF of *R*. *felis* strains URRWXCal2, LSU, “AUS-Lb,” and LSU-Lb. Inset at top-right describes the CDS color scheme. Inset at center-right provides the total CDS absent, truncated or fragmented for each strain. CDS with significant similarity are connected by thick gray arcs across the interior of the plot. Thin blue arcs depict split ORFs that are conserved across all four plasmids. Thin red arcs depict two instances where adjacent CDS are fused as one CDS in strain LSU-Lb. Annotations for the 68 pRF CDS are provided in supplementary figure S6, Supplementary Material online. Plot made using Circos (Krzywinski et al. 2009) with manual adjustment. Inset at bottom-right shows the prototype DALP utilized for phylogeny estimation shown in panel (*B*). (*B*) Phylogeny estimation of DALPs encoded on 20 diverse *Rickettsia* plasmids. Hypothetical protein OTT_0889 (*O. tsutsugamushi* str. Ikeda) was used as an outgroup. For highlighting: Yellow, pRF plasmids; gray, plasmid pLbaR. See text for alignment and tree-building methods. Tree is final optimization likelihood: (−16,490.366606) using WAG substitution model with GAMMA and proportion of invariant sites estimated. Brach support is from 1,000 bootstrap pseudoreplications. Plasmid statistics were compiled from PATRIC, NCBI or from analyses in this study. “OGs” refers to the number of proteins that were included in OGs generated across all 20 *Rickettsia* plasmids.

Both LSU and LSU-Lb strains of *R*. *felis* carry the pRF plasmid, which was originally identified in strain URRWXCal2 (Ogata, Renesto, et al. 2005) and subsequently shown to vary minimally in an *R*. *felis* strain associated with *L*. *bostrychophila* from Australia (Behar et al. 2010), hereafter referred to as str. “AUS-Lb.” A comparison of all four pRF plasmids illustrates limited conservation in CDS (fig. 2*A*) but high synteny (supplementary fig. S5, Supplementary Material online). A core set of 54 CDS is present across all four plasmids, yet many genes are either split or truncated in one or more plasmids. Only 15 CDS are conserved in length across all four plasmids (supplementary fig. S6, Supplementary Material online). One of these, pRF_56, was originally annotated as a hyaluronidase, an enzyme thought to facilitate pathogen spread through host tissues. Characterization of a similar protein from *Streptococcus pyogenes* did not reveal a hyaluronidase function, but rather beta-*N*-acetylglucosaminidase activity, with the ability to remove beta-*O*-linked *N*-acetylglucosamine from mammalian glycoproteins (Sheldon et al. 2006). As homologs of pRF_56 are unknown from other *Rickettsia* genomes, these proteins may be a factor unique to the biology of *R*. *felis*.

We previously noted that one particular pRF-encoded hypothetical protein, pRF_68, is conserved in the chromosomes of TG rickettsiae, but variably present in other *Rickettsia* genomes (Gillespie et al. 2007). Thus, pRF_68 is one a few factors strongly conserved in insect-associated *Rickettsia* genomes (Ammerman et al. 2009; Gillespie, Ammerman, Beier-Sexton, et al. 2009). Homologs of pRF_68 have since been identified on the pMC5_1 plasmid of *R*. *australis* str. Cutlack (YP_005389775), as well as on the chromosomes of *Rickettsia bellii* str. RML369-C (YP_538411), *Rickettsia* endosymbiont of *Ixodes scapularis* (REIS) (EER21570), and *R*. *monacensis* str. IrR/Munich (CDI29587). Remarkably, a homolog (CDG49776) is also encoded on the chromosome of the obligate intracellular bacterium *Cardinium* cBtQ1 (Bacteroidetes), a facultative endosymbiont of the whitefly *Bemisia tabaci* (Santos-Garcia et al. 2014). These results further bolster our original observations that plasmid pRF is a modular array of LGTs exchanged with other *Rickettsia* spp. as well as other divergent bacteria (Gillespie et al. 2007).

Two gene fusions were identified in pRF of *R*. *felis* str. LSU-Lb, but not str. “AUS-Lb,” consistent with the near dearth of shared SNPs in the conserved chromosomal genes (discussed above). Despite this, a phylogeny of conserved pRF genes grouped the *L*. *bostrychophila*-associated strains together relative to the pRF plasmids of strains LSU and URRWXCal2 (data not shown). Consistent with this, a phylogeny estimation of DALPs, the sole protein encoded across all sequenced *Rickettsia* plasmids as revealed by our OG generation, grouped strains LSU-Lb and “AUS-Lb” and strains LSU and URRWXCal2 as sister clades (fig. 2*B*). Thus, despite considerable decay in gene content across pRF plasmids, these extrachromosomal elements share a common origin and have diverged in tandem with their respective chromosomes.

#### R. felis str. LSU-Lb Carries a Second Unique Plasmid

Inspection of the *R*. *felis* str. LSU-Lb assembly revealed a single contig comprised many plasmid-like genes. This contig, hereafter referred to as plasmid of *L*. *bostrychophila* associated *Rickettsia* (pLbaR), encodes 43 predicted proteins, including a ParA gene typically present on *Rickettsia* plasmids (supplementary fig. S7, Supplementary Material online). Multiple attempts to circularize this putative plasmid through PCR were unsuccessful, potentially due to transposases flanking the large contig. Despite this, the contig size (52.3 kb) and %GC (33) of pLbaR are typical of other *Rickettsia* plasmids, and 29 predicted proteins were grouped into OGs in a clustering analysis across 20 *Rickettsia* plasmids (fig. 2*B*). Importantly, the DALP encoded on pLbaR is most closely related to the DALP from plasmid pRPR (*Rickettsia peacockii*), which is substantially divergent from pRF plasmids (Felsheim et al. 2009), supporting pLbaR as a unique *Rickettsia* plasmid (fig. 2*B*).

##### pLbaR Integrative Conjugative Element

**Fig. 3.—:**
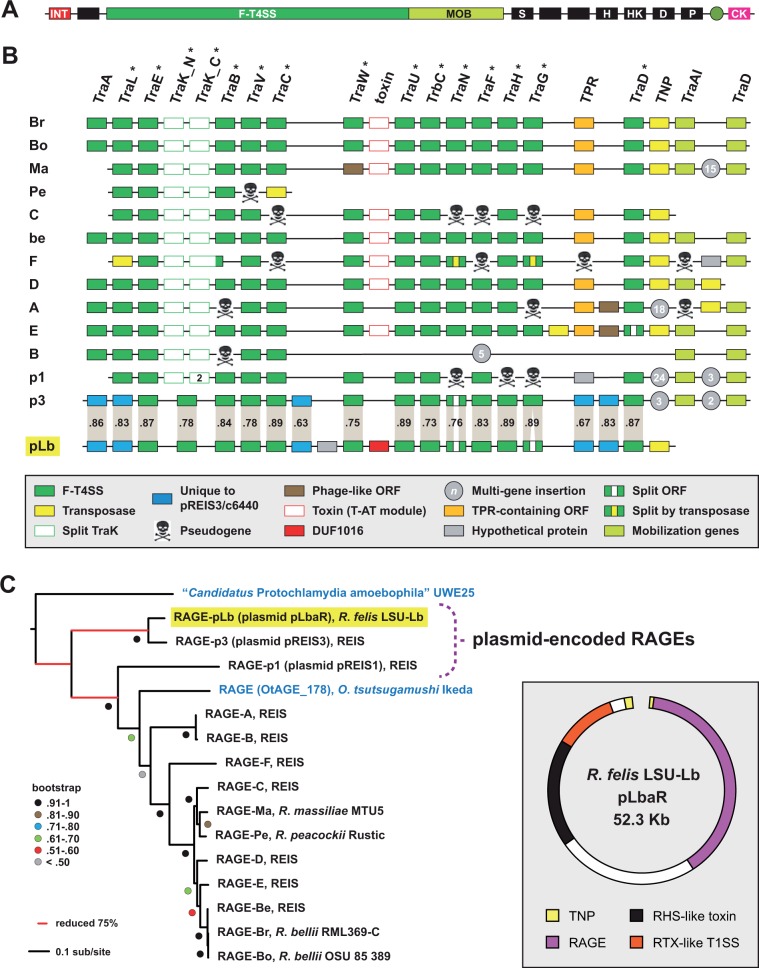
Plasmid pLbaR of *R*. *felis* LSU-Lb carries an integrative conjugative element. (*A*) The prototypical RAGE (Gillespie, Joardar, et al. 2012). Gene models are colored as follows: Red, integrase; black, nonconjugative; green, F-T4SS; light green, mobilization; olive circle, tRNA Val-GAC; magenta, cytidyl kinase. Conserved nonconjugative genes as follows: S, stringent response synthetase; H, stringent response hydrolase; HK, histidine kinase; D, DNA methyltransferase (D12 class N6 adenine-specific); P, *Dna*I-like primase. (*B*) Schema of conjugative (F-T4SS and mobilization) gene organization within 14 RAGEs. RAGE symbols at left are described in panel (*C*). RAGEs p3 and pLb are compared (% aa identity) as indicated with gray shading. Gene color and symbols are described in the inset. Phylogeny estimation of DUF1016 proteins is provided in supplementary figure S8, Supplementary Material online. (*C*) Phylogeny estimation of 14 F-T4SS proteins illustrated in panel (*B*) with an asterisk. Pseudogenes were not included. Branch support (posterior probabilities) is from Bayesian analysis (see text for details). All *Rickettsia* species are colored black, with “*Candidatus* Protochlamydia amoebophila” UWE25 and *O. tsutsugamushi* Ikeda colored blue. Inset at right: General schema of plasmid pLbaR of *R*. *felis* LSU-Lb (see supplementary fig. S7, Supplementary Material online, for further information).

Remarkably, ORFs 2–23 of pLbaR encode an integrative conjugative element, named RAGE, known from the genomes of some rickettsial species (Gillespie, Joardar, et al. 2012). The prototypical RAGE comprised genes encoding an F-T4SS, a mobilization module, nonconjugative proteins with a variety of predicted functions, and an integrase that facilitates insertion into tRNA genes (fig. 3*A*). RAGEs are extensively proliferated and highly pseudogenized in the genomes of the Scrub Typhus agent, *Orientia tsutsugamiushi* (Cho et al. 2007; Nakayama et al. 2008), yet usually occur in a single insertion site (tRNA Val-GAC) in *Rickettsia* genomes; for example, *R*. *bellii* (Ogata et al. 2006) and *Rickettsia massiliae* (Blanc et al. 2007). Aside from an extraordinary nine chromosomal RAGEs, the REIS genome contains two additional RAGEs each on two of its four plasmids (pREIS1 and pREIS3) (Gillespie, Joardar, et al. 2012). Although the RAGE encoded on pLbaR contains only the F-T4SS genes, it shares near perfect colinearity with the RAGE encoded on plasmid pREIS3 (fig. 3*B*). Thus, *R*. *felis* str. LSU-Lb is the second *Rickettsia* species harboring a plasmid with an RAGE, lending further insight on the dynamic nature of these fascinating mobile elements.

The RAGEs of pLbaR and pREIS3 share five genes unknown from other RAGEs, and are also the only RAGEs with the N- and C-terminal domains of TraK (a VirB9 homolog) fused into a single protein (fig. 3*B*). This underscores the common origin of these elements, despite *R*. *felis* and REIS being well diverged from one another in the estimated phylogeny of Rickettsiaceae (fig. 1*A*). Relative to most chromosomal encoded RAGEs, the plasmid-encoded RAGEs of REIS lack a toxin-encoding gene between the genes coding for TraU and TraW; on pLbaR, a unique gene encoding a protein DUF1016 is found in this position. DUF1016 proteins belong to a diverse family of restriction endonucleases (Kinch et al. 2005). BLASTP analysis identified duplicate DUF1016 proteins encoded in the chromosomes of *R*. *bellii* strains and all three *R*. *felis* strains, as well as a single protein encoded in the REIS genome. Phylogeny estimation grouped all of these chromosomal-encoded proteins in a clade with other alphaproteobacterial homologs, including uncharacterized phage proteins encoded in several *Wolbachia* genomes (supplementary fig. S8, Supplementary Material online). Remarkably, the DUF1016 protein of pLbaR grouped with a homolog from *Legionella longbeachae* (WP_003636576) in a separate clade well diverged from that containing the alphaproteobacterial DUF1016 proteins. This LGT underscores the modular nature of RAGEs (Gillespie, Joardar, et al. 2012).

Phylogeny estimation of 14 F-T4SS proteins encoded within 14 *Rickettsia* RAGEs further bolsters the close relatedness of the pLbaR and pREIS3 RAGEs, and indicates the three plasmid encoded RAGEs are divergent from the chromosomal encoded RAGEs (fig. 3*C*). Relative to the F-T4SS of “*Candidatus* Protochlamydia amoebophila” UWE25, the plasmid encoded RAGEs branch off even before an RAGE encoded in the *O*. *tsutsugamushi* Ikeda genome, illustrating that multiple invasions of divergent RAGEs have occurred within *Rickettsia* genomes throughout evolution. The presence of RAGEs on plasmids from two *Rickettsia* species indicates that plasmids are the likely vehicles by which RAGEs spread across *Rickettsia* genomes. Their incorporation into *Rickettsia* chromosomes indicates that RAGE invasions are an active process shaping the composition of rickettsial genomes, equipping recipients with many genes important for survival within eukaryotic cells (Gillespie, Joardar, et al. 2012).

##### pLbaR T1SS Operon

**Fig. 4.—:**
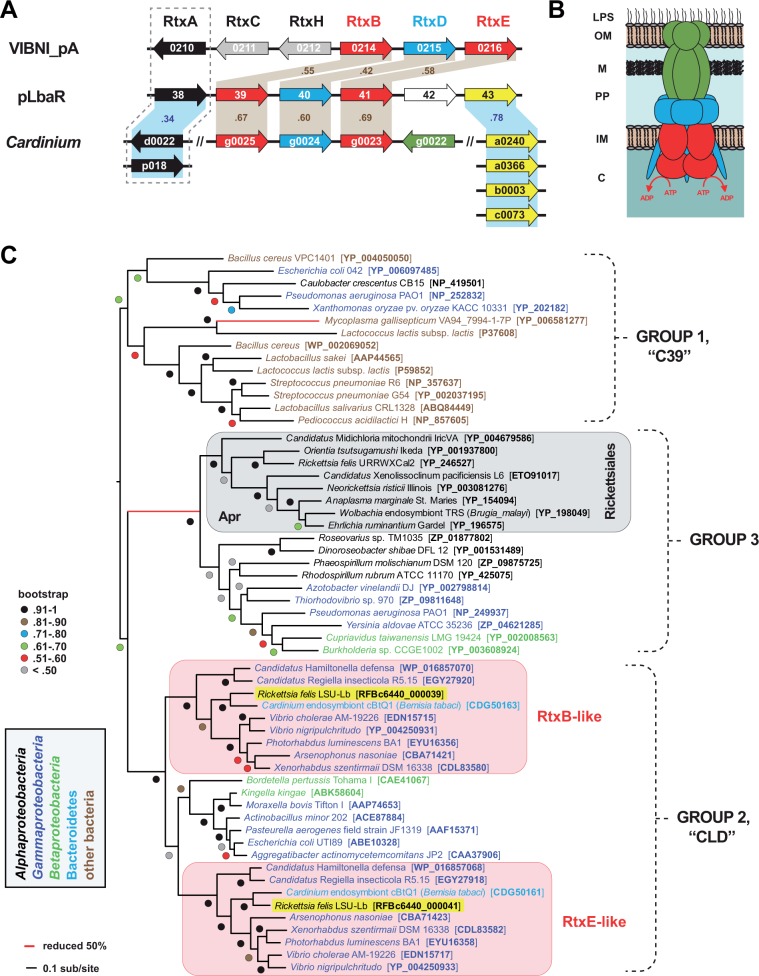
Plasmid pLbaR of *R*. *felis* str. LSU-Lb encodes an RTX-like T1SS. (*A*) Schema comparing the structure of the RTX T1SS and associated genes from plasmid VIBNI_pA of *Vibrio nigripulchritudo* (NC_015156) with T1SSs and associated genes from plasmid pLbaR of *R. felis* str. LSU-Lb, and also the chromosome and plasmid pcBtQ1 of *Cardinium* endosymbiont cBtQ1 of *Bemisia tabaci* (CBQZ010000007). Tan shading indicates high similarity (%ID) across T1SSs. Blue shading illustrates similarity (%ID) across *Rickettsia* and *Cardinium* only. Gene colors as follows: Red, T1SS ABC transporters; blue, T1SS membrane fusion proteins (MFP); green outer membrane efflux protein (OEP); gray, RTX-specific proteins; white, hypothetical protein; yellow, PD-(D/E)XK nuclease family transposase (pfam12784); black, toxins. A dashed box illustrates the close association of the MARTX (*V*. *nigripulchritudo*) and RHS-like (*R*. *felis*) toxins with the T1SS; the RHS-like toxins of *Cardinium* are not linked to the T1SS, but share similarity with the RHS-like toxin of *R*. *felis* (supplementary fig. S9, Supplementary Material online). (*B*) Model for the canonical T1SS illustrating the tripartite structure formed by the trimeric OEP (green), IM/periplasmic MFP (blue), and dimeric IM ABC transporter (red). Bridging the asymmetry between the OEP and ABC transporter oligomers, the MFP is shown as a putative hexamer with the N-terminal sequences in the cytoplasm (Lee et al. 2012). All sequenced Rickettsiales genomes encode an Apr-like T1SS (Gillespie 2014); the distinct RTX-like T1SS of *R*. *felis* str. LSU-Lb is predicted to utilize a second ABC transporter, characteristic of atypical RTX-T1SSs (Boardman and Satchell 2004; Lee et al. 2008; Li et al. 2008). (*C*) Phylogeny estimation of T1SS ABC transporters. Three clades of ABC transporters correspond to a recent classification (Kanonenberg et al. 2013). The gray box illustrates the Group 3 ABC transporter (AprD-like) of the T1SS encoded within all Rickettsiales genomes (Gillespie 2014). Within the Group 2 transporter clade, the duplicate ABC transporters of RTX and RTX-like T1SSs are within red boxes, with the sequences encoded on plasmid pLbaR highlighted yellow. See text for alignment and tree-building methods. Tree is final optimization likelihood: (−59,413.119327) using WAG substitution model with GAMMA and proportion of invariant sites estimated. Brach support is from 1,000 bootstrap pseudoreplications.

ORFs 39–41 of plasmid pLbaR encode components of a T1SS most similar to the RTX-T1SSs (fig. 4). Like RTX-T1SSs of *Vibrio* spp., ORFs 39 and 41 encode ABC transporters that flank a membrane fusion protein (ORF40) (fig. 4*A*). In *Vibrio* RTX-T1SSs, secretion of the large MARTX toxin requires two ABC transporters, RtxB and RtxE (Boardman and Satchell 2004; Lee et al. 2008; Li et al. 2008), and it has been suggested that these proteins may form a heterodimer within the RTX T1SS complex (Boardman and Satchell 2004). Importantly, ORFs 39–41 are distinct from the Apr-like Rickettsiales T1SS (Gillespie 2014), which contains one ABC transporter and one membrane fusion protein that presumably assemble with TolC to form a functional secretion channel (fig. 4*B*). Remarkably, BLASTP analysis of ORFs 39–41 revealed highest similarity to a *Cardinium* cBtQ1 T1SS operon that is duplicated and thought to have arisen from an LGT event with *Vibrio* spp. (Santos-Garcia et al. 2014). Compared with *Cardinium* RTX-like T1SS operons, a TolC homolog is not encoded on pLbaR; however, the chromosomal TolC protein that is highly conserved and functional in *Rickettsia* genomes (Kaur et al. 2012) would serve as the outer membrane channel component of this RTX-like T1SS. The presence of a flanking gene (ORF 43) encoding a PD-(D/E)XK nuclease family transposase (pfam12784) that shares highest similarity to four transposases in the *Cardinium* cBtQ1 genome strongly implies LGT of these RTX-like T1SS operons between these divergent obligate intracellular bacteria.

Phylogeny estimation of T1SS ABC transporters grouped the two RTX-like ABC transporters of pLbaR within the Group 2 transporter clade (fig. 4*C*). Given that the conserved rickettsial AprD-like transporters belong to the Group 3 transporter clade, this is the first report of an RTX-like T1SS from any species of Rickettsiales. Within the Group 2 transporter clade, the duplicate ABC transporters of RTX and RTX-like T1SSs form separate clades (RtxB and RtxE), and assemblage within each clade indicates substantial LGT across divergent obligate intracellular bacteria and other *Gammaproteobacteria*, including species of *Vibrio*. Aside from *R*. *felis* and *Cardinium* cBtQ1, three gammaproteobacterial endosymbionts of insects (“*Candidatus* Hamiltonella defensa,” “*Candidatus* Regiella insecticola R5.15,” and *Arsenophonus nasoniae*) have acquired RTX-like T1SSs, despite reductive genomes due to obligate intracellular lifestyles. As RTX-T1SSs and associated MARTX toxins are known from *Vibrio* plasmids (Le Roux et al. 2011), it is likely that LGTs have equipped these obligate intracellular endosymbionts with a common secretion apparatus and associated toxin(s), which are likely factors for adaptation to specific host environments.

##### pLbaR RHS-Like Toxin

On pLbaR, immediately upstream of the RTX-like T1SS operon lies a gene (ORF 38) encoding a large protein of 3,241 aa, hereafter referred to as pLbaR_38 (fig. 4*A*). Given that large RTX toxins are often encoded nearby the RTX T1SS genes, we investigated whether pLbaR_38 had characteristics of RTX toxins. RTX proteins are highly modular and extraordinarily diverse in protein domain composition across species, with a wide range of ascribed functions (Linhartova et al. 2010). Nevertheless, these toxins are almost always secreted by T1SSs, and also contain hallmark Gly/Asp-rich nonapeptide repeat sequences near the C-terminus (Satchell 2011). pLbaR_38 is not a classical RTX toxin, as it lacks Gly/Asp-rich nonapeptide repeat sequences near its C-terminus (data not shown). However, BLASTP analysis revealed the protein to be modular, having substantially different matches across specific regions of the protein. Three distinct domains were identified: 1) An NT (N-terminal) (pos. 22–180) OTU (Ovarian tumor)-like cysteine protease domain, pfam02338 (Makarova et al. 2000), 2) an internal (pos. 2813–2924) salivary gland secreted toxin (Tox-SGS) domain, pfam15651 (Arca et al. 2005; Korochkina et al. 2006), and 3) a C-terminal ANK repeat domain (pos. 3079–3161), which contains two ANK repeats (supplementary fig. S9, Supplementary Material online). Tox-SGS domains are associated with recombination hot spot (RHS) proteins of insects and crustaceans, with domain architecture indicative of an origin from bacterial polymorphic toxin systems (Zhang et al. 2012). Large bacterial proteins with RHS domains function as insecticidal toxins (Joo Lee et al. 2004) as well as bacteriocins in intercellular completion (Koskiniemi et al. 2013). Accordingly, pLbaR_38 is best classified as an RHS-like toxin.

Remarkably, pLbaR_38 shares significant similarity with two RHS-like proteins encoded in the *Cardinium* cBtQ1 genome (fig. 4*A*), consistent with the close relatedness of the RTX-like T1SSs of these two species. The internal sequence of this RHS-like toxin also shares similarity with proteins encoded in the genomes of some *Wolbachia* spp, very few *Rickettsia* spp., *Diplorickettsia massiliensis*, and *Orientia tsutsugamishi* (supplementary fig. S9, Supplementary Material online). Although none of these proteins has characterized functions, it is clear that diverse sequences have recombined into the large ORF that encodes pLbaR_38. Furthermore, minimal regions within pLbaR_38 and the two RHS-like toxins of *Cardinium* cBtQ1 have low similarity with RTX-like toxins encoded in the genomes of “*Candidatus* Hamiltonella defensa” and “*Candidatus* Regiella insecticola” (data not shown), consistent with the similar RTX-like T1SSs common to all of these genomes. Interestingly, a nearby ORF on pLbaR encoding a hypothetical protein of 614 aa (pLbaR_36) is similar (22% ID) to a protein (NP_966396) encoded in the genome of the *Wolbachia* endosymbiont of *Drosophila melanogaster* (*w*Mel). Curiously, in the *w*Mel genome, NP_966396 is directly adjacent to an ORF encoding a protein (NP_966397) with similarity to pLbaR_38, indicating that a multigene transfer has likely occurred between *w*Mel and pLbaR. This is also reflected by the ANK repeats encoded by pLbaR_38, which are most similar to dozens of ANK-repeat domain containing proteins encoded within many *Wolbachia* genomes, some of which also encode an OTU-like cysteine protease domain like pLbaR_38 (data not shown). Collectively, pLbaR_38 is a highly modular protein containing eukaryotic-like domains and regions similar to proteins encoded in other intracellular bacterial genomes. Its close proximity to an RTX-like T1SS suggests a potential mechanism for secretion of this large toxin by *R*. *felis* LSU-Lb into its booklouse host.

## Discussion

In arthropods, vertical (inherited) versus horizontal transmission of rickettsial species is believed to play an important role in determining whether mutualistic or parasitic interactions evolve (Werren 1997); therefore, vertical transmission favors the evolution of benign and mutualistic associations, whereas frequent horizontal (infectious) transmission favors virulent rickettsiae (Hackstadt 1996). Thus, the level of virulence is expected to depend on the relative importance of vertical and horizontal transmission for maintenance of the rickettsiae within arthropod host populations. Yet, the constituents of the complex interplay between *Rickettsia* spp. and arthropod hosts, and also the checkpoint at which species diverge into pathogens or symbionts, are ill defined. Flea-borne transmission of *R*. *felis* has proved challenging to characterize (Bouyer et al. 2001; Ogata, Renesto, et al. 2005) as both horizontal (Hirunkanokpun et al. 2011) and vertical (Wedincamp and Foil 2002) transmission mechanisms are employed. Also, similar to *R*. *typhi*, *R*. *felis* is shed in flea feces (Reif et al. 2011), possibly providing an alternative avenue for vertebrate host infection. The unique dualistic nature of *R*. *felis*, in which distinct strains employ different transmission routes for sustained infection of arthropod populations, makes this an ideal model organism for studying rickettsial determinants of transmission and pathogenicity.

### R*.* felis *Genomic Heterogeneity*

Despite earlier reports of negligible variation across strains of *R*. *felis* (Pornwiroon et al. 2006; Thepparit et al. 2011), we determined SNPs in both *R*. *felis* str. LSU and *R*. *felis* str. LSU-Lb, in relation to the published URRWXCal2 strain, suggesting that spatial isolation (LSU) and potential host specialization (LSU-Lb) have resulted in genetic divergence (table 1). *Rickettsia felis* str. LSU-Lb had four times more SNPs than *R*. *felis* str. LSU, yet the majority of these polymorphisms were found in intergenic regions, repeat regions, and conserved *Rickettsia* genes. In contrast, the majority of SNPs determined for *R*. *felis* str. LSU were found in genes previously identified as dynamic components of the *Rickettsia* mobilome, particular those encoding proliferated protein families involved in adaptations to intracellular lifestyle (Gillespie, Joardar, et al. 2012). Thus, although both of the strains we sequenced are divergent from the published URRWXCal2 strain, each new genome paints a different portrait of the selective forces operating on *R*. *felis* genomes from strains associated with different arthropod vectors. Importantly, the intraspecific variation we report here for *R*. *felis* strains will add to the list of markers used to distinguish *R*. *felis* and *R*. *felis*-like organisms (Jiang et al. 2013; Odhiambo et al. 2014).

### Chromosome and pRF Plasmid Divergence

Consistent with the SNP analysis, analyses of the assembled genomes suggest that *R*. *felis* str. LSU-Lb is divergent from the flea-associated strains (fig. 1*B* and *C*). Unexpectedly, assembly of the *R*. *felis* str. LSU genome revealed more divergence not witnessed by initial SNP estimation, though manual assessment of gene predictions revealed many split genes and gene fragments that appear to be artifacts of sequencing and assembly. Such artifacts were far less observed for *R*. *felis* str. LSU-Lb. One possible reason for the difficulty encountered with the *R*. *felis* str. LSU sequencing and assembly is the presence of many *Wolbachia* reads in the generated data. Another is a higher instance of repeat regions and encoded transposases in *R*. *felis* str. LSU relative to *R*. *felis* str. LSU-Lb. Notwithstanding, future sequencing efforts, ideally utilizing a combination of short- and long-read sequencing methods, will be needed to resolve the incomplete genes models within both *R*. *felis* str. LSU and *R*. *felis* str. LSU-Lb genomes, as well as to evaluate the few missing genes that are present in the *Rickettsia* core genome and/or the *R*. *felis* str. URRWXCal2 genome.

Despite neither assembly of the LSU and LSU-Lb genomes resulting in single chromosomes (table 2), manual curation allowed for an approximation of a core *R*. *felis* genome (1,261 genes) and dynamic accessory genome (fig. 1*D*). Relative to *R*. *felis* str. URRWXCal2, both LSU and LSU-Lb genomes lacked 21 genes, which are all probable pseudogenes (average size of 80 aa) relative to other *Rickettsia* homologs. Similar profiles were found for genes shared by *R*. *felis* str. URRWXCal2 and either LSU or LSU-Lb strain, indicating a pseudogene-laden accessory genome characteristic of rickettsial reductive genome evolution (Andersson et al. 1998). Additionally, singletons encoded within the LSU and LSU-Lb genomes are also probable pseudogenes, having much larger counterparts in other *Rickettsia* genomes. Collectively, cursory analysis of the composition and divergence of the chromosomes of all three strains of *R*. *felis* does not reveal any specific factors that might account for host specialization.

As with chromosomes, the composition and degree of pseudogenization across pRF plasmids from four *R*. *felis* strains (URRWXCal2, LSU, “AUS-Lb,” and LSU-Lb) does not show a pattern consistent with strict divergence between flea- and booklouse-associated strains (fig. 2*A*). Since its discovery in 2005 as the first plasmid from an obligate intracellular bacterium (Ogata, Renesto, et al. 2005), virtually nothing is known regarding the function of the 68 proteins encoded on pRF, other than the expression of two of its small heatshock proteins (Ogawa et al. 2007). Our comparative analysis of four complete pRF plasmids from diverse strains of *R*. *felis* revealed some insight on the conservation of genes that are potentially critical for the biology of *R*. *felis* (fig. 2). However, like the chromosomes of all three strains, the pRF plasmids do not contain any particular distinct features that might account for host specialization.

### pLbaR: A Factor Mediating Host Specialization?

**Fig. 5.—:**
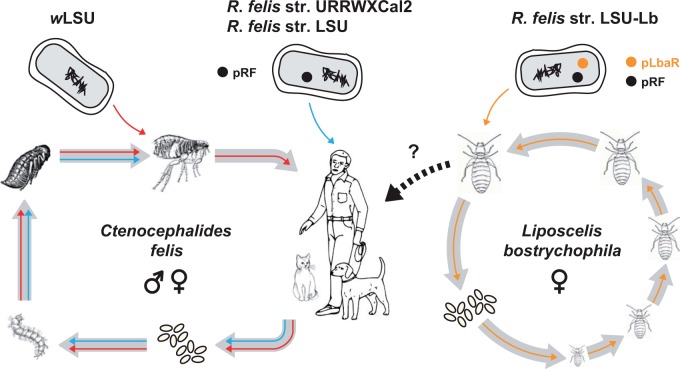
Modes of infection and transmission of divergent *R*. *felis* strains in fleas and booklice. As opportunistic pathogens, *R*. *felis* strains URRWXCal2 and LSU are shown infecting vertebrate hosts (center). Such infection would occur by a blood-feeding arthropod (e.g., tick or mite) or by flea-fecal contamination of bites on the skin. The life cycle of the cat flea, *C. felis*, is shown (left), with an adult insect harboring a *Wolbachia* symbiont (top left). After feeding on a vertebrate host that is infected with *R*. *felis*, eggs are laid that contain both *Wolbachia* and *R*. *felis*. Both parasites are transmitted transstadially throughout the holometabolous life cycle (egg–larva–pupa–adult). Adults feed again on an uninfected vertebrate host, with transovarial transmission of *R*. *felis* and *Wolbachia*. The life cycle of the parthenogenic booklouse, *L. bostrychophila*, is shown (right), with an adult female infected by *R*. *felis* str. LSU-Lb. Rickettsia are transmitted transovarially and transstadially throughout the stages of the paurometabolous life cycle (egg–nymphal stages (1–4)–adult). Although rickettsial infection of *L*. *bostrychophila* occurs at 100% frequency throughout populations across the world, superinfection by different *R*. *felis* strains is plausible given the presence of rickettsia in ovaries, oocytes, fat bodies, and digestive tracts. Such superinfection is predicted to occur due to the niche overlap of *C*. *felis* and *L*. *bostrychophila*, with booklice feeding on dead fleas and flea eggs that contain other strains of *R*. *felis*. The ability of *R*. *felis* str. LSU-Lb. to infect vertebrates (dashed arrow) is unknown, but would involve inhalation or dermal inoculation of booklouse feces (see text for further details).

The discovery of a second, unique plasmid (pLbaR) in the *R*. *felis* str. LSU-Lb assembly provides a set of unique genes that might play a role in this strain’s unique biology with *L*. *bostrychophila* (fig. 5). *Rickettsia felis* str. LSU-Lb is the third *Rickettsia* genome identified that carries multiple divergent plasmids, with the genomes of REIS and “*Candidatus* Rickettsia amblyommii” GAT-30V carrying four and three distinct plasmids, respectively. Additionally, this is the second report of a genome harboring a plasmid that encodes an RAGE. Although RAGEs tend to carry genes encoding DNA methyltransferases (D12 class), PolC-like DNA helicases, stringent response hydrolases and synthetases, and proteins containing histidine kinase and tetratricopeptide domains, many other genes with diverse functions are known to piggyback on these elements (Gillespie, Joardar, et al. 2012). Adding to this list is the DUF1016 protein encoded within the RAGE of pLbaR. Proteins annotated as DUF1016 are similar to the uncharacterized protein YhcG of *Escherichia coli*, which has been grouped in the PD-(D/E)XK phosphodiesterase superfamily (Kosinski et al. 2005; Steczkiewicz et al. 2012). Bacterial DUF1016 proteins are known from other mobile elements, for example, the integrative conjugative elements of *Enterococcus* spp. and *Clostridium difficile* that carry vancomycin resistance (Garnier et al. 2000; Sebaihia et al. 2006). The closest homolog to the pLbaR-encoded DUF1016 protein is from another intracellular species, *Legionella longbeachae*, consistent with previous reports identifying LGT between species of *Legionella* and *Rickettsia* (Cox et al. 2004; Ogata et al. 2006; Gillespie, Ammerman, Dreher-Lesnick, et al. 2009; Gillespie et al. 2010; Gillespie, Joardar, et al. 2012). The presence of divergent DUF1016 proteins in the genomes of several different *Wolbachia* spp. might hint at a common role these proteins serve rickettsiae in their arthropod hosts.

The pLbaR operon encoding the RTX-like T1SS and associated RHS-like toxin also likely arose from LGT, as the *Cardinium* endosymbiont cBtQ1 genome encodes highly similar homologs to all of these proteins. Gene exchanges between *Rickettsia* spp. and *Cardinium* endosymbionts of diverse arthropod species have previously been reported. Originally described from pseudogenes of *R*. *peacockii* (Simser et al. 2005), the transposase ISRPe1 is sporadically encoded across *Rickettsia* genomes (Felsheim et al. 2009; Gillespie, Joardar, et al. 2012), yet highly similar proteins are encoded across species of *Cardinium* and *Rickettsia*, as well as some other intracellular bacterial species (Duron 2013). We previously reported the first occurrence of an entire biotin synthesis operon on a bacterial plasmid, pREIS2 of REIS, with phylogeny estimation indicating that this unique operon was also found in the chromosomes of *Neorickettsia* spp. and the deltaproteobacterium *Lawsonia intracellularis* (Gillespie, Joardar, et al. 2012). This same operon was subsequently found in the genome of the *Cardinium hertigii*, an endosymbiont of the wasp *Encarsia pergandiella*, despite a near lack of genes encoding all other major biosynthesis pathways (Penz et al. 2012). Remarkably, the same biotin operon was recently found in the genome of the *Wolbachia* endosymbiont of the bedbug *Cimex lectularius* (*w*Cle), with *w*Cle provisioning biotin to its insect host in an established obligate mutualism (Nikoh et al. 2014).

Other LGTs were also predicted between *Rickettsia* spp. and *Cardinium hertigii* (Penz et al. 2012), as were dozens of exchanges between *Rickettsia* spp. and the amoeba symbiont “*Candidatus* Amoebophilus asiaticus” (Schmitz-Esser et al. 2010; Gillespie, Joardar, et al. 2012), another member of the Bacteroidetes that is closely related to *Cardinium* spp. (Santos-Garcia et al. 2014). These findings are reflective of intracellular bacterial species occupying similar niches within their eukaryotic hosts, the so-called “intracellular arena” (Bordenstein and Wernegreen 2004). *Cardinium* and *Rickettsia* coinfect whiteflies (Chiel et al. 2007; Skaljac et al. 2010, 2013), and both were detected together in the bacteriocyte, as well as in other host tissues (Skaljac et al. 2010). This co-occurrence increases opportunities for genetic exchange, with factors such as the RAGE (Gillespie, Joardar, et al. 2012) and bacteriophage (Bordenstein and Wernegreen 2004; Degnan and Moran 2008) facilitating LGT.

Our estimated phylogeny of T1SS ABC transporters suggests that, along with the *Cardinium* endosymbiont cBtQ1, several gammaproteobacterial arthropod endosymbionts utilize a similar RTX-like T1SS to deliver substrates into their intracellular environments. The genomes of *A. nasoniae* (Wilkes et al. 2010), “*Candidatus* Hamiltonella defensa” (Degnan et al. 2009), and “*Candidatus* Regiella insecticola R5.15” (Hansen et al. 2012) all encode RTX toxins in addition to these specialized T1SSs. Regarding *R*. *insecticola* 5.15, its RTX-like T1SS and RTX toxins are among several pathogenicity factors that are absent in a closely related strain, *R*. *insecticola* LSR1, which unlike strain 5.15 does not protect host pea aphids from attack by the parasitic wasp *Aphidius ervi* (Hymenoptera: Braconidae) (Hansen et al. 2012). Thus, it is likely that RTX-like T1SSs of these endosymbionts are utilized to secrete substrates that serve as facilitators of intracellular survival, acting directly against species that harm the host. An overall role in equipping resident symbionts with tools to outcompete other microbes and maintain a consistent microbiota is also probable for RTX and RHS substrates, as has recently been suggested for elevated genes encoding these toxins in the genomes of honey bee endosymbionts (Kwong et al. 2014).

Although the type VI secretion system (T6SS) has been implicated in the secretion of some bacterial RHS proteins (Koskiniemi et al. 2013), the lack of T6SS genes in the genomes of *Cardinium* endosymbiont cBtQ1 and *R*. *felis* str. LSU-Lb suggests another means for the putative secretion of their RHS proteins. The RTX-like T1SSs encoded in both genomes would serve to facilitate secretion of such large substrates, especially given that T6SS substrates are typically smaller in size than large RHS proteins (Silverman et al. 2012). Collectively, the operon encoding the RTX-like T1SS and associated RHS-like toxin, as well as the DUF1016 gene piggybacking on the RAGE, are the major factors encoded on pLbaR that distinguish the *R*. *felis* str. LSU-Lb genome from the genomes of the flea-associated *R*. *felis* strains. These factors are promising for investigating the genetic mechanisms that differentiate *R*. *felis* as an obligate mutualist of *L*. *bostrychophila* from *R*. *felis* as a facultative parasite of fleas and other arthropods (Behar et al. 2010). Given that *R*. *felis* str. LSU-Lb lacks nearly all SNPs unique to other *L*. *bostrychophila*-associated *R*. *felis* strains (fig. 1*E*), it is imperative to determine whether these strains also carry plasmid pLbaR, or contain different factors that are associated with independent colonization(s) of *L*. *bostrychophila*.

### TRG Rickettsiae and Reproductive Parasitism

Since its discovery, *R*. *felis* has been difficult to sort in the traditional TG/SFG rickettsiae classification scheme, consistent with it possessing attributes of both groups. Based on 1) its phylogenetic position (ancestral to SFG rickettsiae), 2) its predominant association with insects, and 3) substantial LGT of pRF plasmid genes with species of AG rickettsiae, *R*. *felis* was grouped together with *R*. *akari* and *R*. *australis* in the TRG rickettsiae (Gillespie et al. 2007). Subsequent phylogenomics analysis supported TRG rickettsiae as a distinct rickettsial lineage (Gillespie et al. 2008). Although convincing evidence for minimal LGT between *R*. *typhi* and *R*. *felis* has been shown (Merhej et al. 2011), hundreds of genes in the core *Rickettsia* genome possess a strong pattern of vertical descent (Gillespie et al. 2008; Hernandez-Lopez et al. 2013). Along with our recent report (Driscoll et al. 2013), the CAT model-based phylogeny estimation of the core *Rickettsia* genome presented here supports the common ancestry of TG and TRG rickettsiae, refuting the classification of *R*. *felis* as a member of SFG rickettsiae (fig. 1).

Relative to TG (body lice and fleas) and SFG (ticks) rickettsiae, a broader host range has been reported for TRG rickettsiae (e.g., ticks, mites, fleas, booklice, other insects) (Gillespie, Nordberg, et al. 2012). Although little is known regarding the nature of the relationships that species of TRG rickettsiae have with their hosts, it is clear that multiple reproductive parasites have emerged within the lineage of *R*. *felis*, *R*. *felis*-like organisms, and the recently described “*Candidatus* Rickettsia asemboensis” (Jiang et al. 2013). For the wasp *Neochrysocharis formosa* (Hymenoptera: Eulophidae), which is an endoparasitoid of the leafminer *Liriomyza trifolii* (Diptera: Agromyzidae), an uncharacterized *Rickettsia* species has been shown to induce thelytokous parthenogenesis (Hagimori et al. 2006). A BLASTN search with the 16S rDNA of this uncharacterized species (AB185963) yields the best match to 16S rDNA of *R*. *felis* str. URRWXCal2 (1,341/1,346 identity). Interestingly, other members of this lineage have been reported from the Birch Catkin Bug, *Kleidocerys resedae* (Hemiptera: Lygaeidae) (Matsuura et al. 2012), as well as several species of the weevil genera *Curculio*, *Koreoculio,* and *Archarius* (Coleoptera: Curculionidae) (Toju and Fukatsu 2011; Toju et al. 2013). The effects of these uncharacterized species on host fitness and reproductive mode are unknown and warrant future investigation.

For unnamed strains of *R*. *felis* infecting *L*. *bostrychophila*, reproductive parasitism has evolved into obligate mutualism, supported by 1) a paucity of males known in nature, 2) the near 100% rickettsial infection rate, and 3) no viable males after eliminating rickettsia (Yusuf and Turner 2004; Perotti et al. 2006). Despite SNP analyses revealing it is a divergent strain (fig. 1*E*), the lack of males in our LSU *L*. *bostrychophila* colony, coupled with a 100% infection rate, suggests *R*. *felis* str. LSU-Lb is also an obligate mutualist, though this needs to be substantiated. It is unknown how exactly *R*. *felis* contributes to parthenogenesis, other than having a probable role in oocyte development (Perlman et al. 2006). In stark contrast, *R*. *felis* has never been implicated in the induction of parthenogenesis in the cat flea, and it is clear that *C*. *felis* does not undergo parthenogenesis in the absence of males (Zakson-Aiken et al. 1996). Thus, *R*. *felis* strains URRWXCal2 and LSU are best described as facultative parasites of fleas, with unknown potential as reproductive parasites of other arthropods.

For several *Rickettsia* species belonging to ancestral lineages, the mechanisms that govern parthenogenesis (Perotti et al. 2006; Giorgini et al. 2010), male killing (Werren et al. 1994; Lawson et al. 2001; von der Schulenburg et al. 2001; Majerus and Majerus 2010), and feminization (Zchori-Fein et al. 2006) are unknown, though future sequencing of the genomes of these organisms may yield potential factors that promote reproductive parasitism. However, genome sequence data may not readily reveal these factors, as a recent attempt to identify common factors in *Cardinium* and *Wolbachia* species that are involved in cytoplasmic incompatibility proved futile (Penz et al. 2012). Although several factors encoded on plasmid pLbaR of *R*. *felis* str. LSU-Lb were also identified in the genomes of some *Cardinium* and *Wolbachia* species, their presence in the genomes of other *Rickettsia* reproductive parasites remains to be determined.

### Pathogenicity of *R. felis* str. LSU-Lb?

Aside from plasmid pLbaR, the minimal genomic divergence distinguishing *R*. *felis* str. LSU-Lb from flea-associated strains suggests that it has the potential to be a human pathogen (fig. 5). While provisional, our hypothesis that different strains of *R*. *felis* have independently colonized *L*. *bostrychophila* is consistent with a report of liposcelids feeding on dead insects and insect eggs (Turner 1994). Considering that *L*. *bostrychophila* occupies common niches with fleas, such as bird and rat nests, as well as canine fur (Mockford 1971; Baz 1990; Turner 1994), dead fleas and flea eggs would serve as prime sources for the acquisition of *R*. *felis*. Although primary endosymbionts of arthropods are nearly always found in specialized organs (mycetomes), *R*. *felis* is found in a variety of tissues throughout *L*. *bostrychophila*, including single cell mycetocytes and an organ-forming mycetome (Perotti et al. 2006; Thepparit et al. 2011). For this early stage of evolutionary transition from a facultative parasite into an obligate endosymbiont, vertical transmission may still be periodically supplemented with horizontal transmission. This scenario best explains why *R*. *felis* LSU-Lb lacks nearly all SNPs unique to other *L*. *bostrychophila*-associated *R*. *felis* strains, yet possesses the same host–symbiont relationship as these strains relative to the facultative parasitic nature of the flea-associated strains.

As a closely related lineage to parasitic lice (Grimaldi and Engel 2006), liposcelids have an intimate association with human habitats (Baz 1990), and have been observed feeding on nails (Lin et al. 2004) and infesting scalps (Burgess et al. 1991). The distribution of rickettsia in the liposcelid digestive tract and fat bodies (Chapman 2003; Perotti et al. 2006) indicates that bacteria may be present in host feces, similar to that reported for flea-associated *R*. *felis* (Reif et al. 2011) and *R*. *typhi* (Azad and Beard 1998; Reif and Macaluso 2009). Thus, although *R*. *felis* str. LSU-Lb is a probable obligate mutalist of a nonhematophagous arthropod, its potential to be horizontally transmitted to vertebrates needs to be addressed. As a corollary, another critical aspect to determine is whether or not *R*. *felis* str. LSU-Lb can infect fleas. If both of these scenarios are possible, then in theory fleas could acquire *R*. *felis* str. LSU-Lb by feeding on infected vertebrates. To this end, the potential for *R*. *felis* str. LSU-Lb to disrupt sexual reproduction in fleas, as well as other arthropod vectors, has profound implications for infections disease research and arthropod pest management.

## Supplementary Material

Supplementary tables S1–S4 and figures S1–S9 are available at *Genome Biology and Evolution* online (http://www.gbe.oxfordjournals.org/).

Supplementary Data
