# Supplementary material for: Genomic Diversification in Strains of Rickettsia felis Isolated from Different Arthropods
Source: Genome Biol Evol. 2014 Dec 4;7(1):35–56. doi: 10.1093/gbe/evu262 (PMC4316617; doi:10.1093/gbe/evu262)
Supplement: Supplementary Data [file supp_7_1_35__index.html]

Genomic Diversification in Strains of Rickettsia felis Isolated from Different Arthropods — Supplementary Data 

# Genomic Diversification in Strains of *Rickettsia felis* Isolated from Different Arthropods

## Supplementary Data

files

**Files in this Data Supplement:**

- Supplementary Data - pdf file
- Supplementary Data - pdf file
- Supplementary Data - docx file
- Supplementary Data - xlsx file
