# Supplementary material for: Genomic Diversification in Strains of Rickettsia felis Isolated from Different Arthropods
Source: Genome Biol Evol. 2014 Dec 4;7(1):35–56. doi: 10.1093/gbe/evu262 (PMC4316617; doi:10.1093/gbe/evu262)
Supplement: Supplementary Data [file supp_evu262_Supp_Mat_Figs_1-9.pdf]

Supplementary fig. S1. Characteristics of the *Wolbachia* 16S rRNA gene concurrently sequence and assembled with *R. felis* str. LSU.

(A) Assembled sequence of the 16S rRNA gene of the *Wolbachia* species concurrently sequenced with *Rickettsia felis* str. LSU. Two divergent *Wolbachia* species are known to exist in the LSU *C. felis* colony (Pornwiroon, et al. 2007), an uncharacterized species and a species closely related to *Wolbachia* spp. previously detected in fleas (Gorham, et al. 2003). The sequence assembled here is identical to the 16S rDNA of the uncharacterized *Wolbachia* species (EF121347) previously reported from the LSU cat flea colony.

(B) Alignment showing the identical match of the assembled *Wolbachia* 16S rDNA to the uncharacterized *Wolbachia* species previously reported from the LSU cat flea colony. Alignment was performed using MUSCLE v3.6 (default parameters) (Edgar 2004). Asterisks denote identical nucleotides.

Literature Cited

Edgar RC 2004. MUSCLE: multiple sequence alignment with high accuracy and high throughput. Nucleic Acids Res 32: 1792-1797. doi: 10.1093/nar/gkh340
32/5/1792 [pii]

Gorham CH, Fang QQ, Durden LA 2003. *Wolbachia* endosymbionts in fleas (Siphonaptera). The Journal of parasitology 89: 283-289. doi: 10.1645/0022-3395(2003)089[0283:WEIFS]2.0.CO;2

Pornwiroon W, Kearney MT, Husseneder C, Foil LD, Macaluso KR 2007. Comparative microbiota of *Rickettsia felis*-uninfected and -infected colonized cat fleas, *Ctenocephalides felis*. The ISME journal 1: 394-402. doi: 10.1038/ismej.2007.38

A

```
>wLSU 16S SSU rDNA, full-length
TTTTTCATAGTAATATCCCTAAGTAGATGTATATTTTATATATTAATGCTTTTGATTTGCAAAAACATATTGACACTGTGATTATATCACATAATAATATTTTTTTAAGTTGTTTGAATAGTTGAAGATTAAAATA
TTTCTAGAGTAGAAATAAAGGCAGCAGTATAAAGCTAAATTATTTTTTTATCGGATTTATCCGATGTACCCTCAACCTTTGATAGGTTGAAGTCTAAATTTGAGAGTTTGATCCTGGCTCAGAATGAACGCTGGCGG
CAGGCCTAACACATGCAAGTCGAACGAGGTTATATTGTAGCTTGCTATAGTATAACTTAGTGGCAGACGGGTGAGTAATGTATAGGAATCTACCTAGTAGTACGGGACAATTGCTGGAAACGGCAACTAATACCGT
ATACGCCCTACGGGGGAAAGATTTATTGCTATTAGATGAGCCTATATTAGATTAGTTAGTTGGTGGGGTAATGGCCTACCAAGGCAATGATCTATAGCTGATCTGAGAGGATGATCAGCCACACTGGAACTGAGAT
ACGGTCCAGACTCCTACGGGAGGCAGCAGTGGGGAATATTGGACAATGGGCGAAAGCCTGATCCAGCCATGCCGCATGAGTGAAGAAGGCCTTTGGGTTGTAAAGCTCTTTCAGTGAGGAAGATAATGACGGTACT
CACAGAAGAAGTCCTGGCTAACTCCGTGCCAGCAGCCGCGGTAATACGGAGAGGGCTAGCGTTATTCGGAATTATTGGGCGTAAAGGGCGCGTAGGCTGATTAGTAAGTTAAAAGTGAAATCCCAAAGCTTAACTT
TGGAATTGCTTTTAAAACTGCTAATCTAGAGATTGAAAGAGGATAGAGGAATTCCTAGTGTAGAGGTGAAATTCGTAAATATTAGGAGGAACACCAGTGGCGAAGGCGTCTATCTGGTTCAAATCTGACGCTGAGG
CGCGAAGGCGTGGGGAGCAAACAGGATTAGATACCCTGGTAGTCCACGCTGTAAACGATGAATGTTAAATATGGGGAGTTTACTTTCTGTATTACAGCTAACGCGTTAAACATTCCGCCTGGGGACTACGGTCGCA
AGATTAAAACTCAAAGGAATTGACGGGGACCCGCACAAGCGGTGGAGCATGTGGTTTAATTCGATGCAACGCGAAAAACCTTACCACTCCTTGACATGGAAATTATACCTATCCGAAGGGATGGGGTCGGTTCGGC
CGGATTTCACACAGGTGTTGCATGGCTGTCGTCAGCTCGTGTCGTGAGATGTTGGGTTAAGTCCCGCAACGAGCGCAACCCTCATCCTTAGTTACCATCAGGTAATGCTGGGAACTTTAAGGAAACTGCCAGTGAT
AAACTGGAGGAAGGTGGGGATGATGTCAAGTCATCATGGCCCTTATGGAGTGGGCTACACACGTGCTACAATGGTGGCTACAATGGGCTGCAAAGTCGCGAGGCTAAGCTAATCCCTTAAAAGCCATCTCAGTTCG
GATTGTACTCTGCAACTCGAGTGCATGAAGTTGGAATCGCTAGTAATCGTGGATCAGCATGCCACGGTGAATACGTTCTCGGGTCTTGTACACACTGCCCGTCACGCCATGGGAATTGGTTTCACTCGAAGCTAAT
GACCTAACCGCAAGGAGGGAGTTATTTAAAGTGGGATCAGTGACTGGGGTGAAGTCGTAACAAGGTAGCAGTAGGGGAATCTGCAGCTGGATTACCTCCTTAGGCTTTGTGCATACTTACTGCTTTTGATAGTGGT
GTTGTTATGCACTATACTGCTGTCTTTACTTCTACTTTATTTTGATCTCCCTATTCTAATTTCCTGGTAATGGAATTTTATGAGTGATAGGCCTCTTTCTCCACATTTACAAATCTATAAAGTACAAGTTACTAGT
TTTTTTCTCTATTATGCACAGGTTAACTGGTACCTTGCTGTTTCTTTTATTAATTGTATTTTTCTTGGTATTTCATATTACATGTTTATTTTCCTAGACTATTTATAGTGAAATACTTAA
```

B

```
wCfe        TTTTTCATAGTAATATCCCTAAGTAGATGTATATTTTATATATTAATGCTTTTGATTTGCAAAAACATATTGACACTGTGATTATATCACATAATAATATTTTTTTAAGTTGTTTGAATA
EF121347    ------------------------------------------------------------------------------------------------------------------------

wCfe        GTTGAAGATTAAAATATTTCTAGAGTAGAAATAAAGGCAGCAGTATAAAGCTAAATTATTTTTTTATCGGATTTATCCGATGTACCCTCAACCTTTGATAGGTTGAAGTCTAAATTTGAGA
EF121347    ------------------------------------------------------------------------------------------------------------------------

wCfe        GTTTGATCCTGGCTCAGAATGAACGCTGGCGGCAGGCCTAACACATGCAAGTCGAACGAGGTTATATTGTAGCTTGCTATAGTATAACTTAGTGGCAGACGGGTGAGTAATGTATAGGAA
EF121347    -----------------AATGAACGCTGGCGGCAGGCCTAACACATGCAAGTCGAACGAGGTTATATTGTAGCTTGCTATAGTATAACTTAGTGGCAGACGGGTGAGTAATGTATAGGAA
                             *******************************************************************************************************

wCfe        TCTACCTAGTAGTACGGGACAATTGCTGGAAACGGCAACTAATACCGTATACGCCCTACGGGGGAAAGATTTATTGCTATTAGATGAGCCTATATTAGATTAGTTAGTTGGTGGGGTAAT
EF121347    TCTACCTAGTAGTACGGGACAATTGCTGGAAACGGCAACTAATACCGTATACGCCCTACGGGGGAAAGATTTATTGCTATTAGATGAGCCTATATTAGATTAGTTAGTTGGTGGGGTAAT
            ************************************************************************************************************************

wCfe        GGCCTACCAAGGCAATGATCTATAGCTGATCTGAGAGGATGATCAGCCACACTGGAACTGAGATACGGTCCAGACTCCTACGGGAGGCAGCAGTGGGGAATATTGGACAATGGGCGAAAG
EF121347    GGCCTACCAAGGCAATGATCTATAGCTGATCTGAGAGGATGATCAGCCACACTGGAACTGAGATACGGTCCAGACTCCTACGGGAGGCAGCAGTGGGGAATATTGGACAATGGGCGAAAG
            ************************************************************************************************************************

wCfe        CCTGATCCAGCCATGCCGCATGAGTGAAGAAGGCCTTTGGGTTGTAAAGCTCTTTCAGTGAGGAAGATAATGACGGTACTCACAGAAGAAGTCCTGGCTAACTCCGTGCCAGCAGCCGCG
EF121347    CCTGATCCAGCCATGCCGCATGAGTGAAGAAGGCCTTTGGGTTGTAAAGCTCTTTCAGTGAGGAAGATAATGACGGTACTCACAGAAGAAGTCCTGGCTAACTCCGTGCCAGCAGCCGCG
            ************************************************************************************************************************

wCfe        GTAATACGGAGAGGGCTAGCGTTATTCGGAATTATTGGGCGTAAAGGGCGCGTAGGCTGATTAGTAAGTTAAAAGTGAAATCCCAAAGCTTAACTTTGGAATTGCTTTTAAAACTGCTAA
EF121347    GTAATACGGAGAGGGCTAGCGTTATTCGGAATTATTGGGCGTAAAGGGCGCGTAGGCTGATTAGTAAGTTAAAAGTGAAATCCCAAAGCTTAACTTTGGAATTGCTTTTAAAACTGCTAA
            ************************************************************************************************************************

wCfe        TCTAGAGATTGAAAGAGGATAGAGGAATTCCTAGTGTAGAGGTGAAATTCGTAAATATTAGGAGGAACACCAGTGGCGAAGGCGTCTATCTGGTTCAAATCTGACGCTGAGGCGCGAAGG
EF121347    TCTAGAGATTGAAAGAGGATAGAGGAATTCCTAGTGTAGAGGTGAAATTCGTAAATATTAGGAGGAACACCAGTGGCGAAGGCGTCTATCTGGTTCAAATCTGACGCTGAGGCGCGAAGG
            ************************************************************************************************************************

wCfe        CGTGGGGAGCAAACAGGATTAGATACCCTGGTAGTCCACGCTGTAAACGATGAATGTTAAATATGGGGAGTTTACTTTCTGTATTACAGCTAACGCGTTAAACATTCCGCCTGGGGACTA
EF121347    CGTGGGGAGCAAACAGGATTAGATACCCTGGTAGTCCACGCTGTAAACGATGAATGTTAAATATGGGGAGTTTACTTTCTGTATTACAGCTAACGCGTTAAACATTCCGCCTGGGGACTA
            ************************************************************************************************************************

wCfe        CGGTCGCAAGATTAAAACTCAAAGGAATTGACGGGGACCCGCACAAGCGGTGGAGCATGTGGTTTAATTCGATGCAACGCGAAAAACCTTACCACTCCTTGACATGGAAATTATACCTAT
EF121347    CGGTCGCAAGATTAAAACTCAAAGGAATTGACGGGGACCCGCACAAGCGGTGGAGCATGTGGTTTAATTCGATGCAACGCGAAAAACCTTACCACTCCTTGACATGGAAATTATACCTAT
            ************************************************************************************************************************

wCfe        CCGAAGGGATGGGGTCGGTTCGGCCGGATTTCACACAGGTGTTGCATGGCTGTCGTCAGCTCGTGTCGTGAGATGTTGGGTTAAGTCCCGCAACGAGCGCAACCCTCATCCTTAGTTACC
EF121347    CCGAAGGGATGGGGTCGGTTCGGCCGGATTTCACACAGGTGTTGCATGGCTGTCGTCAGCTCGTGTCGTGAGATGTTGGGTTAAGTCCCGCAACGAGCGCAACCCTCATCCTTAGTTACC
            ************************************************************************************************************************

wCfe        ATCAGGTAATGCTGGGAACTTTAAGGAAACTGCCAGTGATAAACTGGAGGAAGGTGGGGATGATGTCAAGTCATCATGGCCCTTATGGAGTGGGCTACACACGTGCTACAATGGTGGCTA
EF121347    ATCAGGTAATGCTGGGAACTTTAAGGAAACTGCCAGTGATAAACTGGAGGAAGGTGGGGATGATGTCAAGTCATCATGGCCCTTATGGAGTGGGCTACACACGTGCTACAATGGTGGCTA
            ************************************************************************************************************************

wCfe        CAATGGGCTGCAAAGTCGCGAGGCTAAGCTAATCCCTTAAAAGCCATCTCAGTTCGGATTGTACTCTGCAACTCGAGTGCATGAAGTTGGAATCGCTAGTAATCGTGGATCAGCATGCCA
EF121347    CAATGGGCTGCAAAGTCGCGAGGCTAAGCTAATCCCTTAAAAGCCATCTCAGTTCGGATTGTACTCTGCAACTCGAGTGCATGAAGTTGGAATCGCTAGTAATCGTGGATCAGCATGCCA
            ************************************************************************************************************************

wCfe        CGGTGAATACGTTCTCGGGTCTTGTACACACTGCCCGTCACGCCATGGGAATTGGTTTCACTCGAAGCTAATGACCTAACCGCAAGGAGGGAGTTATTTAAAGTGGGATCAGTGACTGGG
EF121347    CGGTGAATACGTTCTCGGGTCTTGTACACACTGCCCGTCACGCCATGGGAATTGGTTTCACTCGAAGCTAATGACCTAACCGCAAGGAGGGAGTTATTTAAAGTGGGATCAGTGACTGGG
            ************************************************************************************************************************

wCfe        GTGAAGTCGTAACAAGGTAGCAGTAGGGGAATCTGCAGCTGGATTACCTCCTTAGGCTTTGTGCATACTTACTGCTTTTGATAGTGGTGTTGTTATGCACTATACTGCTGTCTTTACTTC
EF121347    GTG---------------------------------------------------------------------------------------------------------------------
            ***

wCfe        TACTTTATTTTGATCTCCCTATTCTAATTTCCTGGTAATGGAATTTTATGAGTGATAGGCCTCTTTCTCCACATTTACAAATCTATAAAGTACAAGTTACTAGTTTTTTTCTCTATTATG
EF121347    ------------------------------------------------------------------------------------------------------------------------

wCfe        CACAGGTTAACTGGTACCTTGCTGTTTCTTTTATTAATTGTATTTTTCTTGGTATTTCATATTACATGTTTATTTTCCTAGACTATTTATAGTGAAATACTTAA
EF121347    --------------------------------------------------------------------------------------------------------
```

Supplementary fig. S2. Generation of orthologous groups (OGs) of proteins across three *R. felis* genomes.

(A) FastOrthoMCL, an in-house modified version of OrthoMCL (Li, et al. 2003), was used to generate OGs from a total of 4,835 predicted proteins from the genomes of *R. felis* str. URRWXCal2, *R. felis* str. LSU and *R. felis* str. LSU-Lb (summarized in gray box at top left). Plasmid proteins were not included. Unique proteins (singletons) not grouped into OGs are distinguished by the dashed arrow, with statistics shown in the yellow box (top right). Characteristics of different OG categories are described moving throughout the flowchart (following the gray arrows). OGs unique to *R. felis* strains LSU and LSU-Lb (green box) were manually evaluated, with results shown in panel B. Several OGs unique to strain LSU or LSU-Lb (yellow box) were combined with singletons and manually evaluated, with results shown in panel C. Numbers shown on the Venn diagram in **fig. 1*d*** are the result of these manual evaluations. Total numbers of proteins per genome that were grouped into OGs are shown in the gray box at bottom left.

(B) Manual evaluation of singletons. Results for *R. felis* strains LSU and LSU-Lb are shown at left and right, respectively. All singletons were used as queries in BLASTP analyses against the NR database. Proteins were then separated into 'top hits to *R. felis* str. URRWXCal2' (top), 'top hits to other *Rickettsia* spp.' (middle), and 'top hits to other bacteria' (bottom: QL, query length; SL, subject length). A summary for the final singleton calculations for each genome is provided. Briefly, proteins with top hits to *R. felis* were determined to be fragments of larger proteins included in OGs with *R. felis* str. URRWXCal2, and were thus culled from the *R. felis* str. LSU and *R. felis* str. LSU-Lb accessory genomes (numbers adjusted in **fig. 1*d***). Proteins having no significant matches in BLASTP searches, *R. felis* str. LSU (n = 19) and *R. felis* str. LSU-lb (n = 12) were also excluded from final tallies.

(C) Manual evaluation of 141 OGs containing proteins only from *R. felis* str. LSU and *R. felis* str. LSU-lb. All proteins were used as queries in BLASTP analyses against the NR database. Proteins were then separated into 'top hits to *R. felis* str. URRWXCal2' (top), 'top hits to other *Rickettsia* spp.' (middle), and 'top hits to other bacteria' (bottom: QL, query length; SL, subject length). A summary for the final calculation is provided. Briefly, proteins with top hits to *R. felis* were determined to be fragments of larger proteins included in OGs with *R. felis* str. URRWXCal2, and were thus culled from the portion of the accessory genome shared by both *R.*

felis str. LSU and *R. felis* str. LSU-Lb (number adjusted in **fig. 1*d***). OGs with proteins having no significant matches in BLASTP searches (*n* = 28) were also excluded from final tallies.

Literature Cited

Li L, Stoeckert CJ, Jr., Roos DS 2003. OrthoMCL: identification of ortholog groups for eukaryotic genomes. Genome research 13: 2178-2189. doi: 10.1101/gr.1224503

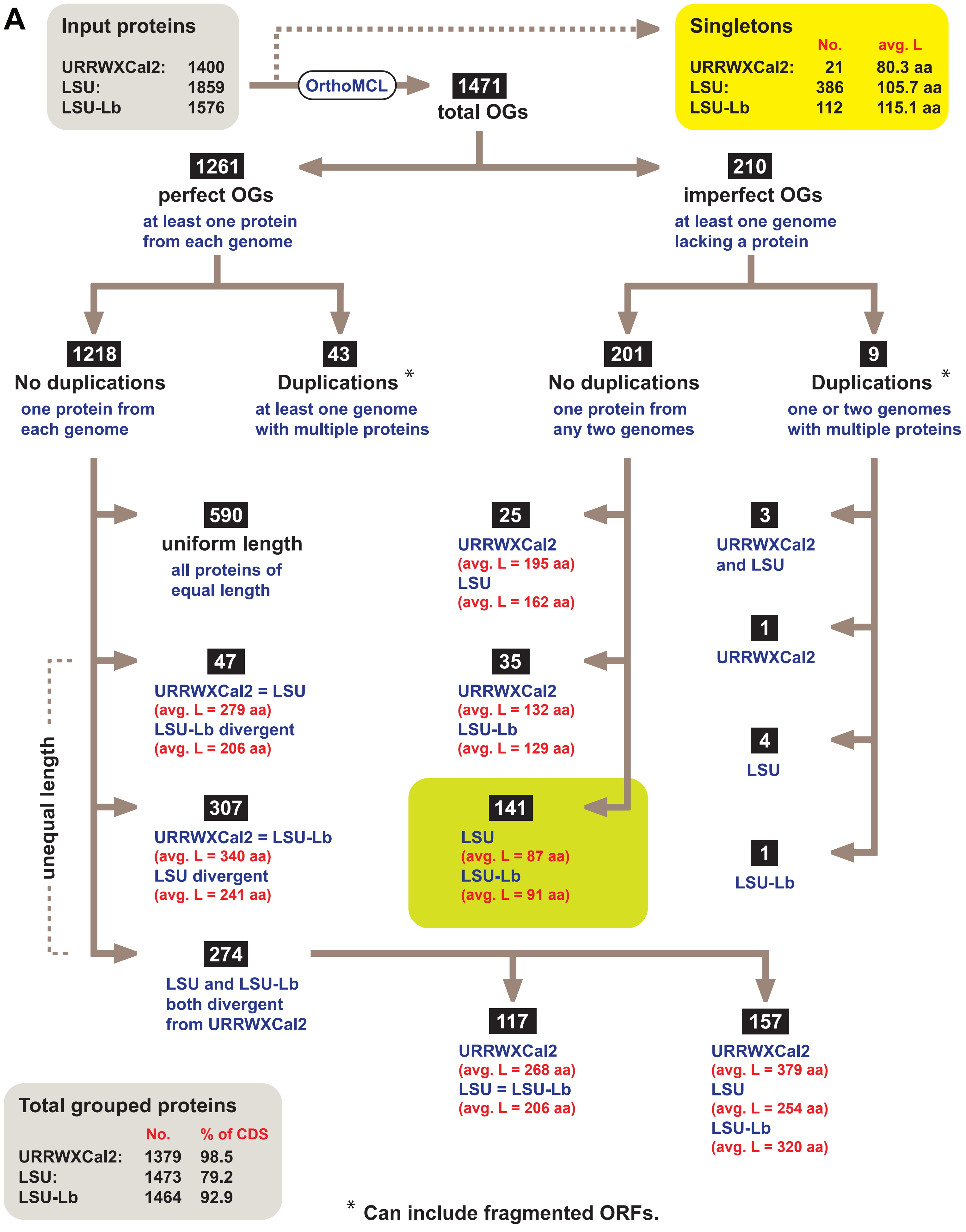
A
Input proteins
URRWXCal2: 1400
LSU: 1859
LSU-Lb 1576
OrthoMCL
1471
total OGs
Singletons
No. avg. L
URRWXCal2: 21 80.3 aa
LSU: 386 105.7 aa
LSU-Lb 112 115.1 aa
1261
perfect OGs
at least one protein from each genome
210
imperfect OGs
at least one genome lacking a protein
1218
No duplications
one protein from each genome
43
Duplications *
at least one genome with multiple proteins
201
No duplications
one protein from any two genomes
9
Duplications *
one or two genomes with multiple proteins
590
uniform length
all proteins of equal length
47
URRWXCal2 = LSU
(avg. L = 279 aa)
LSU-Lb divergent
(avg. L = 206 aa)
307
URRWXCal2 = LSU-Lb
(avg. L = 340 aa)
LSU divergent
(avg. L = 241 aa)
274
LSU and LSU-Lb both divergent from URRWXCal2
unequal length
25
URRWXCal2
(avg. L = 195 aa)
LSU
(avg. L = 162 aa)
35
URRWXCal2
(avg. L = 132 aa)
LSU-Lb
(avg. L = 129 aa)
141
LSU
(avg. L = 87 aa)
LSU-Lb
(avg. L = 91 aa)
3
URRWXCal2 and LSU
1
URRWXCal2
4
LSU
1
LSU-Lb
117
URRWXCal2
(avg. L = 268 aa)
LSU = LSU-Lb
(avg. L = 206 aa)
157
URRWXCal2
(avg. L = 379 aa)
LSU
(avg. L = 254 aa)
LSU-Lb
(avg. L = 320 aa)
Total grouped proteins
No. % of CDS
URRWXCal2: 1379 98.5
LSU: 1473 79.2
LSU-Lb 1464 92.9
* Can include fragmented ORFs.

B

Top hits to *R. felis* str. URRWXCal2

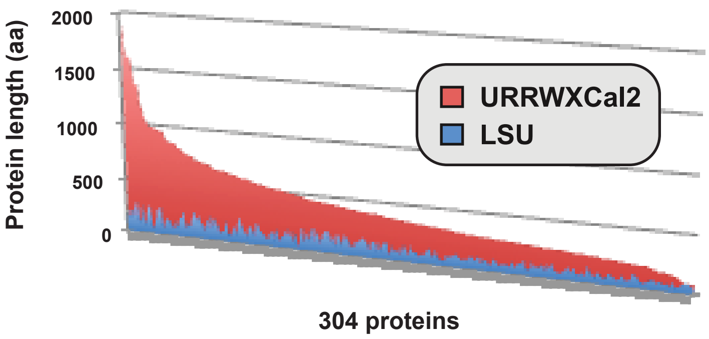

Top hits to other *Rickettsia* spp.

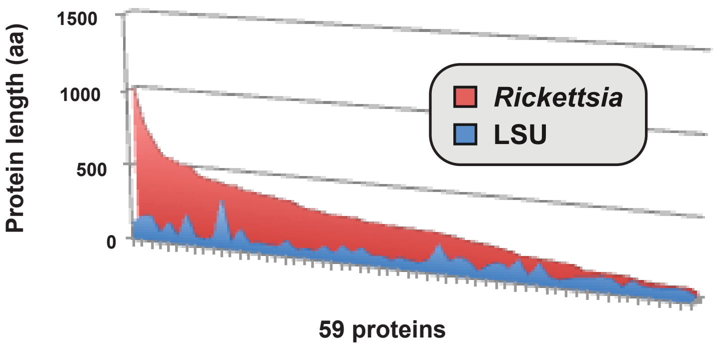

Top hits to other bacteria

	QL	SL
Hypothetical protein: *Mycoplasma hyopneumoniae*	103	177
Hypothetical protein: Rickettsiaceae bacterium Os18	100	171
Hypothetical protein: Rickettsiaceae bacterium Os18	125	147
Hypothetical protein: Rickettsiaceae bacterium Os18	55	67

Summary

304 protein fragments excluded
19 proteins lacking BLASTP matches removed
63 remaining *R. felis* str. LSU singletons

Top hits to *R. felis* str. URRWXCal2

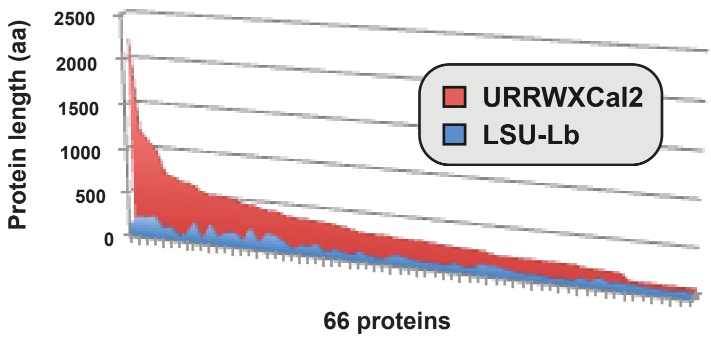

Top hits to other *Rickettsia* spp.

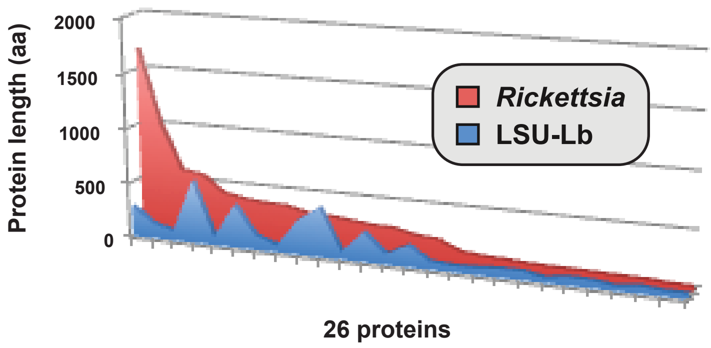

Top hits to other bacteria

	QL	SL
Transposase: *Wolbachia* (*Lissorhoptrus oryzophilus*)	59	443
Transposase: *Burkholderia* sp. CCGE1002	298	341
Transposase: *Waddlia chondrophila* 2032/99	92	335
Transposase: *Wolbachia* (*Culex quinquefasciatus*)	222	280
Transposase: *Wolbachia* sp. wTai	144	144
Transposase: *Legionella drancourtii*	74	130
Hypothetical protein: *Bacillus* sp. 2_A_57_CT2	46	125
Hypothetical protein: *Desulfovibrio bastinii*	89	96

Summary

66 protein fragments excluded
12 proteins lacking BLASTP matches removed
34 remaining *R. felis* str. LSU-Lb singletons

C

Top hits to *R. felis* str. URRWXCal2

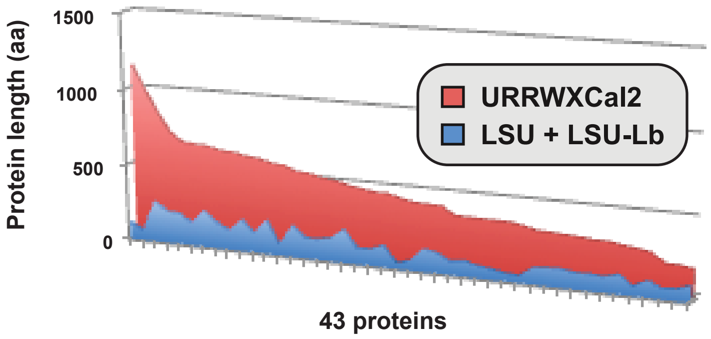

Top hits to other *Rickettsia* spp.

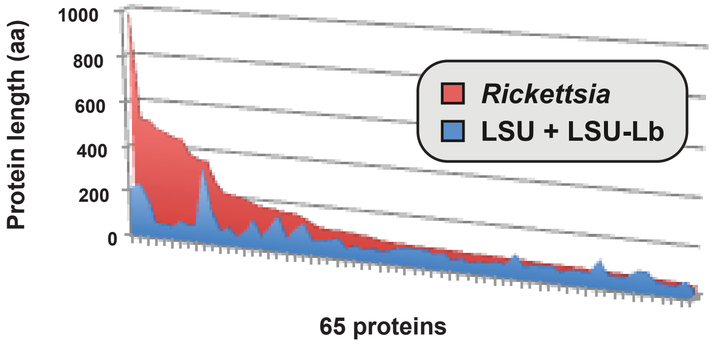

Top hits to other bacteria

	QL	SL
Conserved hypothetical protein: *Magnetospirillum gryphiswaldense* MSR-1	195	183
Hypothetical protein: *Holospora undulata*	49	127
Hypothetical protein: *Corynebacterium pseudogenitalium*	74	74
Putative uncharacterized protein: *Azospirillum* sp. CAG:239	62	64
Putative uncharacterized protein YcdV: *Escherichia coli*	60	63

Summary

43 OGs (protein fragments) excluded
28 proteins lacking BLASTP matches removed
48 remaining OGs shared by *R. felis* str. LSU and *R. felis* str. LSU-Lb

Supplementary fig. S3. Synteny analysis across three *R. felis* genomes (chromosomes).

Genome alignment was performed with the complete chromosome sequence of *Rickettsia felis* str. URRWXCal2 (NC_007109), 21 stitched contigs from the *R. felis* str. LSU assembly, and 35 stitched contigs from the *R. felis* str. LSU-Lb assembly. Genome sequence alignments were performed using Mauve v.2.3.1, utilizing the “progressiveMauve” option (Darling, et al. 2010).

Literature Cited

Darling AE, Mau B, Perna NT 2010. progressiveMauve: multiple genome alignment with gene gain, loss and rearrangement. PLos One 5: e11147. doi: 10.1371/journal.pone.0011147

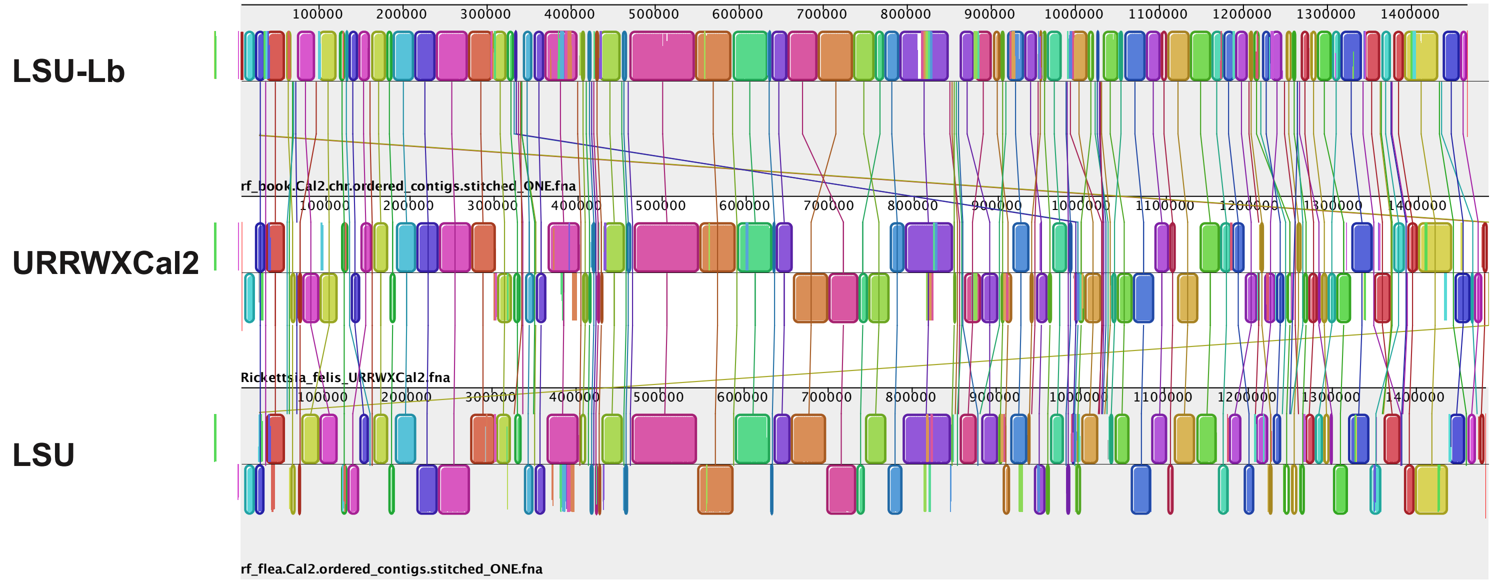

LSU-Lb
URRWXCal2
LSU
rf_book.Cal2.chr.ordered_contigs.stitched_ONE.fna
Rickettsia_felis_URRWXCal2.fna
rf_flea.Cal2.ordered_contigs.stitched_ONE.fna
100000
200000
300000
400000
500000
600000
700000
800000
900000
1000000
1100000
1200000
1300000
1400000

Supplementary fig. S4. Comparison of 16S rDNA, *gltA*, *sca0*, *sca4*, and *sca5* sequences across selected *R. felis* strains.

Aside from sequences derived from the genome assemblies, accession numbers for other *R. felis* strains (all associated with booklice) are provided. For the *R. felis* str. LSU 16S rDNA sequence, red indicates a portion of the sequence that was misassembled. Highlighted positions indicate variable characters shown in **fig. 1*e*** (NOTE: no variation was observed across *sca4* sequences, thus *sca4* was excluded from **fig. 1*e***). Alignments were performed using MUSCLE v3.6 (default parameters) (Edgar 2004). Asterisks denote positions conserved across all input sequences.

16S rDNA sequences

AZ-Lb, *Rickettsia felis* clone Ar1 (GQ329872); AZ-Lb2, *Rickettsia felis* clone Te1 (GQ329874); AUS-Lb, *Rickettsia felis* clone As1 (GQ329876).

```
LSU             ---------------------------------GAAAATGAAGAAGAAAAAGAGGCTTAT
Cal2            GACAGAATCAAACTTGAGAGTTTGATCCTGGCTCAGAACGAACGCTATCGGTATGCTTAA
LSU-Lb          -------TCAAACTTGAGAGTTTGATCCTGGCTCAGAACGAACGCTATCGGTATGCTTAA
AZ-Lb           ------------------------------------------------------------
AZ-Lb2          ------------------------------------------------------------
AUS-Lb          ------------------------------------------------------------

LSU             AACTGG--AGTAAGAGTAATGAATTAAAGCAAAAAGAAATAGAAGATGCGTATAAGAAAA
Cal2            CACATGCAAGTCGGACGGACTAATTGGGGCTTGCTCCAATTAGTTAGTGGCAGACGGGTG
LSU-Lb          CACATGCAAGTCGGACGGACTAATTAGGGCTTGCTCCAATTAGTTAGTGGCAGACGGGTG
AZ-Lb           ------------------------------------------------------------
AZ-Lb2          ------------------------------------------------------------
AUS-Lb          ------------------------------------------------------------

LSU             AACAAGATGAGGAAAGTATAGACAATTATAAACAATTAAGTGTAGAGGTGCAAGCGTCAA
Cal2            AGTAACACGTGGGAA-TCTGCCCATCAGTACGGAATAACTTTTAGAAATAAAAGC-----
LSU-Lb          AGTAACACGTGGGAA-TCTGCCCATCAGTACGGAATAACTTTTAGAAATAAAAGC-----
AZ-Lb           ----ACACGTGGGAA-TCTGCCCATCAGTACGGAATAACTTTTAGAAATAAAAGC-----
AZ-Lb2          --TAACACGTGGGAA-TCTGCCCATCAGTACGGAATAACTTTTAGAAATAAAAGC-----
AUS-Lb          -GTAACACGTGGGAA-TCTGCCCATCAGTACGGAATAACTTTTAGAAATAAAAGC-----
                    * * * ** ** * *   **    **   *** *  * ****  *  ****

LSU             GGCCTAAAGCATCATCCGATGTTAGCAACGGAAGTAAAATGATACAGAGCAACGGATGAA
Cal2            ---------TAATACCGTATATTCTCTACAGAGGAAAGAT---------TTATCGCTGAT
LSU-Lb          ---------TAATACCGTATATTCTCTACAGAGGAAAGAT---------TTATCGCTGAT
AZ-Lb           ---------TAATACCGTATATTCTCTACAGAGGAAAGAT---------TTATCGCTGAT
AZ-Lb2          ---------TAATACCGTATATTCTCTACAGAGGAAAGAT---------TTATCGCTGAT
AUS-Lb          ---------TAATACCGTATATTCTCTACAGAGGAAAGAT---------TTATCGCTGAT
                          *  * *  ** **  * ** ** * ** **           *  * ***

LSU             AATTGATACAGTATTAAAGTAATATCTTCTGGATAAGGAGGTAATGAATAA------TGA
Cal2            GGATGAGCCCGCGTCAGATTAGG------TAGTTGGTGAGGTAACGGCTCACCAAGCCGA
LSU-Lb          GGATGAGCCCGCGTCAGATTAGG------TAGTTGGTGAGGTAACGGCTCACCAAGCCGA
AZ-Lb           GTATGAGCCCGCGTCAGATTAGK------TAGTTGGTGAGGTAACGGCTCACCAAGCCGA
AZ-Lb2          GTATGAGCCCGCGTCAGATTAGK------TAGTTGGTGAGGTAACGGCTCACCAAGCCGA
AUS-Lb          GTATGAGCCCGCGTCAGATTAGK------TAGTTGGTGAGGTAACGGCTCACCAAGCCGA
                   ***  * *  * * * **        * * *   ******* *  * *       **

LSU             TAATACTGGAGCAAAT---AATGGAAATAAAAATATTGAATAAACATGGTAAGAGCCTAA
Cal2            CGAT-CTGTAGCTGGTCTGAGAGGA-----------TGATCAGCCACACTGGGA--CTGA
LSU-Lb          CGAT-CTGTAGCTGGTCTGAGAGGA-----------TGATCAGCCACACTGGGA--CTGA
AZ-Lb           CGAT-CTGTAGCTGGTCTGAGGGGA-----------TGATCAGCCACACTGGGA--CTGA
AZ-Lb2          CGAT-CTGTAGCTGGTCTGAGGGGA-----------TGATCAGCCACACTGGGA--CTGA
AUS-Lb          CGAT-CTGTAGCTGGTCTGAGGGGA-----------TGATCAGCCACACTGGGA--CTGA
                  ** *** ***   *   *  ***           ***  *  **   *  **  ** *

LSU             GA--------AGTATAGCAAGAGAAGTAGGAGTATCAGTAAATACAGTACGTAAATATTT
Cal2            GACACGGCCCAGACTCCTACGGGAGGCAGCAGTG----------------GGGAATATTG
LSU-Lb          GACACGGCCCAGACTCCTACGGGAGGCAGCAGTG----------------GGGAATATTG
AZ-Lb           GACACGGCCCAGACTCCTACGGGAGGCAGCAGTG----------------GGGAATATTG
AZ-Lb2          GACACGGCCCAGACTCCTACGGGAGGCAGCAGTG----------------GGGAATATTG
AUS-Lb          GACACGGCCCAGACTCCTACGGGAGGCAGCAGTG----------------GGGAATATTG
                **        **  *   * * ** * ** ***                 *  ******

LSU             AAAAATATGATGGTAGCCCTAAATATAAGGATAGGTCGGAGTTGGTAACAAAGCTTGCCC
Cal2            GACAATG-GGCGAAAGCCT--GATCCAGCAATA---CCGAGTGAGTGATGAAG---GCCC
LSU-Lb          GACAATG-GGCGAAAGCCT--GATCCAGCAATA---CCGAGTGAGTGATAAAG---GCCC
AZ-Lb           GACAATG-GGCGAAAGCCT--GATCCAGCAATA---CCGAGTGAGTGATGAAG---GCCC
AZ-Lb2          GACAATG-GGCGAAAGCCT--GATCCAGCAATA---CCGAGTGAGTGATGAAG---GCCC
AUS-Lb          GACAATG-GGCGAAAGCCT--GATCCAGCAATA---CCGAGTGAGTGATGAAG---GCCC
                 * ***  *  *  ****    **  *   ***   * ****  ** *  ***   ****
```

```
LSU             ---------------------------------------------------------CCC
Cal2            TAGGGTTGTAAAG-CTCTTTTAGC-AAGGAAGATAATGACGTTACTTGCAGAAAAAGCCC
LSU-Lb          TAGGGTTGTAAAG-CTCTTTTAGC-AAGGAAGATAATGACGTTACTTGCAGAAAAAGCCC
AZ-Lb           TAGGGTTGTAAAG-CTCTTTTAGC-AAGGAAGATAATGACGTTACTTGCAGAAAAAGCCC
AZ-Lb2          TAGGGTTGTAAAG-CTCTTTTAGC-AAGGAAGATAATGACGTTACTTGCAGAAAAAGCCC
AUS-Lb          TAGGGTTGTAAAG-CTCTTTTAGC-AAGGAAGATAATGACGTTACTTGCAGAAAAAGCCC
                 *    * ***** **  ** **  ** *** * *** *   *              ***

LSU             CGGCTAACTCCGTGCCAGCAGCCGCGGTAAGACGGAGGGGGCTAGCGTTGTTCGGAATTA
Cal2            CGGCTAACTCCGTGCCAGCAGCCGCGGTAAGACGGAGGGGGCTAGCGTTGTTCGGAATTA
LSU-Lb          CGGCTAACTCCGTGCCAGCAGCCGCGGTAAGACGGAGGGGGCTAGCGTTGTTCGGAATTA
AZ-Lb           CGGCTAACTCCGTGCCAGCAGCCGCGGTAAGACGGAGGGGGCTAGCGTTGTTCGGAATTA
AZ-Lb2          CGGCTAACTCCGTGCCAGCAGCCGCGGTAAGACGGAGGGGGCTAGCGTTGTTCGGAATTA
AUS-Lb          CGGCTAACTCCGTGCCAGCAGCCGCGGTAAGACGGAGGGGGCTAGCGTTGTTCGGAATTA
                ************************************************************

LSU             CTGGGCGTAAAGAGTGCGTAGGCGGTTTAGTAAGTTGGAAGTGAAAGCCCGGGGCTTAAC
Cal2            CTGGGCGTAAAGAGTGCGTAGGCGGTTTAGTAAGTTGGAAGTGAAAGCCCGGGGCTTAAC
LSU-Lb          CTGGGCGTAAAGAGTGCGTAGGCGGTTTAGTAAGTTGGAAGTGAAAGCCCGGGGCTTAAC
AZ-Lb           CTGGGCGTAAAGAGTGCGTAGGCGGTTTAGTAAGTTGGAAGTGAAAGCCCGGGGCTTAAC
AZ-Lb2          CTGGGCGTAAAGAGTGCGTAGGCGGTTTAGTAAGTTGGAAGTGAAAGCCCGGGGCTTAAC
AUS-Lb          CTGGGCGTAAAGAGTGCGTAGGCGGTTTAGTAAGTTGGAAGTGAAAGCCCGGGGCTTAAC
                ************************************************************

LSU             CTCGGAATTGCTTTCAAAACTACTAATCTAGAGTGTAGTAGGGGATGATGGAATTCCTAG
Cal2            CTCGGAATTGCTTTCAAAACTACTAATCTAGAGTGTAGTAGGGGATGATGGAATTCCTAG
LSU-Lb          CTCGGAATTGCTTTCAAAACTACTAATCTAGAGTGTAGTAGGGGATGATGGAATTCCTAG
AZ-Lb           CTCGGAATTGCTTTCAAAACTACTAATCTAGAGTGTAGTAGGGGATGATGGAATTCCTAG
AZ-Lb2          CTCGGAATTGCTTTCAAAACTACTAATCTAGAGTGTAGTAGGGGATGATGGAATTCCTAG
AUS-Lb          CTCGGAATTGCTTTCAAAACTACTAATCTAGAGTGTAGTAGGGGATGATGGAATTCCTAG
                ************************************************************

LSU             TGTAGAGGTGAAATTCTTAGATATTAGGAGGAACACCGGTGGCGAAGGCGGTCATCTGGG
Cal2            TGTAGAGGTGAAATTCTTAGATATTAGGAGGAACACCGGTGGCGAAGGCGGTCATCTGGG
LSU-Lb          TGTAGAGGTGAAATTCTTAGATATTAGGAGGAACACCGGTGGCGAAGGCGGTCATCTGGG
AZ-Lb           TGTAGAGGTGAAATTCTTAGATATTAGGGGGAACACCGGTGGCGAAGGCGGTCATCTGGG
AZ-Lb2          TGTAGAGGTGAAATTCTTAGATATTAGGGGGAACACCGGTGGCGAAGGCGGTCATCTGGG
AUS-Lb          TGTAGAGGTGAAATTCTTAGATATTAGGGGGAACACCGGTGGCGAAGGCGGTCATCTGGG
                **************************** *******************************

LSU             CTACAACTGACGCTGATGCACGAAAGCGTGGGGAGCAAACAGGATTAGATACCCTGGTAG
Cal2            CTACAACTGACGCTGATGCACGAAAGCGTGGGGAGCAAACAGGATTAGATACCCTGGTAG
LSU-Lb          CTACAACTGACGCTGATGCACGAAAGCGTGGGGAGCAAACAGGATTAGATACCCTGGTAG
AZ-Lb           CTACAACTGACGCTGATGCACGAAAGCGTGGGGAGCAAACAGGATTAGATACCCTGGTAG
AZ-Lb2          CTACAACTGACGCTGATGCACGAAAGCGTGGGGAGCAAACAGGATTAGATACCCTGGTAG
AUS-Lb          CTACAACTGACGCTGATGCACGAAAGCGTGGGGAGCAAACAGGATTAGATACCCTGGTAG
                ************************************************************

LSU             TCCACGCCGTAAACGATGAGTGCTAGATATCGGAAGATTCTCTTTCGGTTTCGCAGCTAA
Cal2            TCCACGCCGTAAACGATGAGTGCTAGATATCGGAAGATTCTCTTTCGGTTTCGCAGCTAA
LSU-Lb          TCCACGCCGTAAACGATGAGTGCTAGATATCGGAAGATTCTCTTTCGGTTTCGCAGCTAA
AZ-Lb           TCCACGCCGTAAACGATGAGTGCTAGATATCGGAAGATTCTCTTTCGGTTTCGCAGCTAA
AZ-Lb2          TCCACGCCGTAAACGATGAGTGCTAGATATCGGAAGATTCTCTTTCGGTTTCGCAGCTAA
AUS-Lb          TCCACGCCGTAAACGATGAGTGCTAGATATCGGAAGATTCTCTTTCGGTTTCGCAGCTAA
                ************************************************************

LSU             CGCATTA-AGCACTCCGCCTGGGGAGTACGGTCGCAAGATTAAAACTCAAAGGAATTGAC
Cal2            CGCATTA-AGCACTCCGCCTGGGGAGTACGGTCGCAAGATTAAAACTCAAAGGAATTGAC
LSU-Lb          CGCATTA-AGCACTCCGCCTGGGGAGTACGGTCGCAAGATTAAAACTCAAAGGAATTGAC
AZ-Lb           CGCATTATAGCACTCCGCCTGGGGAGTACGGTCGCAAGATTAAAACTCAAAGGAATTGAC
AZ-Lb2          CGCATTATAGCACTCCGCCTGGGGAGTACGGTCGCAAGATTAAAACTCAAAGGAATTGAC
AUS-Lb          CGCATTATAGCACTCCGCCTGGGGAGTACGGTCGCAAGATTAAAACTCAAAGGAATTGAC
                ******* ****************************************************

LSU             GGGGGCTCGCACAAGCGGTGGAGCATGCGGTTTAATTCGATGTTACGCGAAAAACCTTAC
AZ-Lb           GGGGGCTCGCACAAGCGGTGGAGCATGCGGTTTAATTCGATGTTACGCGAAAAACCTTAC
AZ-Lb2          GGGGGCTCGCACAAGCGGTGGAGCATGCGGTTTAATTCGATGTTACGCGAAAAACCTTAC
AUS-Lb          GGGGGCTCGCACAAGCGGTGGAGCATGCGGTTTAATTCGATGTTACGCGAAAAACCTTAC
Cal2            GGGGGCTCGCACAAGCGGTGGAGCATGCGGTTTAATTCGATGTTACGCGAAAAACCTTAC
LSU-Lb          GGGGGCTCGCACAAGCGGTGGAGCATGCGGTTTAATTCGATGTTACGCGAAAAACCTTAC
                ************************************************************
```

```
LSU             CAACCCTTGACATGGTGGTCGCGGATCGCAGAGATGCTTTCCTTCAGCTCGGCTGGACCA
Cal2            CAACCCTTGACATGGTGGTCGCGGATCGCAGAGATGCTTTCCTTCAGCTCGGCTGGACCA
LSU-Lb          CAACCCTTGACATGGTGGTCGCGGATCGCAGAGATGCTTTCCTTCAGCTCGGCTGGACCA
AZ-Lb           CAACCCTTGACATGGTGGTCGCGGATCGCAGAGATGCTTTCCTTCAGCTCGGCTGGACCA
AZ-Lb2          CAACCCTTGACATGGTGGTCGCGGATCGCAGAGATGCTTTCCTTCAGCTCGGCTGGACCA
AUS-Lb          CAACCCTTGACATGGTGGTCGCGGATCGCAGAGATGCTTTCCTTCAGCTCGGCTGGACCA
                ************************************************************

LSU             CACACAGGTGTTGCATGGCTGTCGTCAGCTCGTGTCGTGAGATGTTGGGTTAAGTCCCGC
Cal2            CACACAGGTGTTGCATGGCTGTCGTCAGCTCGTGTCGTGAGATGTTGGGTTAAGTCCCGC
LSU-Lb          CACACAGGTGTTGCATGGCTGTCGTCAGCTCGTGTCGTGAGATGTTGGGTTAAGTCCCGC
AZ-Lb           CACACAGGTGTTGCATGGCTGTCGTCAGCTCGTGTCGTGAGATGTTGGGTTAAGTCCCGC
AZ-Lb2          CACACAGGTGTTGCATGGCTGTCGTCAGCTCGTGTCGTGAGATGTTGGGTTAAGTCCCGC
AUS-Lb          CACACAGGTGTTGCATGGCTGTCGTCAGCTCGTGTCGTGAGATGTTGGGTTAAGTCCCGC
                ************************************************************

LSU             AACGAGCGCAACCCTCATTCTTATTTGCCAGCGGGTAATGCCGGGAACTATAAGAAAACT
Cal2            AACGAGCGCAACCCTCATTCTTATTTGCCAGCGGGTAATGCCGGGAACTATAAGAAAACT
LSU-Lb          AACGAGCGCAACCCTCATTCTTATTTGCCAGCGGGTAATGCCGGGAACTATAAGAAAACT
AZ-Lb           AACGAGCGCAACCCTCATTCTTATTTGCCAGCGGGTAATGCCGGGAACTATAAGAAAACT
AZ-Lb2          AACGAGCGCAACCCTCATTCTTATTTGCCAGCGGGTAATGCCGGGAACTATAAGAAAACT
AUS-Lb          AACGAGCGCAACCCTCATTCTTATTTGCCAGCGGGTAATGCCGGGAACTATAAGAAAACT
                ************************************************************

LSU             GCCGGTGATAAGCCGGAGGAAGGTGGGGACGACGTCAAGTCATCATGGCCCTTACGGGTT
Cal2            GCCGGTGATAAGCCGGAGGAAGGTGGGGACGACGTCAAGTCATCATGGCCCTTACGGGTT
LSU-Lb          GCCGGTGATAAGCCGGAGGAAGGTGGGGACGACGTCAAGTCATCATGGCCCTTACGGGTT
AZ-Lb           GCCGGTGATAAGCCGGAGGAAGGTGGGGACGACGTCAAGTCATCATGGCCCTTACGGGTT
AZ-Lb2          GCCGGTGATAAGCCGGAGGAAGGTGGGGACGACGTCAAGTCATCATGGCCCTTACGGGTT
AUS-Lb          GCCGGTGATAAGCCGGAGGAAGGTGGGGACGACGTCAAGTCATCATGGCCCTTACGGGTT
                ************************************************************

LSU             GGGCTACACGCGTGCTACAATGGTGTTTACAGAGGGAAGCAAGACGGCGACGTGGAGCAA
Cal2            GGGCTACACGCGTGCTACAATGGTGTTTACAGAGGGAAGCAAGACGGCGACGTGGAGCAA
LSU-Lb          GGGCTACACGCGTGCTACAATGGTGTTTACAGAGGGAAGCAAGACGGCGACGTGGAGCAA
AZ-Lb           GGGCTACACGCGTGCTACAATGGTGTTTACAGAGGGAAGCAAGACGGCGACGTGGAGCAA
AZ-Lb2          GGGCTACACGCGTGCTACAATGGTGTTTACAGAGGGAAGCAAGACGGCGACGTGGAGCAA
AUS-Lb          GGGCTACACGCGTGCTACAATGGTGTTTACAGAGGGAAGCAAGACGGCGACGTGGAGCAA
                ************************************************************

LSU             ATCCCTAAAAGACATCTCAGTTCGGATTGTTCTCTGCAACTCGAGAGCATGAAGTTGGAA
AZ-Lb           ATCCCTAAAAGACATCTCAGTTCGGATTGTTCTCTGCAACTCGAGAGCATGAAGTTGGAA
Cal2            ATCCCTAAAAGACATCTCAGTTCGGATTGTTCTCTGCAACTCGAGAGCATGAAGTTGGAA
LSU-Lb          ATCCCTAAAAGACATCTCAGTTCGGATTGTTCTCTGCAACTCGAGAGCATGAAGTTGGAA
AZ-Lb2          ATCCCTAAAAGACATCTCAGTTCGGATTGTTCTCTGCAACTCGAGAGCATGAAGTTGGAA
AUS-Lb          ATCCCTAAAAGACATCTCAGTTCGGATTGTTCTCTGCAACTCGAGAGCATGAAGTTGGAA
                ************************************************************

LSU             TCGCTAGTAATCGCGGATCAGCATGCCGCGGTGAATACGTTCTCGGGCCTTGTACACACT
Cal2            TCGCTAGTAATCGCGGATCAGCATGCCGCGGTGAATACGTTCTCGGGCCTTGTACACACT
LSU-Lb          TCGCTAGTAATCGCGGATCAGCATGCCGCGGTGAATACGTTCTCGGGCCTTGTACACACT
AZ-Lb           TCGCTAGTAATCGCGGA-------------------------------------------
AZ-Lb2          TCGCTAGTAATCGCGGAT------------------------------------------
AUS-Lb          TCGCTAGTAATCGCGGAT------------------------------------------
                *****************

LSU             GCCCGTCACGCCATGGGAGTTGGTTTTACCTGAAGGTGGTGAGCTAACGCAAGAGGCAGC
Cal2            GCCCGTCACGCCATGGGAGTTGGTTTTACCTGAAGGTGGTGAGCTAACGCAAGAGGCAGC
LSU-Lb          GCCCGTCACGCCATGGGAGTTGGTTTTACCTGAAGGTGGTGAGCTAACGCAAGAGGCAGC
AZ-Lb           ------------------------------------------------------------
AZ-Lb2          ------------------------------------------------------------
AUS-Lb          ------------------------------------------------------------


LSU             CAACCACGGTAAAATTAGCGACTGGGGTGAAGTCGTAACAAGGTAGCCGTAGGGGAACCT
Cal2            CAACCACGGTAAAATTAGCGACTGGGGTGAAGTCGTAACAAGGTAGCCGTAGGGGAACCT
LSU-Lb          CAACCACGGTAAAATTAGCGACTGGGGTGAAGTCGTAACAAGGTAGCCGTAGGGGAACCT
AZ-Lb           ------------------------------------------------------------
AZ-Lb2          ------------------------------------------------------------
AUS-Lb          ------------------------------------------------------------
```

```
LSU             GCGGCTGGATTACCTCCTTA
Cal2            GCGGCTGGATTACCTCCTTA
LSU-Lb          GCGGCTGGATTACCTCCTTA
AZ-Lb           --------------------
AZ-Lb2          --------------------
AUS-Lb          --------------------
```

gltA sequences

Ar2-Lb, *Rickettsia felis* clone Ar2 (GQ329873); As2-Lb, *Rickettsia felis* clone As2 (GQ329877).

```
Cal2            ATGACCAATGAAAATAGTAATGATTCAGAATTTGCCGAATTAAAAATCAGAGGAAAAATA
LSU             ATGACCAATGAAAATAGTAATGATTCAGAATTTGCCGAATTAAAAATCAGAGGAAAAATA
LSU-Lb          ATGACCAATGAAAATAGTAATGATTCAGAATTTGCCGAATTAAAAATCAGAGGAAAAATA
Ar2-Lb          ------------------------------------------------------------
As2-Lb          ------------------------------------------------------------

Cal2            TTTAAATTACCTATACTTAAAGCAAGTATTGGTGAGGATGTAATCGATATAAGCAGGGTA
LSU             TTTAAATTACCTATACTTAAAGCAAGTATTGGTGAGGATGTAATCGATATAAGCAGGGTA
LSU-Lb          TTTAAATTACCTATACTTAAAGCAAGTATTGGTGAGGATGTAATCGATATAAGCAGGGTA
Ar2-Lb          ------------------------------------------------------------
As2-Lb          ------------------------------------------------------------

Cal2            TCTTCGGAAGCCGATTGTTTTACTTATGACCCGGGCTTTATGTCTACTGCTTCTTGTCAG
LSU             TCTTCGGAAGCCGATTGTTTTACTTATGACCCGGGCTTTATGTCTACTGCTTCTTGTCAG
LSU-Lb          TCTTCGGAAGCCGATTGTTTTACTTATGACCCGGGCTTTATGTCTACTGCTTCTTGTCAG
Ar2-Lb          ------------------------------------------------------------
As2-Lb          ------------------------------------------------------------

Cal2            TCTACTATCACTTATATAGACGGTGATAAAGGAATCTTGCGACATCGAGGATATGACATT
LSU             TCTACTATCACTTATATAGACGGTGATAAAGGAATCTTGCGACATCGAGGATATGACATT
LSU-Lb          TCTACTATCACTTATATAGACGGTGATAAAGGAATCTTGCGACATCGAGGATATGACATT
Ar2-Lb          ------------------------------------------------------------
As2-Lb          ------------------------------------------------------------

Cal2            AAAGATTTAGCCGAGAAAAGTGATTTTTTAGAAGTGGCATATTTGCTAATCTACGGAGAA
LSU             AAAGATTTAGCCGAGAAAAGTGATTTTTTAGAAGTGGCATATTTGCTAATCTACGGAGAA
LSU-Lb          AAAGATTTAGCCGAGAAAAGTGATTTTTTAGAAGTGGCATATTTGCTAATCTACGGAGAA
Ar2-Lb          ------------------------------------------------------------
As2-Lb          ------------------------------------------------------------

Cal2            TTGCCAAGCATCGAGCAGTATAATAATTTCACTAAACAGGTTGCTCATCATTCATTAGTG
LSU             TTGCCAAGCATCGAGCAGTATAATAATTTCACTAAACAGGTTGCTCATCATTCATTAGTG
LSU-Lb          TTGCCAAGCATCGAGCAGTATAATAATTTCACTAAACAGGTTGCTCATCATTCATTAGTG
Ar2-Lb          ------------------------------------------------------------
As2-Lb          ------------------------------------------------------------

Cal2            AATGAAAGATTACACTATTTATTTCAAACATTTTGTAGCTCTTCTCATCCTATGGCTATT
LSU             AATGAAAGATTACACTATTTATTTCAAACATTTTGTAGCTCTTCTCATCCTATGGCTATT
LSU-Lb          AATGAAAGATTACACTATTTATTTCAAACATTTTGTAGCTCTTCTCATCCTATGGCTATT
Ar2-Lb          ------------------------------------------------------------
As2-Lb          ------------------------------------------------------------

Cal2            ATGCTTGCGGCCGTTGGTTCTCTTTCGGCATTTTATCCTGATTTACTGAATTTTAAAGAA
LSU             ATGCTTGCGGCCGTTGGTTCTCTTTCGGCATTTTATCCTGATTTACTGAATTTTAAAGAA
LSU-Lb          ATGCTTGCGGCCGTTGGTTCTCTTTCGGCATTTTATCCTGATTTACTGAATTTTAAAGAA
Ar2-Lb          ------------------------------------------------------------
As2-Lb          ------------------------------------------------------------
```

```
Cal2          GCAGACTACGAACTTACCGCTATTAGAATGATTGCTAAGATACCAACTATCGCCGCAATG
LSU           GCAGACTACGAACTTACCGCTATTAGAATGATTGCTAAGATACCAACTATCGCCGCAATG
LSU-Lb        GCAGACTACGAACTTACCGCTATTAGAATGATTGCTAAGATACCAACTATCGCCGCAATG
Ar2-Lb        ------------------------------------------------------------
As2-Lb        ------------------------------------------------------------

Cal2          TCCTATAAATATTCTATAGGACAACCGTTTATTTATCCTGATAATTCGTTAGATTTTACT
LSU           TCCTATAAATATTCTATAGGACAACCGTTTATTTATCCTGATAATTCGTTAGATTTTACT
LSU-Lb        TCCTATAAATATTCTATAGGACAACCGTTTATTTATCCTGATAATTCGTTAGATTTTACT
Ar2-Lb        ------------------------------------------------------------
As2-Lb        ------------------------------------------------------------

Cal2          GAAAATTTTCTGCATATGATGTTTGCAACGCCTTGTACAAAATATAAAGTAAATCCAATA
LSU           GAAAATTTTCTGCATATGATGTTTGCAACGCCTTGTACAAAATATAAAGTAAATCCAATA
LSU-Lb        GAAAATTTTCTGCATATGATGTTTGCAACGCCTTGTACAAAATATAAAGTAAATCCAATA
Ar2-Lb        ------------------------------------------------------------
As2-Lb        ------------------------------------------------------------

Cal2          ATAAAAAATGCTCTTAATAAAATATTTATTCTGCATGCTGACCATGAGCAGAACGCTTCT
LSU           ATAAAAAATGCTCTTAATAAAATATTTATTCTGCATGCTGACCATGAGCAGAACGCTTCT
LSU-Lb        ATAAAAAATGCTCTTAATAAAATATTTATTCTGCATGCTGACCATGAGCAGAACGCTTCT
Ar2-Lb        ------------------------------------------------------------
As2-Lb        ------------------------------------------------------------

Cal2          ACTTCAACAGTCCGAATTGCCGGCTCATCCGGGGCTAACCCTTTTGCTTGCGTAAGTACA
LSU           ACTTCAACAGTCCGAATTGCCGGCTCATCCGGGGCTAACCCTTTTGCTTGCGTAAGTACA
LSU-Lb        ACTTCAACAGTCCGAATTGCCGGCTCATCCGGGGCTAACCCTTTTGCTTGCGTAAGTACA
Ar2-Lb        ------------------------------------------------------------
As2-Lb        ------------------------------------------------------------

Cal2          GGGATTGCCTCACTTTGGGGGCCTGCCCACGGCGGGGCTAATGAAGCGGTAATAAATATG
LSU           GGGATTGCCTCACTTTGGGGGCCTGCCCACGGCGGGGCTAATGAAGCGGTAATAAATATG
LSU-Lb        GGGATTGCCTCACTTTGGGGGCCTGCCCACGGCGGGGCTAATGAAGCGGTAATAAATATG
Ar2-Lb        -----------------GGGACCTGCTCACGGCGGGGCTAATGAAGCGGTAATAAATATG
As2-Lb        -----------------GGGACCTGCTCACGGCGGGGCTAATGAAGCGGTAATAAATATG
                               *** ***** *********************************

Cal2          CTTAAAGAAATCGGTAGTTCTGAGAATATTCCTAAATATATAGCTAAAGCTAAGGATAAA
LSU           CTTAAAGAAATCGGTAGTTCTGAGAATATTCCTAAATATATAGCTAAAGCTAAGGATAAA
LSU-Lb        CTTAAAGAAATCGGTAGTTCTGAGAATATTCCTAAATATATAGCTAAAGCTAAGGATAAA
Ar2-Lb        CTTAAAGAAATCGGTAGTTCTGAGAATATTCCTAAATATATAGCTAAAGCTAAGGATAAA
As2-Lb        CTTAAAGAAATCGGTAGTTCTGAGAATATTCCTAAATATATAGCTAAAGCTAAGGATAAA
              ************************************************************

Cal2          AATGATCCGTTTAGGTTAATGGGTTTCGGTCATCGTGTTTATAAAAACTATGACCCACGT
LSU           AATGATCCGTTTAGGTTAATGGGTTTCGGTCATCGTGTTTATAAAAACTATGACCCACGT
LSU-Lb        AATGATCCGTTTAGGTTAATGGGTTTCGGTCATCGTGTTTATAAAAACTATGACCCACGT
Ar2-Lb        AATGATCCGTTTAGGTTAATGGGTTTCGGTCATCGTGTTTATAAAAACTATGACCCACGT
As2-Lb        AATGATCCGTTTAGGTTAATGGGTTTCGGTCATCGTGTTTATAAAAACTATGACCCACGT
              ************************************************************


Cal2          GCCGCAGTACTTAAAGAAACTTGCAAAGAAGTATTAAAGGAACTCGGACAGCTAGAAAAC
LSU           GCCGCAGTACTTAAAGAAACTTGCAAAGAAGTATTAAAGGAACTCGGACAGCTAGAAAAC
LSU-Lb        GCCGCAGTACTTAAAGAAACTTGCAAAGAAGTATTAAAGGAACTCGGACAGCTAGAAAAC
Ar2-Lb        GCCGCAGTACTTAAAGAAACTTGCAAAGAAGTATTAAAGGAACTCGGACAGCTAGAAAAC
As2-Lb        GCCGCAGTACTTAAAGAAACTTGCAAAGAAGTATTAAAGGAACTCGGACAGCTAGAAAAC
              ************************************************************

Cal2          AATCCGCTTTTGCAAATAGCAATAGAACTTGAAGCTATCGCTCTTAAAGATGAATATTTT
LSU           AATCCGCTTTTGCAAATAGCAATAGAACTTGAAGCTATCGCTCTTAAAGATGAATATTTT
LSU-Lb        AATCCGCTTTTGCAAATAGCAATAGAACTTGAAGCTATCGCTCTTAAAGATGAATATTTT
Ar2-Lb        AATCCGCTTTTGCAAATAGCAATAGAACTTGAAGCTATCGCTCTTAAAGATGAATATTTT
As2-Lb        AATCCGCTTTTGCAAATAGCAATAGAACTTGAAGCTATCGCTCTTAAAGATGAATATTTT
              ************************************************************
```

```
Cal2                ATTGAGAGAAAATTATATCCAAATGTTGATTTTTATTCAGGTATTATCTATAAAGCTATG
LSU                 ATTGAGAGAAAATTATATCCAAATGTTGATTTTTATTCAGGTATTATCTATAAAGCTATG
LSU-Lb              ATTGAGAGAAAATTATATCCAAATGTTGATTTTTATTCAGGTATTATCTATAAAGCTATG
Ar2-Lb              ATTGAGAGAAAATTATATCCAAATGTTGATTTTTATTCAGGTATTATCTATAAAGCTATG
As2-Lb              ATTGAGAGAAAATTATATCCAAATGTTGATTTTTATTCAGGTATTATCTATAAAGCTATG
                    ************************************************************

Cal2                GGTATACCGTCGCAAATGTTTACGGTACTTTTTGCAATAGCAAGAACCGTAGGCTGGATG
LSU                 GGTATACCGTCGCAAATGTTTACGGTACTTTTTGCAATAGCAAGAACCGTAGGCTGGATG
LSU-Lb              GGTATACCGTCGCAAATGTTTACGGTACTTTTTGCAATAGCAAGAACCGTAGGCTGGATG
Ar2-Lb              GGTATACCGTCGCAAATGTTCACTGTACTTTTTGCAATA---------------------
As2-Lb              GGTATACCGTCGCAAATGTTCACTGTACTTTTTGCAATA---------------------
                    ******************** ** ***************

Cal2                GCACAGTGGAAAGAAATGCACGAAGACCCTGAACAAAAAATCAGCAGACCTAGACAGCTT
LSU                 GCACAGTGGAAAGAAATGCACGAAGACCCTGAACAAAAAATCAGCAGACCTAGACAGCTT
LSU-Lb              GCACAGTGGAAAGAAATGCACGAAGACCCTGAACAAAAAATCAGCAGACCTAGACAGCTT
Ar2-Lb              ------------------------------------------------------------
As2-Lb              ------------------------------------------------------------


Cal2                TACACCGGTTATGTACATAGAGAGTATAAGGGTATCGGGGAGAGGTAG
LSU                 TACACCGGTTATGTACATAGAGAGTATAAGGGTATCGGGGAGAGGTAG
LSU-Lb              TACACCGGTTATGTACATAGAGAGTATAAGGGTATCGGGGAGAGGTAG
Ar2-Lb              ------------------------------------------------
As2-Lb              ------------------------------------------------
```

sca0 **sequences**

As5-Lb, *Rickettsia felis* clone As5 (GQ329880).

```
Cal2                TTGCAAGCAGTGTTAATGCAAAAGGTGCGGCGACGCTCGGCGATACTACAAGTTTTGCCG
LSU                 TTGCAAGCAGTGTTAATGCAAAAGGTGCGGCGACGCTCGGCGATACTACAAGTTTTGCCG
LSU-Lb              TTGCAAGCAGTGTTAATGCAAAAGGTGCGGCGACGCTCGGCGATACTACAAGTTTTGCCG
As5-Lb              TTGCAAGCAGTGTTAATGCAAAAGGTGCGGCGACGCTCGGCGATACTACAAGTTTTGCCG
                    ************************************************************

Cal2                ATATATTTACTAACACCGGTGCAGTTACTTTAGCTAAAGCTTCTATCACTAATTTTGCTA
LSU                 ATATATTTACTAACACCGGTGCAGTTACTTTAGCTAAAGCTTCTATCACTAATTTTGCTA
LSU-Lb              ATATATTTACTAACACCGGTGCAGTTACTTTAGCTAAAGCTTCTATCACTAATTTTGCTA
As5-Lb              ATATATTTACTAACACCGGTGCAGTTACTTTAGCTAAAGCTTCTATCACTAATTTTGCTA
                    ************************************************************

Cal2                AAAACGTAACGGCTACCAGCTTTGCAGCTAACAATGCTACTATTAATTTCGGTAATAGCC
LSU                 AAAACGTAACGGCTACCAGCTTTGCAGCTAACAATGCTACTATTAATTTCGGTAATAGCC
LSU-Lb              AAAACGTAACGGCTACCAGCTTTGCAGCTAACAATGCTACTATTAATTTCGGTAATAGCC
As5-Lb              AAAACGTAACGGCTACCAGCTTTGCAGCTAACAATGCTACTATTAATTTCGGTAATAGCC
                    ************************************************************

Cal2                TAGCCTTTAATAGTAATATAACAGGTAGCGGTACTACACTTACTTTAGGTGCAAGCCAAG
LSU                 TAGCCTTTAATAGTAATATAACAGGTAGCGGTACTACACTTACTTTAGGTGCAAGCCAAG
LSU-Lb              TAGCCTTTAATAGTAATATAACAGGTAGCGGTACTACACTTACTTTAGGTGCAAGCCAAG
As5-Lb              TAGCCTTTAATAGTAATATAACAGGTAGCGGTACTACACTTACTTTAGGTGCAAGCCAAG
                    ************************************************************

Cal2                TAACATATACCGGCACCGGTAGCTTTACTGATACGCTAACCTTAAATACTACTTTTGACG
LSU                 TAACATATACCGGCACCGGTAGCTTTACTGATACGCTAACCTTAAATACTACTTTTGACG
LSU-Lb              TAACATATACCGGCACCGGTAGCTTTACTGATACGCTAACCTTAAATACTACTTTTGACG
As5-Lb              TAACATATACCGGCACCGGTAGCTTTACTGATACGCTAACCTTAAATACTACTTTTGACG
                    ************************************************************

Cal2                GAGCAGCTAAGTCAGGCGGTAATATCTTAATTAAATCAGGTAGTACTCTTGATTTATCAG
LSU                 GAGCAGCTAAGTCAGGCGGTAATATCTTAATTAAATCAGGTAGTACTCTTGATTTATCAG
LSU-Lb              GAGCAGCTAAGTCAGGCGGTAATATCTTAATTAAATCAGGTAGTACTCTTGATTTATCAG
As5-Lb              GAGCAGCTAAGTCAGGCGGTAATATCTTAATTAAATCAGGTAGTACTCTTGATTTATCAG
                    ************************************************************
```

```
Cal2           GGGTTTCAACTTTAGCACTTGTTGTTACTGCTACTAATTTTGACATTAATAATATAAGCC
LSU            GGGTTTCAACTTTAGCACTTGTTGTTACTGCTACTAATTTTGACATTAATAATATAAGCC
LSU-Lb         GGGTTTCAACTTTAGCACTTGTTGTTACTGCTACTAATTTTGACATTAATAATATAAGCC
As5-Lb         GGGTTTCAACTTTAGCACTTGTTGTTACTGCTACTAATTTTGACATTAATAATATAAGCC
               ************************************************************

Cal2           CGGATACAAAATATACGGTAATATCTGCAGAAACGGAAGGCGGTTTAAAGCCTACTCCCG
LSU            CGGATACAAAATATACGGTAATATCTGCAGAAACGGAAGGCGGTTTAAAGCCTACTCCCG
LSU-Lb         CGGATACAAAATATACGGTAATATCTGCAGAAACGGAAGGCGGTTTAAAGCCTACTCCCG
As5-Lb         CGGATACAAAATATACGGTAATATCTGCAGAAACGGAAGGCGGTTTAAAGCCTACTCCCG
               ************************************************************

Cal2           AAGAGAATGTTAAAATAACTATTAACAATGATAACCGTTTTGTTGACTTTACTTTTGATG
LSU            AAGAGAATGTTAAAATAACTATTAACAATGATAACCGTTTTGTTGACTTTACTTTTGATG
LSU-Lb         AAGAGAATGTTAAAATAACTATTAACAATGATAACCGTTTTGTTGACTTTACTTTTGATG
As5-Lb         AAGAGAATGTTAAAATAACTATTAACAATGATAACCGTTTTGTTGACTTTACTTTTGATG
               ************************************************************

Cal2           CATCGACTTTAACGTTATTTGCTGAAGATATAGCTGAAGATGTTATAGATGAAGATTTTG
LSU            CATCGACTTTAACGTTATTTGCTGAAGATATAGCTGAAGATGTTATAGATGAAGATTTTG
LSU-Lb         CATCGACTTTAACGTTATTTGCTGAAGATATAGCTGAAGATGTTATAGATGAAGATTTTG
As5-Lb         CATCGACTTTAACGTTATTTGCTGAAGATATAGCTGAAGATGTTATAGATGAAGATTTTG
               ************************************************************

Cal2           AACCAGGCGGACCGCTTGCAAATATCCCGAATGCTGCAAATATAAAGAAATCGCTTGAGT
LSU            AACCAGGCGGACCGCTTGCAAATATCCCGAATGCTGCAAATATAAAGAAATCGCTTGAGT
LSU-Lb         AACCAGGCGGACCGCTTGCAAATATCCCGAATGCTGCAAATATAAAGAAATCGCTTGAGT
As5-Lb         AACCAGGCGGACCGCTTGCAAATATCCCGAATGCTGCAAATATAAAGAAATCGCTTGAGT
               ************************************************************

Cal2           TAATGGAAGATGCTCCAAACGGTTCGGATGTACGTCAAGCTTTCAATAATTTCGGCTTTA
LSU            TAATGGAAGATGCTCCAAACGGTTCGGATGTACGTCAAGCTTTCAATAATTTCGGCTTTA
LSU-Lb         TAATGGAAGATGCTCCAAACGGTTCGGATGTACGTCAAGCTTTCAATAATTTCGGCTTTA
As5-Lb         TAATGGAAGATGCTCCAAACGGTTCGGATGTACGTCAAGCTTTCAATAATTTCGGCTTTA
               ************************************************************

Cal2           TGACGCCGCAACAAGAAGCAGATGCTGTAACTCACCTTATACAAGATGTTGTAAAACCTA
LSU            TGACGCCGCAACAAGAAGCAGATGCTGTAACTCACCTTATACAAGATGTTGTAAAACCTA
LSU-Lb         TGACGCCGCAACAAGAAGCAGATGCTGTAACTCACCTTATACAAGATGTTGTAAAACCTA
As5-Lb         TGACGCCGCAACAAGAAGCAGATGCTGTAACTCACCTTATACAAGATGTTGTAAAACCTA
               ************************************************************

Cal2           GCGATACTATAGCTGCTATTAATAATCAAGTTATACTAAGTAATATCTCAAGTAGCTTAA
LSU            GCGATACTATAGCTGCTATTAATAATCAAGTTATACTAAGTAATATCTCAAGTAGCTTAA
LSU-Lb         GCGATACTATAGCTGCTATTAATAATCAAGTTATACTAAGTAATATCTCAAGTAGCTTAA
As5-Lb         GCGATACTATAGCTGCTATTAATAATCAAGTTATACTAAGTAATATCTCAAGTAGCTTAA
               ************************************************************

Cal2           TTAATCTAAATGCTAGAATGGATGCAATACAGCCCGGCTGCCGTAGCTGCCGGTGATGAG
LSU            TTAATCTAAATGCTAGAATGGATGCAATACAGCCCGGCTGCCGTAGCTGCCGGTGATGAG
LSU-Lb         TTAATCTAAATGCTAGAATGGATGCAATACAGCCCGGCTGCCGTAGCTGCCGGTGATGAG
As5-Lb         TTAATCTAAATGCTAGAATGGATGCAATACAGCCCGGCTGCCGTAGCTGCCGGTGATGAG
               ************************************************************

Cal2           GACAGAGATGCTAAGTTTGGTGCATGGATAAGTCCGTTTGTCGGTAATGCAACGCAGAAG
LSU            GACAGAGATGCTAAGTTTGGTGCATGGATAAGTCCGTTTGTCGGTAATGCAACGCAGAAG
LSU-Lb         GACAGAGATGCTAAGTTTGGTGCATGGATAAGTCCGTTTGTCGGTAATGCAACGCAGAAG
As5-Lb         GACAGAGATGCTAAGTTTGGTGCATGGATAAGTCCGTTTGTCGGTAATGCAACGCAGAAG
               ************************************************************


Cal2           ATGCGTAACAATATAAGCGGTTATAAGTCTGATACAACCGGTGGCACTATAGGTTTTGAC
LSU            ATGCGTAACAATATAAGCGGTTATAAGTCTGATACAACCGGTGGCACTATAGGTTTTGAC
LSU-Lb         ATGCGTAACAATATAAGCGGTTATAAGTCTGATACAACCGGTGGCACTATAGGTTTTGAC
As5-Lb         ATGCGTAACAATATAAGCGGTTATAAGTCTGATACAACCGGTGGCACTATAGGTTTTGAC
               ************************************************************

Cal2           GGCTTAGTTAACGATGATTTAGCACTCGGGCTTGCATATACAAGAGCCGATACCGATATT
LSU            GGCTTAGTTAACGATGATTTAGCACTCGGGCTTGCATATACAAGAGCCGATACCGATATT
LSU-Lb         GGCTTAGTTAACGATGATTTAGCACTCGGGCTTGCATATACAAGAGCCGATACCGATATT
As5-Lb         GGCTTAGTTAACGATGATTTAGCACTCGGGCTTGCATATACAAGAGCCGATACCGATATT
               ************************************************************
```

```
Cal2            AAGCTGAAAAATAATAAGACGGGCGATAAGAATAAGGTAGAGAGTAACATCTATTCCGTA
LSU             AAGCTGAAAAATAATAAGACGGGCGATAAGAATAAGGTAGAGAGTAACATCTATTCCGTA
LSU-Lb          AAGCTGAAAAATAATAAGACGGGCGATAAGAATAAGGTAGAGAGTAACATCTATTCCGTA
As5-Lb          AAGCTGAAAAATAATAAGACGGGCGATAAGAATAAGGTAGAGAGTAACATCTATTCCGTA
                ************************************************************

Cal2            TACGGTTTATATAATGTACCTTATGAAAATCTTTTCGTTGAAGCTATAGCATCTTACTCG
LSU             TACGGTTTATATAATGTACCTTATGAAAATCTTTTCGTTGAAGCTATAGCATCTTACTCG
LSU-Lb          TACGGTTTATATAATGTACCTTATGAAAATCTTTTCGTTGAAGCTATAGCATCTTACTCG
As5-Lb          TACGGTTTATATAATGTACCTTATGAAAATCTTTTCGTTGAAGCTATAGCATCTTACTCG
                ************************************************************

Cal2            GATAATAGGATAAAAAGCAAATCAAGACGTGTTATTGCAACGGCACTAGAGACTGTCGGC
LSU             GATAATAGGATAAAAAGCAAATCAAGACGTGTTATTGCAACGGCACTAGAGACTGTCGGC
LSU-Lb          GATAATAGGATAAAAAGCAAATCAAGACGTGTTATTGCAACGGCACTAGAGACTGTCGGC
As5-Lb          GATAATAGGATAAAAAGCAAATCAAGACGTGTTATTGCAACGGCACTAGAGACTGTCGGC
                ************************************************************

Cal2            TATCAAACCGCAAGCGGTAAGTATAAATCTGAAAGCTATACAGGTCAGTTAATGGCTGGT
LSU             TATCAAACCGCAAGCGGTAAGTATAAATCTGAAAGCTATACAGGTCAGTTAATGGCTGGT
LSU-Lb          TATCAAACCGCAAGCGGTAAGTATAAATCTGAAAGCTATACAGGTCAGTTAATGGCTGGT
As5-Lb          TATCAAACCGCAAGCGGTAAGTATAAATCTGAAAGCTATACAGGTCAGTTAATGGCTGGT
                ************************************************************

Cal2            TATACCTATATGATGCCTGAGAATATTAACTTAACACCGCTTGCAGGGCTTAGATATTCG
LSU             TATACCTATATGATGCCTGAGAATATTAACTTAACACCGCTTGCAGGGCTTAGATATTCG
LSU-Lb          TATACCTATATGATGCCTGAGAATATTAACTTAACACCGCTTGCAGGGCTTAGATATTCG
As5-Lb          TATACCTATATGATGCCTGAGAATATTAACTTAACACCGCCTGCAGGGCTTAGATATTCG
                **************************************** *******************

Cal2            GCTATCAAAGATAAGGGCTATAAGGAAACTGACTACTAACCAAAACCTTATAGTTAAAGG
LSU             GCTATCAAAGATAAGGGCTATAAGGAAACTGACTACTAACCAAAACCTTATAGTTAAAGG
LSU-Lb          GCTATCAAAGATAAGGGCTATAAGGAAACTGACTACTAACCAAAACCTTATAGTTAAAGG
As5-Lb          GCTATCAAAGATAAGGGCTATAAGGAAACTGACTACTAACCAAAACCTTATAGTTAAAGG
                ************************************************************

Cal2            CAAGAACTATAATAGCTTCGATGGTTTACTCGGCGGTAAAGTATCAAGTAATATCAATGT
LSU             CAAGAACTATAATAGCTTCGATGGTTTACTCGGCGGTAAAGTATCAAGTAATATCAATGT
LSU-Lb          CAAGAACTATAATAGCTTCGATGGTTTACTCGGCGGTAAAGTATCAAGTAATATCAATGT
As5-Lb          CAAGAACTATAATAGCTTCGATGGTTTACTCGGCGGTAAAGTATCAAGTAATATCAATGT
                ************************************************************

Cal2            CAATGAAGAAGTAGTGCTAACACCTGAGCTTTACGCAATGGTTGATTATGCATTCAAGAA
LSU             CAATGAAGAAGTAGTGCTAACACCTGAGCTTTACGCAATGGTTGATTATGCATTCAAGAA
LSU-Lb          CAATGAAGAAGTAGTGCTAACACCTGAGCTTTACGCAATGGTTGATTATGCATTCAAGAA
As5-Lb          CAATGAAGAAGTAGTGCTAACACCTGAGCTTTACGCAATGGTTGATTATGCATTCAAGAA
                ************************************************************

Cal2            TAAAGTTCCGGCGATTGATGCAAGATTACAAGGTATGACTGCTCCTTTACCAACAGCTTT
LSU             TAAAGTTCCGGCGATTGATGCAAGATTACAAGGTATGACTGCTCCTTTACCAACAGCTTT
LSU-Lb          TAAAGTTCCGGCGATTGATGCAAGATTACAAGGTATGACTGCTCCTTTACCAACAGCTTT
As5-Lb          TAAAGTTCCGGCGATTGATGCAAGATTACAAGGTATGACTGCTCCTTTACCAACAGCTTT
                ************************************************************

Cal2            AAGCAAAGCAAAACAAGCTTTGATGTCGGCGTCGGTGTTACTGCTAAGCACAAAATGATG
LSU             AAGCAAAGCAAAACAAGCTTTGATGTCGGCGTCGGTGTTACTGCTAAGCACAAAATGATG
LSU-Lb          AAGCAAAGCAAAACAAGCTTTGATGTCGGCGTCGGTGTTACTGCTAAGCACAAAATGATG
As5-Lb          AAGCAAAGCAAAACAAGCTTTGATGTCGGCGTCGG-GTTACTGCTAAGCACAAAATGATG
                *********************************** ************************

Cal2            GAATACGGTATTAACTACGATACAA
LSU             GAATACGGTATTAACTACGATACAA
LSU-Lb          GAATACGGTATTAACTACGATACAA
As5-Lb          GAATACGGTATTAACTACGATACAA
                *************************
```

sca4 **sequences**

As3-Lb, *Rickettsia felis* clone As3 (GQ329878); AF1-Lb, *Rickettsia felis* strain AF1 (HM626232); AM1-Lb, *Rickettsia felis* strain AM1 (HM626231).

```
Cal2          ATGAGTAAAGATAGTGATAATCCTGGATATGAAAGTGGATATGAGTCAGACACCGAAG-A
LSU           ----------------------------ATGAAAGTGGATATGAGTCAGACACCGAAGAA
LSU-lb        ----------------------------ATGAAAGTGGATATGAGTCAGACACCGAAGAA
As3-Lb        ------------------------------------------------------------
AF1-Lb        ------------------------------------------------------------
AM1-Lb        ------------------------------------------------------------

Cal2          AAAAAAACAAGAGCAAGCTGTTCCTGCTCAACCTATTAGTAGTACTGCAAATAAAGACGG
LSU           AAAAAAACAAGAGCAAGCTGTTCCTGCTCAACCTATTAGTAGTACTGCAAATAAAGACGG
LSU-lb        AAAAAAACAAGAGCAAGCTGTTCCTGCTCAACCTATTAGTAGTACTGCAAATAAAGACGG
As3-Lb        ------------------------------------------------------------
AF1-Lb        ------------------------------------------------------------
AM1-Lb        ------------------------------------------------------------

Cal2          TAACCCCGATACAAGTGAGTTTGATCCTTTAGCAAATAAGGAATATACAGAAGAACAAAA
LSU           TAACCCCGATACAAGTGAGTTTGATCCTTTAGCAAATAAGGAATATACAGAAGAACAAAA
LSU-lb        TAACCCCGATACAAGTGAGTTTGATCCTTTAGCAAATAAGGAATATACAGAAGAACAAAA
As3-Lb        -----------------------------------------------CAGAAGAACAAAA
AF1-Lb        ------------------------------------------------------------
AM1-Lb        ------------------------------------------------------------

Cal2          GCAAAAATTAGAACAAGAACAAAAAGAGTATTTTTCTCAAACTACACCACAAGAACTAGA
LSU           GCAAAAATTAGAACAAGAACAAAAAGAGTATTTTTCTCAAACTACACCACAAGAACTAGA
LSU-lb        GCAAAAATTAGAACAAGAACAAAAAGAGTATTTTTCTCAAACTACACCACAAGAACTAGA
As3-Lb        GCAAAAATTAGAACAAGAACAAAAAGAGTATTTTTCTCAAACTACACCACAAGAACTAGA
AF1-Lb        ------------------------------------------------------------
AM1-Lb        ------------------------------------------------------------

Cal2          AGCTGATGATGGTTTTAGCTTTACTCCTGCATCTTCTACTCAATCTACTCCTTCAATCAG
LSU           AGCTGATGATGGTTTTAGCTTTACTCCTGCATCTTCTACTCAATCTACTCCTTCAATCAG
LSU-lb        AGCTGATGATGGTTTTAGCTTTACTCCTGCATCTTCTACTCAATCTACTCCTTCAATCAG
As3-Lb        AGCTGATGATGGTTTTAGCTTTACTCCTGCATCTTCTACTCAATCTACTCCTTCAATCAG
AF1-Lb        ------------------------------------------------------------
AM1-Lb        ------------------------------------------------------------

Cal2          CAGCTTATCGGGCGGTATTTCCTCTGACAGTCAGACATCAGACCCAATAACCAAGGCTGT
LSU           CAGCTTATCGGGCGGTATTTCCTCTGACAGTCAGACATCAGACCCAATAACCAAGGCTGT
LSU-lb        CAGCTTATCGGGCGGTATTTCCTCTGACAGTCAGACATCAGACCCAATAACCAAGGCTGT
As3-Lb        CAGCTTATCGGGCGGTATTTCCTCTGACAGTCAGACATCAGACCCAATAACCAAGGCTGT
AF1-Lb        ------------------------------------------------------------
AM1-Lb        ------------------------------------------------------------

Cal2          AAGAGAAACAATTATACAACCACAAAAAGATGAGATAGCAGAGCAAATATTAAAAGACCT
LSU           AAGAGAAACAATTATACAACCACAAAAAGATGAGATAGCAGAGCAAATATTAAAAGACCT
LSU-lb        AAGAGAAACAATTATACAACCACAAAAAGATGAGATAGCAGAGCAAATATTAAAAGACCT
As3-Lb        AAGAGAAACAATTATACAACCACAAAAAGATGAGATAGCAGAGCAAATATTAAAAGACCT
AF1-Lb        ------------------------------------------------------------
AM1-Lb        ------------------------------------------------------------

Cal2          AGCAGCCCTTGCAGACCGTGATTTAGCTGAACAAAAAAGAAAAGAAATAGAAGAGGAAAA
LSU           AGCAGCCCTTGCAGACCGTGATTTAGCTGAACAAAAAAGAAAAGAAATAGAAGAGGAAAA
LSU-lb        AGCAGCCCTTGCAGACCGTGATTTAGCTGAACAAAAAAGAAAAGAAATAGAAGAGGAAAA
As3-Lb        AGCAGCCCTTGCAGACCGTGATTTAGCTGAACAAAAAAGAAAAGAAATAGAAGAGGAAAA
AF1-Lb        ------------------------------------------------------------
AM1-Lb        ------------------------------------------------------------

Cal2          AGATAAAACATTAAGTGCTTTTTTCGGTAATCCGGCTAATAGAGAATTTATTGATAAGGC
LSU           AGATAAAACATTAAGTGCTTTTTTCGGTAATCCGGCTAATAGAGAATTTATTGATAAGGC
LSU-lb        AGATAAAACATTAAGTGCTTTTTTCGGTAATCCGGCTAATAGAGAATTTATTGATAAGGC
As3-Lb        AGATAAAACATTAAGTGCTTTTTTCGGTAATCCGGCTAATAGAGAATTTATTGATAAGGC
AF1-Lb        ------------------------------------------------------------
AM1-Lb        ------------------------------------------------------------
```

```
Cal2            TTTAGAAAATCCTGAACTTAAAAAGAAATTAGAATCAATAGAAATAGCTGGCTATAAAAA
LSU             TTTAGAAAATCCTGAACTTAAAAAGAAATTAGAATCAATAGAAATAGCTGGCTATAAAAA
LSU-lb          TTTAGAAAATCCTGAACTTAAAAAGAAATTAGAATCAATAGAAATAGCTGGCTATAAAAA
As3-Lb          TTTAGAAAATCCTGAACTTAAAAAGAAATTAGAATCAATAGAAATAGCTGGCTATAAAAA
AF1-Lb          ------------------------------------------------------------
AM1-Lb          ------------------------------------------------------------

Cal2            TGTTCTCTCAACATATAGTGCCGCTAATGGATATCAGGGTGGATTTAAGCCCGTACAGTG
LSU             TGTTCTCTCAACATATAGTGCCGCTAATGGATATCAGGGTGGATTTAAGCCCGTACAGTG
LSU-lb          TGTTCTCTCAACATATAGTGCCGCTAATGGATATCAGGGTGGATTTAAGCCCGTACAGTG
As3-Lb          TGTTCTCTCAACATATAGTGCCGCTAATGGATATCAGGGTGGATTTAAGCCCGTACAGTG
AF1-Lb          ------------------------------------------------------------
AM1-Lb          ------------------------------------------------------------

Cal2            GGAAAACCAAATAAGTGCAAGCGATCTTAGAGCCACGGTAGTTAGAAATGATGCAGGCGA
LSU             GGAAAACCAAATAAGTGCAAGCGATCTTAGAGCCACGGTAGTTAGAAATGATGCAGGCGA
LSU-lb          GGAAAACCAAATAAGTGCAAGCGATCTTAGAGCCACGGTAGTTAGAAATGATGCAGGCGA
As3-Lb          GGAAAACCAAATAAGTGCAAGCGATCTTAGAGCCACGGTAGTTAGAAATGATGCAGGCGA
AF1-Lb          ------------------------------------------------------------
AM1-Lb          ------------------------------------------------------------

Cal2            TGAACTCTGTACCTTAAATGAAACAACTGTTAAAACTAAGCCTTTTACTGTAGCTAAACA
LSU             TGAACTCTGTACCTTAAATGAAACAACTGTTAAAACTAAGCCTTTTACTGTAGCTAAACA
LSU-lb          TGAACTCTGTACCTTAAATGAAACAACTGTTAAAACTAAGCCTTTTACTGTAGCTAAACA
As3-Lb          TGAACTCTGTACCTTAAATGAAACAACTGTTAAAACTAAGCCTTTTACTGTAGCTAAACA
AF1-Lb          ------------------------------------------------------------
AM1-Lb          ------------------------------------------------------------

Cal2            AGACGGTACTCAGGTTCAAATCAATTCATATAGAGAAATAGATTTTCCTATAAAACTTGA
LSU             AGACGGTACTCAGGTTCAAATCAATTCATATAGAGAAATAGATTTTCCTATAAAACTTGA
LSU-lb          AGACGGTACTCAGGTTCAAATCAATTCATATAGAGAAATAGATTTTCCTATAAAACTTGA
As3-Lb          AGACGGTACTCAGGTTCAAATCAATTCATATAGAGAAATAGATTTTCCTATAAAACTTGA
AF1-Lb          ------------------------------------------------------------
AM1-Lb          ------------------------------------------------------------

Cal2            TAAAGCCGATGGGTCAATGCATCTATCAATGGTAGCATTAAAAGCTGATGGCACAAAGCC
LSU             TAAAGCCGATGGGTCAATGCATCTATCAATGGTAGCATTAAAAGCTGATGGCACAAAGCC
LSU-lb          TAAAGCCGATGGGTCAATGCATCTATCAATGGTAGCATTAAAAGCTGATGGCACAAAGCC
As3-Lb          TAAAGCCGATGGGTCAATGCATCTATCAATGGTAGCATTAAAAGCTGATGGCACAAAGCC
AF1-Lb          ------------------------------------------------------------
AM1-Lb          ------------------------------------------------------------

Cal2            CTCTAAAGATAAAGCAGTATATTTCACTGCTCACTACGAAGAAGGACCAAACGGTAAACC
LSU             CTCTAAAGATAAAGCAGTATATTTCACTGCTCACTACGAAGAAGGACCAAACGGTAAACC
LSU-lb          CTCTAAAGATAAAGCAGTATATTTCACTGCTCACTACGAAGAAGGACCAAACGGTAAACC
As3-Lb          CTCTAAAGATAAAGCAGTATATTTCACTGCTCACTACGAAGAAGGACCAAACGGTAAACC
AF1-Lb          ------------------------------------------------------------
AM1-Lb          ------------------------------------------------------------

Cal2            TCAACTTAAAGAAATAAGCTCACCGAAACCTTTAAAATTTGCCGGAGACGGACCGGATGC
LSU             TCAACTTAAAGAAATAAGCTCACCGAAACCTTTAAAATTTGCCGGAGACGGACCGGATGC
LSU-lb          TCAACTTAAAGAAATAAGCTCACCGAAACCTTTAAAATTTGCCGGAGACGGACCGGATGC
As3-Lb          TCAACTTAAAGAAATAAGCTCACCGAAACCTTTAAAATTTGCCGGAGACGGACCGGATGC
AF1-Lb          ------------------------------------------------------------
AM1-Lb          ------------------------------------------------------------

Cal2            GGTAGCTTATATTGAGCATGGCGGAGAAATTTATACACTTGCGGTAACACGCGGTAAATA
LSU             GGTAGCTTATATTGAGCATGGCGGAGAAATTTATACACTTGCGGTAACACGCGGTAAATA
LSU-lb          GGTAGCTTATATTGAGCATGGCGGAGAAATTTATACACTTGCGGTAACACGCGGTAAATA
As3-Lb          GGTAGCTTATATTGAGCATGGCGGAGAAATTTATACACTTGCGGTAACACGCGGTAAATA
AF1-Lb          ------------------------------------------------------------
AM1-Lb          ------------------------------------------------------------
```

```
Cal2                TAAAGAAATGATGAGAGAGGTAGAACTAAACCAAGGGCAGAGCGTTGATTTATCACAAAC
LSU                 TAAAGAAATGATGAGAGAGGTAGAACTAAACCAAGGGCAGAGCGTTGATTTATCACAAAC
LSU-lb              TAAAGAAATGATGAGAGAGGTAGAACTAAACCAAGGGCAGAGCGTTGATTTATCACAAAC
As3-Lb              TAAAGAAATGATGAGAGAGGTAGAACTAAACCAAGGGCAGAGCGTTGATTTATCACAAAC
AF1-Lb              ------------------------------------------------------------
AM1-Lb              ------------------------------------------------------------

Cal2                TATAGCTGAAGATTTAACAAAGGTACAAGGTCGATCTCAGGAAACACCACAACCAATAAT
LSU                 TATAGCTGAAGATTTAACAAAGGTACAAGGTCGATCTCAGGAAACACCACAACCAATAAT
LSU-lb              TATAGCTGAAGATTTAACAAAGGTACAAGGTCGATCTCAGGAAACACCACAACCAATAAT
As3-Lb              TATAGCTGAAGATTTAACAAAGGTACAAGGTCGATCTCAGGAAACACCACAACCAATAAT
AF1-Lb              ------------------------------------------------------------
AM1-Lb              ------------------------------------------------------------

Cal2                AACTCCGAATCAAGAATTAAAATCATCTATTGAAACGCCTACCACTACCCAAGTACCGCC
LSU                 AACTCCGAATCAAGAATTAAAATCATCTATTGAAACGCCTACCACTACCCAAGTACCGCC
LSU-lb              AACTCCGAATCAAGAATTAAAATCATCTATTGAAACGCCTACCACTACCCAAGTACCGCC
As3-Lb              AACTCCGAATCAAGAATTAAAATCATCTATTGAAACGCCTACCACTACCCAAGTACCGCC
AF1-Lb              ------------------------------------------------------------
AM1-Lb              ------------------------------------------------------------

Cal2                AATTACTCCTGCCAACCAACCACTGCAACCTGAGACTTCACAAATGCCACAGCCGCAACA
LSU                 AATTACTCCTGCCAACCAACCACTGCAACCTGAGACTTCACAAATGCCACAGCCGCAACA
LSU-lb              AATTACTCCTGCCAACCAACCACTGCAACCTGAGACTTCACAAATGCCACAGCCGCAACA
As3-Lb              AATTACTCCTGCCAACCAACCACTGCAACCTGAGACTTCACAAATGCCACAGCCGCAACA
AF1-Lb              ------------------------------------------------------------
AM1-Lb              ------------------------------------------------------------

Cal2                AGTGAATCCAAACCTCCTTAATGCAGCTACGGCTTTATCAACCAGTATGCAAGATTTATT
LSU                 AGTGAATCCAAACCTCCTTAATGCAGCTACGGCTTTATCAACCAGTATGCAAGATTTATT
LSU-lb              AGTGAATCCAAACCTCCTTAATGCAGCTACGGCTTTATCAACCAGTATGCAAGATTTATT
As3-Lb              AGTGAATCCAAACCTCCTTAATGCAGCTACGGCTTTATCAACCAGTATGCAAGATTTATT
AF1-Lb              ---------AAACCTCCTTAATGCAGCTACGGCTTTATCAACCAGTATGCAAGATTTATT
AM1-Lb              ---------------------------TACGGCTTTATCAACCAGTATGCAAGATTTATT
                                               *********************************

Cal2                AAATTATGTAAATGCAGGTTTAACAAAAGAAAAAGACGGTAATAAACAAATTGATTTAAT
LSU                 AAATTATGTAAATGCAGGTTTAACAAAAGAAAAAGACGGTAATAAACAAATTGATTTAAT
LSU-lb              AAATTATGTAAATGCAGGTTTAACAAAAGAAAAAGACGGTAATAAACAAATTGATTTAAT
As3-Lb              AAATTATGTAAATGCAGGTTTAACAAAAGAAAAAGACGGTAATAAACAAATTGATTTAAT
AF1-Lb              AAATTATGTAAATGCAGGTTTAACAAAAGAAAAAGACGGTAATAAACAAATTGATTTAAT
AM1-Lb              AAATTATGTAAATGCAGGTTTAACAAAAGAAAAAGACGGTAATAAACAAATTGATTTAAT
                    ************************************************************

Cal2                TAACGAAGCAGCCACGGCAATTCTTAATAATGAGAAAAGTGATATTGCTGAAAAGCAGGC
LSU                 TAACGAAGCAGCCACGGCAATTCTTAATAATGAGAAAAGTGATATTGCTGAAAAGCAGGC
LSU-lb              TAACGAAGCAGCCACGGCAATTCTTAATAATGAGAAAAGTGATATTGCTGAAAAGCAGGC
As3-Lb              TAACGAAGCAGCCACGGCAATTCTTAATAATGAGAAAAGTGATATTGCTGAAAAGCAGGC
AF1-Lb              TAACGAAGCAGCCACGGCAATTCTTAATAATGAGAAAAGTGATATTGCTGAAAAGCAGGC
AM1-Lb              TAACGAAGCAGCCACGGCAATTCTTAATAATGAGAAAAGTGATATTGCTGAAAAGCAGGC
                    ************************************************************

Cal2                TAATATCATTGCTTTAACTGAAAATACGGTCAATAATAACGACCTCACACCGGATACAAA
LSU                 TAATATCATTGCTTTAACTGAAAATACGGTCAATAATAACGACCTCACACCGGATACAAA
LSU-lb              TAATATCATTGCTTTAACTGAAAATACGGTCAATAATAACGACCTCACACCGGATACAAA
As3-Lb              TAATATCATTGCTTTAACTGAAAATACGGTCAATAATAACGACCTCACACCGGATACAAA
AF1-Lb              TAATATCATTGCTTTAACTGAAAATACGGTCAATAATAACGACCTCACACCGGATACAAA
AM1-Lb              TAATATCATTGCTTTAACTGAAAATACGGTCAATAATAACGACCTCACACCGGATACAAA
                    ************************************************************

Cal2                AGTAGCTGGAGTAAATGCGGTATTAGAAACCATAAAAAATGATCAGAATACTCCAGACCT
LSU                 AGTAGCTGGAGTAAATGCGGTATTAGAAACCATAAAAAATGATCAGAATACTCCAGACCT
LSU-lb              AGTAGCTGGAGTAAATGCGGTATTAGAAACCATAAAAAATGATCAGAATACTCCAGACCT
As3-Lb              AGTAGCTGGAGTAAATGCGGTATTAGAAACCATAAAAAATGATCAGAATACTCCAGACCT
AF1-Lb              AGTAGCTGGAGTAAATGCGGTATTAGAAACCATAAAAAATGATCAGAATACTCCAGACCT
AM1-Lb              AGTAGCTGGAGTAAATGCGGTATTAGAAACCATAAAAAATGATCAGAATACTCCAGACCT
                    ************************************************************
```

```
Cal2            AGAAAAATCAAAAATGCTTGAAGCTACAGTAGCTATAGCTTTAAATTCAGAGAATCTTGA
LSU             AGAAAAATCAAAAATGCTTGAAGCTACAGTAGCTATAGCTTTAAATTCAGAGAATCTTGA
LSU-lb          AGAAAAATCAAAAATGCTTGAAGCTACAGTAGCTATAGCTTTAAATTCAGAGAATCTTGA
As3-Lb          AGAAAAATCAAAAATGCTTGAAGCTACAGTAGCTATAGCTTTAAATTCAGAGAATCTTGA
AF1-Lb          AGAAAAATCAAAAATGCTTGAAGCTACAGTAGCTATAGCTTTAAATTCAGAGAATCTTGA
AM1-Lb          AGAAAAATCAAAAATGCTTGAAGCTACAGTAGCTATAGCTTTAAATTCAGAGAATCTTGA
                ************************************************************

Cal2            ACCGAAGCAAAAACAGCAGATGTTAGAAAAGGCAGTAGATGTCGGTTTAAGTCTTAAAGA
LSU             ACCGAAGCAAAAACAGCAGATGTTAGAAAAGGCAGTAGATGTCGGTTTAAGTCTTAAAGA
LSU-lb          ACCGAAGCAAAAACAGCAGATGTTAGAAAAGGCAGTAGATGTCGGTTTAAGTCTTAAAGA
As3-Lb          ACCGAAGCAAAAACAGCAGATGTTAGAAAAGGCAGTAGATGTCGGTTTAAGTCTTAAAGA
AF1-Lb          ACCGAAGCAAAAACAGCAGATGTTAGAAAAGGCAGTAGATGTCGGTTTAAGTCTTAAAGA
AM1-Lb          ACCGAAGCAAAAACAGCAGATGTTAGAAAAGGCAGTAGATGTCGGTTTAAGTCTTAAAGA
                ************************************************************

Cal2            TGATGCAAGTAGAGTTACGGCAATTGACGGTATTACGGATGCTGTAATAAAAAGTAACCT
LSU             TGATGCAAGTAGAGTTACGGCAATTGACGGTATTACGGATGCTGTAATAAAAAGTAACCT
LSU-lb          TGATGCAAGTAGAGTTACGGCAATTGACGGTATTACGGATGCTGTAATAAAAAGTAACCT
As3-Lb          TGATGCAAGTAGAGTTACGGCAATTGACGGTATTACGGATGCTGTAATAAAAAGTAACCT
AF1-Lb          TGATGCAAGTAGAGTTACGGCAATTGACGGTATTACGGATGCTGTAATAAAAAGTAACCT
AM1-Lb          TGATGCAAGTAGAGTTACGGCAATTGACGGTATTACGGATGCTGTAATAAAAAGTAACCT
                ************************************************************

Cal2            TTCTACTGAAGATAAAGGGACAATGCTTATAGCAGTAGGTGATAAGGTTAATGCCTCTGA
LSU             TTCTACTGAAGATAAAGGGACAATGCTTATAGCAGTAGGTGATAAGGTTAATGCCTCTGA
LSU-lb          TTCTACTGAAGATAAAGGGACAATGCTTATAGCAGTAGGTGATAAGGTTAATGCCTCTGA
As3-Lb          TTCTACTGAAGATAAAGGGACAATGCTTATAGCAGTAGGTGATAAGGTTAATGCCTCTGA
AF1-Lb          TTCTACTGAAGATAAAGGGACAATGCTTATAGCAGTAGGTGATAAGGTTAATGCCTCTGA
AM1-Lb          TTCTACTGAAGATAAAGGGACAATGCTTATAGCAGTAGGTGATAAGGTTAATGCCTCTGA
                ************************************************************

Cal2            ATTAAGCAATGCGGAAAAACAAAAATTATTAGGTTCTGTATTAAAGAAAGGTGTAGAAGC
LSU             ATTAAGCAATGCGGAAAAACAAAAATTATTAGGTTCTGTATTAAAGAAAGGTGTAGAAGC
LSU-lb          ATTAAGCAATGCGGAAAAACAAAAATTATTAGGTTCTGTATTAAAGAAAGGTGTAGAAGC
As3-Lb          ATTAAGCAATGCGGAAAAACAAAAATTATTAGGTTCTGTATTAAAGAAAGGTGTAGAAGC
AF1-Lb          ATTAAGCAATGCGGAAAAACAAAAATTATTAGGTTCTGTATTAAAGAAAGGTGTAGAAGC
AM1-Lb          ATTAAGCAATGCGGAAAAACAAAAATTATTAGGTTCTGTATTAAAGAAAGGTGTAGAAGC
                ************************************************************

Cal2            CCAAGTTCTCAGTCCGGAACAACAACAATTGATGCAGCAGAATTTAGATAAGATTACAGC
LSU             CCAAGTTCTCAGTCCGGAACAACAACAATTGATGCAGCAGAATTTAGATAAGATTACAGC
LSU-lb          CCAAGTTCTCAGTCCGGAACAACAACAATTGATGCAGCAGAATTTAGATAAGATTACAGC
As3-Lb          CCAAGTTCTCAGTCCGGAACAACAACAATTGATGCAGCAGAATTTAGATAAGATTACAGC
AF1-Lb          CCAAGTTCTCAGTCCGGAACAACAACAATTGATGCAGCAGAATTTAGATAAGATTACAGC
AM1-Lb          CCAAGTTCTCAGTCCGGAACAACAACAATTGATGCAGCAGAATTTAGATAAGATTACAGC
                ************************************************************

Cal2            AGAACAAACTAAAAATGCCCAAATAACAGAGGTACAAGGTATTTTAGCAAACCCTGCATT
LSU             AGAACAAACTAAAAATGCCCAAATAACAGAGGTACAAGGTATTTTAGCAAACCCTGCATT
LSU-lb          AGAACAAACTAAAAATGCCCAAATAACAGAGGTACAAGGTATTTTAGCAAACCCTGCATT
As3-Lb          AGAACAAACTAAAAATGCCCAAATAACAGAGGTACAAGGTATTTTAGCAAACCCTGCATT
AF1-Lb          AGAACAAACTAAAAATGCCCAAATAACAGAGGTACAAGGTATTTTAGCAAACCCTGCATT
AM1-Lb          AGAACAAACTAAAAATGCCCAAATAACAGAGGTACAAGGTATTTTAGCAAACCCTGCATT
                ************************************************************

Cal2            TAATACTATCGCTAAAACTGAGGCAATACAAAATGTTACTACAAAGGTTTTAGATAGTCC
LSU             TAATACTATCGCTAAAACTGAGGCAATACAAAATGTTACTACAAAGGTTTTAGATAGTCC
LSU-lb          TAATACTATCGCTAAAACTGAGGCAATACAAAATGTTACTACAAAGGTTTTAGATAGTCC
As3-Lb          TAATACTATCGCTAAAACTGAGGCAATACAAAATGTTACTACAAAGGTTTTAGATAGTCC
AF1-Lb          TAATACTATCGCTAAAACTGAGGCAATACAAAATGTTACTACAAAGGTTTTAGATAGTCC
AM1-Lb          TAATACTATCGCTAAAACTGAGGCAATACAAAATGTTACTACAAAGGTTTTAGATAGTCC
                ************************************************************

Cal2            GATTAAAGCAGAAATAAAAGGTGAAACGCTCGAAAGTATTACTAAGGTAGTTGCTGAGAG
LSU             GATTAAAGCAGAAATAAAAGGTGAAACGCTCGAAAGTATTACTAAGGTAGTTGCTGAGAG
LSU-lb          GATTAAAGCAGAAATAAAAGGTGAAACGCTCGAAAGTATTACTAAGGTAGTTGCTGAGAG
As3-Lb          GATTAAAGCAGAAATAAAAGGTGAAACGCTCGAAAGTATTACTAAGGTAGTTGCTGAGAG
AF1-Lb          GATTAAAGCAGAAATAAAAGGTGAAACGCTCGAAAGTATTACTAAGGTAGTTGCTGAGAG
AM1-Lb          GATTAAAGCAGAAATAAAAGGTGAAACGCTCGAAAGTATTACTAAGGTAGTTGCTGAGAG
                ************************************************************
```

```
Cal2           TCCTTTGAACGGTCAAGATAAAGCAGACATTGTTAAAGGTATGGGAGAAGCTATAGCTAG
LSU            TCCTTTGAACGGTCAAGATAAAGCAGACATTGTTAAAGGTATGGGAGAAGCTATAGCTAG
LSU-lb         TCCTTTGAACGGTCAAGATAAAGCAGACATTGTTAAAGGTATGGGAGAAGCTATAGCTAG
As3-Lb         TCCTTTGAACGGTCAAGATA----------------------------------------
AF1-Lb         TCCTTTGAACGGTC----------------------------------------------
AM1-Lb         TCCTTTGAACGGTCAAGA------------------------------------------
               **************

Cal2           TCATAAAACTATGGCACCTACAGAAAAAATTTCTACTATAGAATCCGTAGAAAAAGGGGT
LSU            TCATAAAACTATGGCACCTACAGAAAAAATTTCTACTATAGAATCCGTAGAAAAAGGGGT
LSU-lb         TCATAAAACTATGGCACCTACAGAAAAAATTTCTACTATAGAATCCGTAGAAAAAGGGGT
As3-Lb         ------------------------------------------------------------
AF1-Lb         ------------------------------------------------------------
AM1-Lb         ------------------------------------------------------------

Cal2           GGCCGAAAGTATAACAGATTTGGAAGATAAAAAGTTAATGACTAAAGGATTAGTAGAGGG
LSU            GGCCGAAAGTATAACAGATTTGGAAGATAAAAAGTTAATGACTAAAGGATTAGTAGAGGG
LSU-lb         GGCCGAAAGTATAACAGATTTGGAAGATAAAAAGTTAATGACTAAAGGATTAGTAGAGGG
As3-Lb         ------------------------------------------------------------
AF1-Lb         ------------------------------------------------------------
AM1-Lb         ------------------------------------------------------------

Cal2           TATTTATGAAGGCAAAGCAAATCCTGAAATAACTTCTGAAAAGACTAAAGCTGTTTCTAG
LSU            TATTTATGAAGGCAAAGCAAATCCTGAAATAACTTCTGAAAAGACTAAAGCTGTTTCTAG
LSU-lb         TATTTATGAAGGCAAAGCAAATCCTGAAATAACTTCTGAAAAGACTAAAGCTGTTTCTAG
As3-Lb         ------------------------------------------------------------
AF1-Lb         ------------------------------------------------------------
AM1-Lb         ------------------------------------------------------------

Cal2           AGGGATTGATAAGAGTACTGCTATACCGGAAGATAAACAAGCTCTTAAAGATGCAGCGAA
LSU            AGGGATTGATAAGAGTACTGCTATACCGGAAGATAAACAAGCTCTTAAAGATGCAGCGAA
LSU-lb         AGGGATTGATAAGAGTACTGCTATACCGGAAGATAAACAAGCTCTTAAAGATGCAGCGAA
As3-Lb         ------------------------------------------------------------
AF1-Lb         ------------------------------------------------------------
AM1-Lb         ------------------------------------------------------------

Cal2           TGAGGCAGCTTTAGATAGAGAAACTCAAAATTTAACTGAAGGGTTAAAAAGACAGAATTT
LSU            TGAGGCAGCTTTAGATAGAGAAACTCAAAATTTAACTGAAGGGTTAAAAAGACAGAATTT
LSU-lb         TGAGGCAGCTTTAGATAGAGAAACTCAAAATTTAACTGAAGGGTTAAAAAGACAGAATTT
As3-Lb         ------------------------------------------------------------
AF1-Lb         ------------------------------------------------------------
AM1-Lb         ------------------------------------------------------------

Cal2           AGGAGAACCTAAGCCTCGTGATGATATATATAACAAAGCTCAAGATGTAGCGGATGCATT
LSU            AGGAGAACCTAAGCCTCGTGATGATATATATAACAAAGCTCAAGATGTAGCGGATGCATT
LSU-lb         AGGAGAACCTAAGCCTCGTGATGATATATATAACAAAGCTCAAGATGTAGCGGATGCATT
As3-Lb         ------------------------------------------------------------
AF1-Lb         ------------------------------------------------------------
AM1-Lb         ------------------------------------------------------------

Cal2           -AAAAAATGTTATTACGCCTGTTTTAGATGCTCATCCTGAAAAACGTGAAGTCTCAGAAG
LSU            AAAAAAATGTTATTACGCCTGTTTTAGATGCTCATCCTGA--------------------
LSU-lb         -AAAAAATGTTATTACGCCTGTTTTAGATGCTCATCCTGAAAAACGTGAAGTCTC---AG
As3-Lb         ------------------------------------------------------------
AF1-Lb         ------------------------------------------------------------
AM1-Lb         ------------------------------------------------------------

Cal2           AAGAAGAAGTTGTGAAAAAAACTTCCAGTATATTAAATGATATCTCTAAGCTTGCAATTG
LSU            ------------------------------------------------------------
LSU-lb         AAGAAGAAGTTGTGAAAAAAACTTCCAGTATATTAAATGATATCTCTAAGCTTGCAATTG
As3-Lb         ------------------------------------------------------------
AF1-Lb         ------------------------------------------------------------
AM1-Lb         ------------------------------------------------------------
```

```
Cal2            AGAAAGTCAATAATTTTCGTGCTATGCTCTCTCCAGATGGTAATCTTAAAACTCTTGAAG
LSU             ------------------------------------------------------------
LSU-lb          AGAAAGTCAATAATTTTCGTGCTATGCTCTCTCCAGATGGTAATCTTAAAACTCTTGAAG
As3-Lb          ------------------------------------------------------------
AF1-Lb          ------------------------------------------------------------
AM1-Lb          ------------------------------------------------------------

Cal2            AAAAAAAA-GCTGAATCAACAAAAAAAGTAGATGAGCTGGTAAAGGAATTTGGTACTAAA
LSU             ------------------------------------------------------------
LSU-lb          AAAAAAAAAGCTGA----------------------------------------------
As3-Lb          ------------------------------------------------------------
AF1-Lb          ------------------------------------------------------------
AM1-Lb          ------------------------------------------------------------

Cal2            TCTTCTACTGAAGAACAGCAAAGTTTTATTAAAGCTAATTTAATTGATGATAAAACTTTA
LSU             ------------------------------------------------------------
LSU-lb          ------------------------------------------------------------
As3-Lb          ------------------------------------------------------------
AF1-Lb          ------------------------------------------------------------
AM1-Lb          ------------------------------------------------------------

Cal2            TCTAAAGAGATACGTTTACAAACTATAAATAAGTTATTACAAGAACAAGCACAAAAACGA
LSU             ------------------------------------------------------------
LSU-lb          ------------------------------------------------------------
As3-Lb          ------------------------------------------------------------
AF1-Lb          ------------------------------------------------------------
AM1-Lb          ------------------------------------------------------------

Cal2            GCAGAAGCAATTGAAAACCCTAATGTTAAAACGGAAGATGTAAGGGTAGTATCAGGAGTT
LSU             ------------------------------------------------------------
LSU-lb          ------------------------------------------------------------
As3-Lb          ------------------------------------------------------------
AF1-Lb          ------------------------------------------------------------
AM1-Lb          ------------------------------------------------------------

Cal2            AATATCAAAGATAATATAAAAATTATGGGAGCATTAATGAATGCAAGAGATAGCATTATT
LSU             ------------------------------------------------------------
LSU-lb          ------------------------------------------------------------
As3-Lb          ------------------------------------------------------------
AF1-Lb          ------------------------------------------------------------
AM1-Lb          ------------------------------------------------------------

Cal2            CAGTCGGAAAATTTAAATAAATCAACACCTATTAAAAGAGAGTCTTCCTTTCCGCCACGC
LSU             ------------------------------------------------------------
LSU-lb          ------------------------------------------------------------
As3-Lb          ------------------------------------------------------------
AF1-Lb          ------------------------------------------------------------
AM1-Lb          ------------------------------------------------------------

Cal2            TAA
LSU             ---
LSU-lb          ---
As3-Lb          ---
AF1-Lb          ---
AM1-Lb          ---
```

sca5 sequences

Ar3-Lb, *Rickettsia felis* clone Ar3 (GQ385243); As4-Lb, *Rickettsia felis* clone As4 (GQ329879); Te2-Lb, *Rickettsia felis* clone Te2 (GQ329875).

```
Cal2            ------------------------------------------------------------
LSU             ------------------------------------------------------------
LSU-Lb          ------------------------------------------------------------
Ar3-Lb          ATAAAAATATAACATTTATTAAAACTTTATGAAAAAACTATAGCGATGCTTGTAATTATA
As4-Lb          ------------------------------------------------------------
Te2-Lb          ------------------------------------------------------------


Cal2            ------------------------------------------------------------
LSU             ------------------------------------------------------------
LSU-Lb          ------------------------------------------------------------
Ar3-Lb          TACAATATATAGTATGCTCAGCTCGTAGTTTAGAAACTATTGTAACAAAATATTAGGTTA
As4-Lb          ------------------------------------------------------------
Te2-Lb          ------------------------------------------------------------


Cal2            ------------------------------------------------------------
LSU             ------------------------------------------------------------
LSU-Lb          ------------------------------------------------------------
Ar3-Lb          TTTCCTTATCAAATGCGGGATATATTGACTTGTGTTTGATAAGTTTGTTTTAATACTAGG
As4-Lb          ------------------------------------------------------------
Te2-Lb          ------------------------------------------------------------


Cal2            -----------------------------------ATGGCTCAAAAACCAAATTTTCTAA
LSU             -----------------------------------ATGGCTCAAAAACCAAATTTTCTAA
LSU-Lb          -----------------------------------ATGGCTCAAAAACCAAATTTTCTAA
Ar3-Lb          TACTAAATTTTAACTTAAATATAGGAAAAAAAATTATGGCTCAAAAACCAAATTTTCTAA
As4-Lb          -----------------------------------ATGGCTCAAAAACCAAATTTTCTAA
Te2-Lb          ------------------------------------------------------------


Cal2            AAAAATTAATTTCCGCAGGATTGGTAACTGCTTCTACAGCTACCATAGTAGCCGGCTTCG
LSU             AAAAATTAATTTCCGCAGGATTGGTAACTGCTTCTACAGCTACCATAGTAGCCGGCTTCG
LSU-Lb          AAAAATTAATTTCCGCAGGATTGGTAACTGCTTCTACAGCTACCATAGTAGCCGGCTTCG
Ar3-Lb          AAAAATTAATTTCCGCAGGATTGGTAACTGCTTCTACAGCTACCATAGTAGCCGGCTTCG
As4-Lb          AAAAATTAATTTCCGCAGGATTGGTAACTGCTTCTACAGCTACCATAGTAGCCGGCTTCG
Te2-Lb          ------------CCGCAGGATTGGTAACTGCTTCTACAGCTACCATAGTAGCCGGCTTCG
                            ************************************************

Cal2            CAGGCTCAGCTATGGGTGCTGCTACACAGCAGAATAGAACAACAGTCGGAGCTGCTACAA
LSU             CAGGCTCAGCTATGGGTGCTGCTACACAGCAGAATAGAACAACAGTCGGAGCTGCTACAA
LSU-Lb          CAGGCTCAGCTATGGGTGCTGCTACACAGCAGAATAGAACAACAGTCGGAGCTGCTACAA
Ar3-Lb          CAGGCTCAGCTATGGGTGCTGCTACACAGCAGAATAGAACAACAGTCGGAGCTGCTACAA
As4-Lb          CAGGCTCAGCTATGGGTGCTGCTACACAGCAGAATAGAACAACAGTCGGAGCTGCTACAA
Te2-Lb          CAGGCTCAGCTATGGGTGCTGCTACACAGCAGAATAGAACAACAGTCGGAGCTGCTACAA
                ************************************************************

Cal2            CTGTTGACGGTGCGGGATTTGACCAAACTGCCGCTCCTGCAAATCTTGCAGTTGCTCCAA
LSU             CTGTTGACGGTGCGGGATTTGACCAAACTGCCGCTCCTGCAAATCTTGCAGTTGCTCCAA
LSU-Lb          CTGTTGACGGTGCGGGATTTGACCAAACTGCCGCTCCTGCAAATCTTGCAGTTGCTCCAA
Ar3-Lb          CTGTTGACGGTGCGGGATTTGACCAAACTGCCGCTCCTGCAAATCTTGCAGTTGCTCCAA
As4-Lb          CTGTTGACGGTGCGGGATTTGACCAAACTGCCGCTCCTGCAAATCTTGCAGTTGCTCCAA
Te2-Lb          CTGTTGACGGTGCGGGATTTGACCAAACTGCCGCTCCTGCAAATCTTGCAGTTGCTCCAA
                ************************************************************

Cal2            ATGCAGTTATTACGGCTAATGCTAATAATGGTATTAATTTTAATACTCCAGCAGGTAGTT
LSU             ATGCAGTTATTACGGCTAATGCTAATAATGGTATTAATTTTAATACTCCAGCAGGTAGTT
LSU-Lb          ATGCAGTTATTACGGCTAATGCTAATAATGGTATTAATTTTAATACTCCAGCAGGTAGTT
Ar3-Lb          ATGCAGTTATTACGGCTAATGCTAATAATGGTATTAATTTTAATACTCCAGCAGGTAGTT
As4-Lb          ATGCAGTTATTACGGCTAATGCTAATAATGGTATTAATTTTAATACTCCAGCAGGTAGTT
Te2-Lb          ATGCAGTTATTACGGCTAATGCTAATAATGGTATTAATTTTAATACTCCAGCAGGTAGTT
                ************************************************************

Cal2            TTAACGGTTTGTTTTTAGATACTGCAAACAATTTAGCAGCGACAGTTAGTGAAGATACTA
LSU             TTAACGGTTTGTTTTTAGATACTGCAAACAATTTAGCAGCGACAGTTAGTGAAGATACTA
LSU-Lb          TTAACGGTTTGTTTTTAGATACTGCAAACAATTTAGCAGCGACAGTTAGTGAAGATACTA
Ar3-Lb          TTAACGGTTTGTTTTTAGATACTGCAAACAATTTAGCAGCGACAGTTAGTGAAGATACTA
As4-Lb          TTAACGGTTTGTTTTTAGATACTGCAAACAATTTAGCAGCGACAGTTAGTGAAGATACTA
Te2-Lb          TTAACGGTTTGTTTTTAGATACTGCAAACAATTTAGCAGCGACAGTTAGTGAAGATACTA
                ************************************************************
```

```
Cal2            CTTTAGGATTTATCACTAATGCTGCTAATAACGGTAACTTCTTTAATTTTACTCTTGGTG
LSU             CTTTAGGATTTATCACTAATGCTGCTAATAACGGTAACTTCTTTAATTTTACTCTTGGTG
LSU-Lb          CTTTAGGATTTATCACTAATGCTGCTAATAACGGTAACTTCTTTAATTTTACTCTTGGTG
Ar3-Lb          CTTTAGGATTTATCACTAATGCTGCTAATAACGGTAACTTCTTTAATTTTACTCTTGGTG
As4-Lb          CTTTAGGATTTATCACTAATGCTGCTAATAACGGTAACTTCTTTAATTTTACTCTTGGTG
Te2-Lb          CTTTAGGATTTATCACTAATGCTGCTAATAACGGTAACTTCTTTAATTTTACTCTTGGTG
                ************************************************************

Cal2            CCGGTAAAACTCTTACCATAACAGGACAAGGTATTACTGCTGGACAAGCTGCTGCTACAA
LSU             CCGGTAAAACTCTTACCATAACAGGACAAGGTATTACTGCTGGACAAGCTGCTGCTACAA
LSU-Lb          CCGGTAAAACTCTTACCATAACAGGACAAGGTATTACTGCTGGACAAGCTGCTGCTACAA
Ar3-Lb          CCGGTAAAACTCTTACCATAACAGGACAAGGTATTACTGCTGGACAAGCTGCTGCTACAA
As4-Lb          CCGGTAAAACTCTTACCATAACAGGACAAGGTATTACTGCTGGACAAGCTGCTGCTACAA
Te2-Lb          CCGGTAAAACTCTTACCATAACAGGACAAGGTATTACTGCTGGACAAGCTGCTGCTACAA
                ************************************************************

Cal2            AAAATGCTCAAAATGCTGTTGCACAAGTTAATGGTGGTAATGCTATTGCCAATAATGATC
LSU             AAAATGCTCAAAATGCTGTTGCACAAGTTAATGGTGGTAATGCTATTGCCAATAATGATC
LSU-Lb          AAAATGCTCAAAATGCTGTTGCACAAGTTAATGGTGGTAATGCTATTGCCAATAATGATC
Ar3-Lb          AAAATGCTCAAAATGCTGTTGCACAAGTTAATGGTGGTAATGCTATTGCCAATAATGATC
As4-Lb          AAAATGCTCAAAATGCTGTTGCACAAGTTAATGGTGGTAATGCTATTGCCAATAATGATC
Te2-Lb          AAAATGCTCAAAATGCTGTTGCACAAGTTAATGGTGGTAATGCTATTGCCAATAATGATC
                ************************************************************

Cal2            TTAGCGGTGTAGGAACAATAGATTTTGGTGCTGCGCCTTCTACATTAGTATTTAATTTAA
LSU             TTAGCGGTGTAGGAACAATAGATTTTGGTGCTGCGCCTTCTACATTAGTATTTAATTTAA
LSU-Lb          TTAGCGGTGTAGGAACAATAGATTTTGGTGCTGCGCCTTCTACATTAGTATTTAATTTAA
Ar3-Lb          TTAGCGGTGTAGGAACAATAGATTTTGGTGCTGCGCCTTCTACATTAGTATTTAATTTAA
As4-Lb          TTAGCGGTGTAGGAACAATAGATTTTGGTGCTGCGCCTTCTACATTAGTATTTAATTTAA
Te2-Lb          TTAGCGGTGTAGGAACAATAGATTTTGGTGCTGCGCCTTCTACATTAGTATTTAATTTAA
                ************************************************************

Cal2            CAAACCCTACAACTCAAAGAGCTCCTCTTATACTTGGAGATAATGCTGTAATAGCTAATG
LSU             CAAACCCTACAACTCAAAGAGCTCCTCTTATACTTGGAGATAATGCTGTAATAGCTAATG
LSU-Lb          CAAACCCTACAACTCAAAAAGCTCCTCTTATACTTGGAGATAATGCTGTAATAGCTAATG
Ar3-Lb          CAAACCCTACAACTCAAAAAGCTCCTCTTATACTTGGAGATAATGCTGTAATAGCTAATG
As4-Lb          CAAACCCTACAACTCAAAAAGCTCCTCTTATACTTGGAGATAATGCTGTAATAGCTAATG
Te2-Lb          CAAACCCTACAACTCAAAAAGCTCCTCTTATACTTGGAGATAATGCTGTAATAGCTAATG
                ****************** *****************************************

Cal2            GTGCTAACGGTACATTAAATGTTACTAATGGATTTATTCAAGTTTCAGATGAAACTTTTG
LSU             GTGCTAACGGTACATTAAATGTTACTAATGGATTTATTCAAGTTTCAGATGAAACTTTTG
LSU-Lb          GTGCTAACGGTACATTAAATGTTACTAATGGATTTATTCAAGTTTCAGATGAAACTTTTG
Ar3-Lb          GTGCTAACGGTACATTAAATGTTACTAATGGATTTATTCAAGTTTCAGATGAAACTTTTG
As4-Lb          GTGCTAACGGTACATTAAATGTTACTAATGGATTTATTCAAGTTTCAGATGAAACTTTTG
Te2-Lb          GTGCTAACGGTACATTAAATGTTACTAATGGATTTATTCAAGTTTCAGATGAAACTTTTG
                ************************************************************

Cal2            CTACTATTAAGACAATTAATATCGGTGACGGTCAAGGTTTCATTTTCAATACTGATGCTA
LSU             CTACTATTAAGACAATTAATATCGGTGACGGTCAAGGTTTCATTTTCAATACTGATGCTA
LSU-Lb          CTACTATTAAGACAATTAATATCGGTGACGGTCAAGGTTTCATTTTCAATACTGATGCTA
Ar3-Lb          CTACTATTAAGACAATTAATATCGGTGACGGTCAAGGTTTCATTTTCAATACTGATGCTA
As4-Lb          CTACTATTAAGACAATTAATATCGGTGACGGTCAAGGTTTCATTTTCAATACTGATGCTA
Te2-Lb          CTACTATTAAGACAATTAATATCGGTGACGGTCAAGGTTTCATTTTCAATACTGATGCTA
                ************************************************************

Cal2            CTGCCGGTAATGCTTTAAATTTACAAGTAGGTGGTGCTACTATTAATTTTAACGGAACAG
LSU             CTGCCGGTAATGCTTTAAATTTACAAGTAGGTGGTGCTACTATTAATTTTAACGGAACAG
LSU-Lb          CTGCCGGTAATGCTTTAAATTTACAAGTAGGTGGTGCTACTATTAATTTTAACGGAACAG
Ar3-Lb          CTGCCGGTAATGCTTTAAATTTACAAGTAGGTGGTGCTACTATTAATTTTAACGGAACAG
As4-Lb          CTGCCGGTAATGCTTTAAATTTACAAGTAGGTGGTGCTACTATTAATTTTAACGGAACAG
Te2-Lb          CTGCCGGTAATGCTTTAAATTTACAAGTAGGTGGTGCTACTATTAATTTTAACGGAACAG
                ************************************************************

Cal2            ACGGTACCGGTAGATTAGTATTATTAAGTAATGCTGCTGGTGGCGGTGCTACCGACTTTA
LSU             ACGGTACCGGTAGATTAGTATTATTAAGTAATGCTGCTGGTGGCGGTGCTACCGACTTTA
LSU-Lb          ACGGTACCGGTAGATTAGTATTATTAAGTAATGCTGCTGGTGGCGGTGCTACCGACTTTA
Ar3-Lb          ACGGTACCGGTAGATTAGTATTATTAAGTAATGCTGCTGGTGGCGGTGCTACCGACTTTA
As4-Lb          ACGGTACCGGTAGATTAGTATTATTAAGTAATGCTGCTGGTGGCGGTGCTACCGACTTTA
Te2-Lb          ACGGTACCGGTAGATTAGTATTATTAAGTAATGCTGCTGGTGGCGGTGCTACCGACTTTA
                ************************************************************
```

```
Cal2            ATGTTACAGGAAGTTTAGGCGGTAATCTAAAAGGTATTATTGAATTTAATACTACTGCAG
LSU             ATGTTACAGGAAGTTTAGGCGGTAATCTAAAAGGTATTATTGAATTTAATACTACTGCAG
LSU-Lb          ATGTTACAGGAAGTTTAGGCGGTAATCTAAAAGGTATTATTGAATTTAATACTACTGCAG
Ar3-Lb          ATGTTACAGGAAGTTTAGGCGGTAATCTAAAAGGTATTATTGAATTTAATACTACTGCAG
As4-Lb          ATGTTACAGGAAGTTTAGGCGGTAATCTAAAAGGTATTATTGAATTTAATACTACTGCAG
Te2-Lb          ATGTTACAGGAAGTTTAGGCGGTAATCTAAAAGG--------------------------
                **********************************

Cal2            TAGCAGGTCAACTTATAGCTAATGCAGGTCCTGCTAATGCAGTAATAGGTACCAATAACG
LSU             TAGCAGGTCAACTTATAGCTAATGCAGGTCCTGCTAATGCAGTAATAGGTACCAATAACG
LSU-Lb          TAGCAGGTCAACTTATAGCTAATGCAGGTCCTGCTAATGCAGTAATAGGTACCAATAACG
Ar3-Lb          TAGCAGGTCAACTTATAGCTAATGCAGGTCCTGCTAATGCAGTAATAGGTACCAATAACG
As4-Lb          TAGCAGGTCAACTTATAGCTAATGCAGGTCCTGCTAATGCAGTAATAGGTACCAATAACG
Te2-Lb          ------------------------------------------------------------


Cal2            GAGCAGGTAGAGCTGCAGGATTTGTTGTTAGCGTAGCTAACGGTAATGCAGCAACAGTCG
LSU             GAGCAGGTAGAGCTGCAGGATTTGTTGTTAGCGTAGCTAACGGTAATGCAGCAACAGTCG
LSU-Lb          GAGCAGGTAGAGCTGCAGGATTTGTTGTTAGCGTAGCTAACGGTAATGCAGCAACAGTCG
Ar3-Lb          GAGCAGGTAGAGCTGCAGGATTTGTTGTTAGCGTAGCTAACGGTAATGCAGCAACAGTCG
As4-Lb          GAGCAGGTAGAGCTGCAGGATTTGTTGTTAGCGTAGCTAACGGTAATGCAGCAACAGTCG
Te2-Lb          ------------------------------------------------------------


Cal2            CTGGACAAGTGTATGCTAAAGACATGGTTATACAAAGTACTAATGCAGGCGGACAAGTAA
LSU             CTGGACAAGTGTATGCTAAAGACATGGTTATACAAAGTACTAATGCAGGCGGACAAGTAA
LSU-Lb          CTGGACAAGTGTATGCTAAAGACATGGTTATACAAAGTACTAATGCAGGCGGACAAGTAA
Ar3-Lb          CTGGACAAGTGTATGCTAAAGACATGGTTATACAAAGTACTAATGCAGGCGGACAAGTAA
As4-Lb          CTGGACAAGTGTATGCTAAAGACATGGTTATACAAAGTACTAATGCAGGCGGACAAGTAA
Te2-Lb          ------------------------------------------------------------


Cal2            ATTTTGGACACATAGTTGATGTTGGTACGGACGGTACTACTGCATTTAAAACAGCAGCTA
LSU             ATTTTGGACACATAGTTGATGTTGGTACGGACGGTACTACTGCATTTAAAACAGCAGCTA
LSU-Lb          ATTTTGGACACATAGTTGATGTTGGTACGGACGGTACTACTGCATTTAAAACAGCAGCTA
Ar3-Lb          ATTTTGGACACATAGTTGATGTTGGTACGGACGGTACTACTGCATTTAAAACAGCAGCTA
As4-Lb          ATTTTGGACACATAGTTGATGTTGGTACGGACGGTACTACTGCATTTAAAACAGCAGCTA
Te2-Lb          ------------------------------------------------------------


Cal2            CTACAGTTGCAATAACCCAGAACTCGAACTTTGGTGCCGTTGATTTCGGTAATACTGCAT
LSU             CTACAGTTGCAATAACCCAGAACTCGAACTTTGGTGCCGTTGATTTCGGTAATACTGCAT
LSU-Lb          CTACAGTTGCAATAACCCAGAACTCGAACTTTGGTGCCGTTGATTTCGGTAATACTGCAT
Ar3-Lb          CTACAGTTGCAATAACCCAGAACTCGAACTTTGGTGCCGTTGATTTCGGTAATACTGCAT
As4-Lb          CTACAGTTGCAATAACCCAGAACTCGAACTTTGGTGCCGTTGATTTCGGTAATACTGCAT
Te2-Lb          ------------------------------------------------------------


Cal2            CACAAATTACAGTTCCTGATACTAAGGTTCTTACAGGTAACTTCACAGGTGATGCTAGCA
LSU             CACAAATTACAGTTCCTGATACTAAGGTTCTTACAGGTAACTTCACAGGTGATGCTAGCA
LSU-Lb          CACAAATTACAGTTCCTGATACTAAGGTTCTTACAGGTAACTTCACAGGTGATGCTAGCA
Ar3-Lb          CACAAATTACAGTTCCTGATACTAAGGTTCTTACAGGTAACTTCACAGGTGATGCTAGCA
As4-Lb          CACAAATTACAGTTCCTGATACTAAGGTTCTTACAGGTAACTTCACAGGTGATGCTAGCA
Te2-Lb          ------------------------------------------------------------


Cal2            ATAACGGTAATACTGCGGGTGTGATAACTTTTGCTGCTAACGGTACTTTAGCAAGCGGTA
LSU             ATAACGGTAATACTGCGGGTGTGATAACTTTTGCTGCTAACGGTACTTTAGCAAGCGGTA
LSU-Lb          ATAACGGTAATACTGCGGGTGTGATAACTTTTGCTGCTAACGGTACTTTAGCAAGCGGTA
Ar3-Lb          ATAACGGTAATACTGCGGGTGTGATAACTTTTGCTGCTAACGGTACTTTAGCAAGCGGTA
As4-Lb          ATAACGGTAATACTGCGGGTGTGATAACTTTTGCTGCTAACGGTACTTTAGCAAGCGGTA
Te2-Lb          ------------------------------------------------------------


Cal2            ATGCTGATGCAAATGTTGCGGTAACAAATAAAATTACAGCAATCGAAGCAGCAGGTGTCG
LSU             ATGCTGATGCAAATGTTGCGGTAACAAATAAAATTACAGCAATCGAAGCAGCAGGTGTCG
LSU-Lb          ATGCTGATGCAAATGTTGCGGTAACAAATAAAATTACAGCAATCGAAGCAGCAGGTGTCG
Ar3-Lb          ATGCTGATGCAAATGTTGCGGTAACAAATAAAATTACAGCAATCGAAGCAGCAGGTGTCG
As4-Lb          ATGCTGATGCAAATGTTGCGGTAACAAATAAAATTACAGCAATCGAAGCAGCAGGTGTCG
Te2-Lb          ------------------------------------------------------------
```

```
Cal2            GAGTTGTCCAATTATCAGGAACACATACTGCTGAATTACGTTTAGGAAACGCTGGTTCTC
LSU             GAGTTGTCCAATTATCAGGAACACATACTGCTGAATTACGTTTAGGAAACGCTGGTTCTC
LSU-Lb          GAGTTGTCCAATTATCAGGAACACATACTGCTGAATTACGTTTAGGAAACGCTGGTTCTC
Ar3-Lb          GAGTTGTCCAATTATCAGGAACACATACTGCTGAATTACGTTTAGGAAACGCTGGTTCTC
As4-Lb          GAGTTGTCCAATTATCAGGAACACATACTGCTGAATTACGTTTAGGAAACGCTGGTTCTC
Te2-Lb          ------------------------------------------------------------

Cal2            AGTTTAAGCTTGCTGATGGTACAATTATCAATGGTAACGTTAACCAAACTGTTCTTGTCG
LSU             AGTTTAAGCTTGCTGATGGTACAATTATCAATGGTAACGTTAACCAAACTGTTCTTGTCG
LSU-Lb          AGTTTAAGCTTGCTGATGGTACAATTATCAATGGTAACGTTAACCAAACTGTTCTTGTCG
Ar3-Lb          AGTTTAAGCTTGCTGATGGTACAATTATCAATGGTAACGTTAACCAAACTGTTCTTGTCG
As4-Lb          AGTTTAAGCTTGCTGATGGTACAATTATCAATGGTAACGTTAACCAAACTGTTCTTGTCG
Te2-Lb          ------------------------------------------------------------

Cal2            GTAATGCTGCTCTTGCAAATGGTGCTATTCAGTTAGATGGAAGTGCTACAATTACCGGTG
LSU             GTAATGCTGCTCTTGCAAATGGTGCTATTCAGTTAGATGGAAGTGCTACAATTACCGGTG
LSU-Lb          GTAATGCTGCTCTTGCAAATGGTGCTATTCAGTTAGATGGAAGTGCTACAATTACCGGTG
Ar3-Lb          GTAATGCTGCTCTTGCAAATGGTGCTATTCAGTTAGATGGAAGTGCTACAATTACCGGTG
As4-Lb          GTAATGCTGCTCTTGCAAATGGTGCTATTCAGTTAGATGGAAGTGCTACAATTACCGGTG
Te2-Lb          ------------------------------------------------------------

Cal2            ATATAGGTAACGGTGCTGGTAATGCTGCTCCGATACAAGGGATTACTTTAGCTAACGATG
LSU             ATATAGGTAACGGTGCTGGTAATGCTGCTCCGATACAAGGGATTACTTTAGCTAACGATG
LSU-Lb          ATATAGGTAACGGTGCTGGTAATGCTGCTCCGATACAAGGGATTACTTTAGCTAACGATG
Ar3-Lb          ATATAGGTAACGGTGCTGGTAATGCTGCTCCGATACAAGGGATTACTTTAGCTAACGATG
As4-Lb          ATATAGGTAACGGTGCTGGTAATGCTGCTCCGATACAAGGGATTACTTTAGCTAACGATG
Te2-Lb          ------------------------------------------------------------

Cal2            CTTCAAAAACATTAACACTTGGCGGAGCAAATATTATCGGTGCTAATGCCGGTGGAACAA
LSU             CTTCAAAAACATTAACACTTGGCGGAGCAAATATTATCGGTGCTAATGCCGGTGGAACAA
LSU-Lb          CTTCAAAAACATTAACACTTGGCGGAGCAAATATTATCGGTGCTAATGCCGGTGGAACAA
Ar3-Lb          CTTCAAAAACATTAACACTTGGCGGAGCAAATATTATCGGTGCTAATGCCGGTGGAACAA
As4-Lb          CTTCAAAAACATTAACACTTGGCGGAGCAAATATTATCGGTGCTAATGCCGGTGGAACAA
Te2-Lb          ------------------------------------------------------------

Cal2            TTGATTTCCAAGCTAACGGAGGTACTGTTAAGTTAACAAGTACTCAAAATAATATTTTAG
LSU             TTGATTTCCAAGCTAACGGAGGTACTGTTAAGTTAACAAGTACTCAAAATAATATTTTAG
LSU-Lb          TTGATTTCCAAGCTAACGGAGGTACTGTTAAGTTAACAAGTACTCAAAATAATATTTTAG
Ar3-Lb          TTGATTTCCAAGCTAACGGAGGTACTGTTAAGTTAACAAGTACTCAAAATAATATTTTAG
As4-Lb          TTGATTTCCAAGCTAACGGAGGTACTGTTAAGTTAACAAGTACTCAAAATAATATTTTAG
Te2-Lb          ------------------------------------------------------------

Cal2            TTGATTTTGATTTAGCTATCACTACTGATAAAACAGGTGTTGTTGATGCAAGTAGCCTAA
LSU             TTGATTTTGATTTAGCTATCACTACTGATAAAACAGGTGTTGTTGATGCAAGTAGCCTAA
LSU-Lb          TTGATTTTGATTTAGCTATCACTACTGATAAAACAGGTGTTGTTGATGCAAGTAGCCTAA
Ar3-Lb          TTGATTTTGATTTAGCTATCACTACTGATAAAACAGGTGTTGTTGATGCAAGTAGCCTAA
As4-Lb          TTGATTTTGATTTAGCTATCACTACTGATAAAACAGGTGTTGTTGATGCAAGTAGCCTAA
Te2-Lb          ------------------------------------------------------------

Cal2            TAAATGCTCAAACTTTAACTATTAGTGGTAATATTGGTACTATTGCAGCTAATAATAAAA
LSU             TAAATGCTCAAACTTTAACTATTAGTGGTAATATTGGTACTATTGCAGCTAATAATAAAA
LSU-Lb          TAAATGCTCAAACTTTAACTATTAGTGGTAATATTGGTACTATTGCAGCTAATAATAAAA
Ar3-Lb          TAAATGCTCAAACTTTAACTATTAGTGGTAATATTGGTACTATTGCAGCTAATAATAAAA
As4-Lb          TAAATGCTCAAACTTTAACTATTAGTGGTAATATTGGTACTATTGCAGCTAATAATAAAA
Te2-Lb          ------------------------------------------------------------

Cal2            CTCTTGGACAATTTAATATCGGCTCAAGTAAAACAGCTTTAAATAGTGGTGATGTTGCTA
LSU             CTCTTGGACAATTTAATATCGGCTCAAGTAAAACAGCTTTAAATAGTGGTGATGTTGCTA
LSU-Lb          CTCTTGGACAATTTAATATCGGCTCAAGTAAAACAGCTTTAAATAGTGGTGATGTTGCTA
Ar3-Lb          CTCTTGGACAATTTAATATCGGCTCAAGTAAAACAGCTTTAAATAGTGGTGATGTTGCTA
As4-Lb          CTCTTGGACAATTTAATATCGGCTCAAGTAAAACAGCTTTAAATAGTGGTGATGTTGCTA
Te2-Lb          ------------------------------------------------------------
```

```
Cal2           TTAATGAGTTAGTTATTGGAAATAACGGTTCAGTACAACTTGCTCACAATACTTATTTAA
LSU            TTAATGAGTTAGTTATTGGAAATAACGGTTCAGTACAACTTGCTCACAATACTTATTTAA
LSU-Lb         TTAATGAGTTAGTTATTGGAAATAACGGTTCAGTACAACTTGCTCACAATACTTATTTAA
Ar3-Lb         TTAATGAGTTAGTTATTGGAAATAACGGTTCAGTACAACTTGCTCACAATACTTATTTAA
As4-Lb         TTAATGAGTTAGTTATTGGAAATAACGGTTCAGTACAACTTGCTCACAATACTTATTTAA
Te2-Lb         ------------------------------------------------------------

Cal2           TAACAAAAACTACAAATGCTGCAAATCAAGGTAAAATAATATTTAATCCTGTTGTAAATG
LSU            TAACAAAAACTACAAATGCTGCAAATCAAGGTAAAATAATATTTAATCCTGTTGTAAATG
LSU-Lb         TAACAAAAACTACAAATGCTGCAAATCAAGGTAAAATAATATTTAATCCTGTTGTAAATG
Ar3-Lb         TAACAAAAACTACAAATGCTGCAAATCAAGGTAAAATAATATTTAATCCTGTTGTAAATG
As4-Lb         TAACAAAAACTACAAATGCTGCAAATCAAGGTAAAATAATATTTAATCCTGTTGTAAATG
Te2-Lb         ------------------------------------------------------------

Cal2           ATAATACAACTCTTGCAGCTGGCACAAATTTAGGTAGTGAAGCAAATCCTCTTGCTGAGA
LSU            ATAATACAACTCTTGCAGCTGGCACAAATTTAGGTAGTGAAGCAAATCCTCTTGCTGAGA
LSU-Lb         ATAATACAACTCTTGCAGCTGGCACAAATTTAGGTAGTGAAGCAAATCCTCTTGCTGAGA
Ar3-Lb         ATAATACAACTCTTGCAGCTGGCACAAATTTAGGTAGTGAAGCAAATCCTCTTGCTGAGA
As4-Lb         ATAATACAACTCTTGCAGCTGGCACAAATTTAGGTAGTGAAGCAAATCCTCTTGCTGAGA
Te2-Lb         ------------------------------------------------------------

Cal2           TTAATTTTGGTTCTAAAGGAGTTAATGGTGACACTATATTAAATGTTGGTCAAGGCGTAA
LSU            TTAATTTTGGTTCTAAAGGAGTTAATGGTGACACTATATTAAATGTTGGTCAAGGCGTAA
LSU-Lb         TTAATTTTGGTTCTAAAGGAGTTAATGGTGACACTATATTAAATGTTGGTCAAGGCGTAA
Ar3-Lb         TTAATTTTGGTTCTAAAGGAGTTAATGGTGACACTATATTAAATGTTGGTCAAGGCGTAA
As4-Lb         TTAATTTTGGTTCTAAAGGAGTTAATGGTGACACTATATTAAATGTTGGTCAAGGCGTAA
Te2-Lb         ------------------------------------------------------------

Cal2           ATTTATATGCTACTAATATTACTACTACCGATGCTAACGTTGGTTCGTTCAGCTTTACTG
LSU            ATTTATATGCTACTAATATTACTACTACCGATGCTAACGTTGGTTCGTTCAGCTTTACTG
LSU-Lb         ATTTATATGCTACTAATATTACTACTACCGATGCTAACGTTGGTTCGTTCAGCTTTACTG
Ar3-Lb         ATTTATATGCTACTAATATTACTACTACCGATGCTAACGTTGGTTCGTTCAGCTTTACTG
As4-Lb         ATTTATATGCTACTAATATTACTACTACCGATGCTAACGTTGGTTCGTTCAGCTTTACTG
Te2-Lb         ------------------------------------------------------------

Cal2           TCGGTGGAACAAATATAGTAAGCGGTACAGTCGGTGGACAGCAAGGTAATAAGTTTAATA
LSU            TCGGTGGAACAAATATAGTAAGCGGTACAGTCGGTGGACAGCAAGGTAATAAGTTTAATA
LSU-Lb         TCGGTGGAACAAATATAGTAAGCGGTACAGTCGGTGGACAGCAAGGTAATAAGTTTAATA
Ar3-Lb         TCGGTGGAACAAATATAGTAAGCGGTACAGTCGGTGGACAGCAAGGTAATAAGTTTAATA
As4-Lb         TCGGTGGAACAAATATAGTAAGCGGTACAGTCGGTGGACAGCAAGGTAATAAGTTTAATA
Te2-Lb         ------------------------------------------------------------

Cal2           CTGTAGAATTAGATAACGGTACTACTGCTAAGTTCTTAGGTAACGCAATATTTAACGGTG
LSU            CTGTAGAATTAGATAACGGTACTACTGCTAAGTTCTTAGGTAACGCAATATTTAACGGTG
LSU-Lb         CTGTAGAATTAGATAACGGTACTACTGCTAAGTTCTTAGGTAACGCAATATTTAACGGTG
Ar3-Lb         CTGTAGAATTAGATAACGGTACTACTGCTAAGTTCTTAGGTAACGCAATATTTAACGGTG
As4-Lb         CTGTAGAATTAGATAACGGTACTACTGCTAAGTTCTTAGGTAACGCAATATTTAACGGTG
Te2-Lb         ------------------------------------------------------------

Cal2           AAACTACAATTGAAGCTAACTCTATCTTACAAATCGGTGGTAACTATACTGCAGATAAGG
LSU            AAACTACAATTGAAGCTAACTCTATCTTACAAATCGGTGGTAACTATACTGCAGATAAGG
LSU-Lb         AAACTACAATTGAAGCTAACTCTATCTTACAAATCGGTGGTAACTATACTGCAGATAAGG
Ar3-Lb         AAACTACAATTGAAGCTAACTCTATCTTACAAATCGGTGGTAACTATACTGCAGATAAGG
As4-Lb         AAACTACAATTGAAGCTAACTCTATCTTACAAATCGGTGGTAACTATACTGCAGATAAGG
Te2-Lb         ------------------------------------------------------------

Cal2           TTGAATCTGCTGATGGTACCGGTATAGTAGAATTTGTTAACACCACTCCTATTACCGTAA
LSU            TTGAATCTGCTGATGGTACCGGTATAGTAGAATTTGTTAACACCACTCCTATTACCGTAA
LSU-Lb         TTGAATCTGCTGATGGTACCGGTATAGTAGAATTTGTTAACACCACTCCTATTACCGTAA
Ar3-Lb         TTGAATCTGCTGATGGTACCGGTATAGTAGAATTTGTTAACACCACTCCTATTACCGTAA
As4-Lb         TTGAATCTGCTGATGGTACCGGTATAGTAGAATTTGTTAACACCACTCCTATTACCGTAA
Te2-Lb         ------------------------------------------------------------
```

```
Cal2            CATTAAACAAACAAGCTGGACCTGTTGATGATTTAAAACAAATAACAGTTTCCGGTCGCG
LSU             CATTAAACAAACAAGCTGGACCTGTTGATGATTTAAAACAAATAACAGTTTCCGGTCGCG
LSU-Lb          CATTAAACAAACAAGCTGGACCTGTTGATGATTTAAAACAAATAACAGTTTCCGGTCGCG
Ar3-Lb          CATTAAACAAACAAGCTGGACCTGTTGATGATTTAAAACAAATAACAGTTTCCGGTCGCG
As4-Lb          CATTAAACAAACAAGCTGGACCTGTTGATGATTTAAAACAAATAACAGTTTCCGGTCGCG
Te2-Lb          ------------------------------------------------------------

Cal2            GTAACGTAGTGATTAATGAAATAGGTAATGCAGGTAATGACCATGGTGCGGCGACTGATA
LSU             GTAACGTAGTGATTAATGAAATAGGTAATGCAGGTAATGACCATGGTGCGGCGACTGATA
LSU-Lb          GTAACGTAGTGATTAATGAAATAGGTAATGCAGGTAATGACCATGGTGCGGCGACTGATA
Ar3-Lb          GTAACGTAGTGATTAATGAAATAGGTAATGCAGGTAATGACCATGGTGCGGCGACTGATA
As4-Lb          GTAACGTAGTGATTAATGAAATAGGTAATGCAGGTAATGACCATGGTGCGGCGACTGATA
Te2-Lb          ------------------------------------------------------------

Cal2            CAATTTCTTTTGAAAATGTAAGTTTAGGTGCAGCTTTATTCTTACCTAACGGGATCCCAT
LSU             CAATTTCTTTTGAAAATGTAAGTTTAGGTGCAGCTTTATTCTTACCTAACGGGATCCCAT
LSU-Lb          CAATTTCTTTTGAAAATGTAAGTTTAGGTGCAGCTTTATTCTTACCTAACGGGATCCCAT
Ar3-Lb          CAATTTCTTTTGAAAATGTAAGTTTAGGTGCAGCTTTATTCTTACCTAACGGGATCCCAT
As4-Lb          CAATTTCTTTTGAAAATGTAAGTTTAGGTGCAGCTTTATTCTTACCTAACGGGATCCCAT
Te2-Lb          ------------------------------------------------------------

Cal2            TAGACGGTTTAACAATTAAAAGTACAGTAGGTAATGAAACAGCTACAGGTAATTTTGATG
LSU             TAGACGGTTTAACAATTAAAAGTACAGTAGGTAATGAAACAGCTACAGGTAATTTTGATG
LSU-Lb          TAGACGGTTTAACAATTAAAAGTACAGTAGGTAATGAAACAGCTACAGGTAATTTTGATG
Ar3-Lb          TAGACGGTTTAACAATTAAAAGTACAGTAGGTAATGAAACAGCTACAGGTAATTTTGATG
As4-Lb          TAGACGGTTTAACAATTAAAAGTACAGTAGGTAATGAAACAGCTACAGGTAATTTTGATG
Te2-Lb          ------------------------------------------------------------

Cal2            TTCCTAGACTGATTGTTTCAGGTGTTGATAGCGTAATCGCTGACGGTCAAGCAATCGGTG
LSU             TTCCTAGACTGATTGTTTCAGGTGTTGATAGCGTAATCGCTGACGGTCAAGCAATCGGTG
LSU-Lb          TTCCTAGACTGATTGTTTCAGGTGTTGATAGCGTAATCGCTGACGGTCAAGCAATCGGTG
Ar3-Lb          TTCCTAGACTGATTGTTTCAGGTGTTGATAGCGTAATCGCTGACGGTCAAGCAATCGGTG
As4-Lb          TTCCTAGACTGATTGTTTCAGGTGTTGATAGCGTAATCGCTGACGGTCAAGCAATCGGTG
Te2-Lb          ------------------------------------------------------------

Cal2            ATCAGGATAATATTGTAGGTCTTGGTCTTGGAAGTGATAACAGTATTACTGTTAATGCTA
LSU             ATCAGGATAATATTGTAGGTCTTGGTCTTGGAAGTGATAACAGTATTACTGTTAATGCTA
LSU-Lb          ATCAGGATAATATTGTAGGTCTTGGTCTTGGAAGTGATAACAGTATTACTGTTAATGCTA
Ar3-Lb          ATCAGGATAATATTGTAGGTCTTGGTCTTGGAAGTGATAACAGTATTACTGTTAATGCTA
As4-Lb          ATCAGGATAATATTGTAGGTCTTGGTCTTGGAAGTGATAACAGTATTACTGTTAATGCTA
Te2-Lb          ------------------------------------------------------------

Cal2            CTAAATTATATGCAGGTATCGGTAGCGTAAACAATAATCAAGGTACTGTCACACTTAGCG
LSU             CTAAATTATATGCAGGTATCGGTAGCGTAAACAATAATCAAGGTACTGTCACACTTAGCG
LSU-Lb          CTAAATTATATGCAGGTATCGGTAGCGTAAACAATAATCAAGGTACTGTCACACTTAGCG
Ar3-Lb          CTAAATTATATGCAGGTATCGGTAGCGTAAACAATAATCAAGGTACTGTCACACTTAGCG
As4-Lb          CTAAATTATATGCAGGTATCGGTAGCGTAAACAATAATCAAGGTACTGTCACACTTAGCG
Te2-Lb          ------------------------------------------------------------

Cal2            GCGGTATTCCTAACACCCCAGGTACAATTTACGGTTTAGGTATAGAGAACGGTAGTCCGA
LSU             GCGGTATTCCTAACACCCCAGGTACAATTTACGGTTTAGGTATAGAGAACGGTAGTCCGA
LSU-Lb          GCGGTATTCCTAACACCCCAGGTACAATTTACGGTTTAGGTATAGAGAACGGTAGTCCGA
Ar3-Lb          GCGGTATTCCTAACACCCCAGGTACAATTTACGGTTTAGGTATAGAGAACGGTAGTCCGA
As4-Lb          GCGGTATTCCTAACACCCCAGGTACAATTTACGGTTTAGGTATAGAGAACGGTAGTCCGA
Te2-Lb          ------------------------------------------------------------

Cal2            AGTTAAAGCAAGTAACGTTTACTACAGACTATAACAACTTAGGTAGTATTATCGCAACTA
LSU             AGTTAAAGCAAGTAACGTTTACTACAGACTATAACAACTTAGGTAGTATTATCGCAACTA
LSU-Lb          AGTTAAAGCAAGTAACGTTTACTACAGACTATAACAACTTAGGTAGTATTATCGCAACTA
Ar3-Lb          AGTTAAAGCAAGTAACGTTTACTACAGACTATAACAACTTAGGTAGTATTATCGCAACTA
As4-Lb          AGTTAAAGCAAGTAACGTTTACTACAGACTATAACAACTTAGGTAGTATTATCGCAACTA
Te2-Lb          ------------------------------------------------------------
```

```
Cal2               ACGCAACAATTAATGACGGTGTAACTGTTACTACAGGTGGTGTAGCCGGAACAGATTTCG
LSU                ACGCAACAATTAATGACGGTGTAACTGTTACTACAGGTGGTGTAGCCGGAACAGATTTCG
LSU-Lb             ACGCAACAATTAATGACGGTGTAACTGTTACTACAGGTGGTGTAGCCGGAACAGATTTCG
Ar3-Lb             ACGCAACAATTAATGACGGTGTAACTGTTACTACAGGTGGTGTAGCCGGAACAGATTTCG
As4-Lb             ACGCAACAATTAATGACGGTGTAACTGTTACTACAGGTGGTGTAGCCGGAACAGATTTCG
Te2-Lb             ------------------------------------------------------------

Cal2               ACGGTAAAATTACCCTTGGAAGTGTTAATGGTAACGCTAATGTAAGATTTGTTGACGGTA
LSU                ACGGTAAAATTACCCTTGGAAGTGTTAATGGTAACGCTAATGTAAGATTTGTTGACGGTA
LSU-Lb             ACGGTAAAATTACCCTTGGAAGTGTTAATGGTAACGCTAATGTAAGATTTGTTGACGGTA
Ar3-Lb             ACGGTAAAATTACCCTTGGAAGTGTTAATGGTAACGCTAATGTAAGATTTGTTGACGGTA
As4-Lb             ACGGTAAAATTACCCTTGGAAGTGTTAATGGTAACGCTAATGTAAGATTTGTTGACGGTA
Te2-Lb             ------------------------------------------------------------

Cal2               CATTTTCTGATTCTACAAGTATGATTGTTACTACTAAAGCTAATAACGGTACTGTAACTT
LSU                CATTTTCTGATTCTACAAGTATGATTGTTACTACTAAAGCTAATAACGGTACTGTAACTT
LSU-Lb             CATTTTCTGATTCTACAAGTATGATTGTTACTACTAAAGCTAATAACGGTACTGTAACTT
Ar3-Lb             CATTTTCTGATTCTACAAGTATGATTGTTACTACTAAAGCTAATAACGGTACTGTAACTT
As4-Lb             CATTTTCTGATTCTACAAGTATGATTGTTACTACTAAAGCTAATAACGGTACTGTAACTT
Te2-Lb             ------------------------------------------------------------

Cal2               ACTTAGGTAGTGCATTAGTCGGTAATATAGGTAGTTCAGATACTCCTGTAGCTTCTGTTA
LSU                ACTTAGGTAGTGCATTAGTCGGTAATATAGGTAGTTCAGATACTCCTGTAGCTTCTGTTA
LSU-Lb             ACTTAGGTAGTGCATTAGTCGGTAATATAGGTAGTTCAGATACTCCTGTAGCTTCTGTTA
Ar3-Lb             ACTTAGGTAGTGCATTAGTCGGTAATATAGGTAGTTCAGATACTCCTGTAGCTTCTGTTA
As4-Lb             ACTTAGGTAGTGCATTAGTCGGTAATATAGGTAGTTCAGATACTCCTGTAGCTTCTGTTA
Te2-Lb             ------------------------------------------------------------

Cal2               AATTTATAGGTAGTGATGATGGTGCAGGATTACAAGGAAATATTTATTCACAAGTCACAG
LSU                AATTTATAGGTAGTGATGATGGTGCAGGATTACAAGGAAATATTTATTCACAAGTCACAG
LSU-Lb             AATTTATAGGTAGTGATGATGGTGCAGGATTACAAGGAAATATTTATTCACAAGTCACAG
Ar3-Lb             AATTTATAGGTAGTGATGATGGTGCAGGATTACAAGGAAATATTTATTCACAAGTCACAG
As4-Lb             AATTTATAGGTAGTGATGATGGTGCAGGATTACAAGGAAATATTTATTCACAAGTCACAG
Te2-Lb             ------------------------------------------------------------

Cal2               ACTTTGGTACTTATGACTTAAGTGTTTTAAATTCTAACGTAATTTTAGGCGGCGGTACTA
LSU                ACTTTGGTACTTATGACTTAAGTGTTTTAAATTCTAACGTAATTTTAGGCGGCGGTACTA
LSU-Lb             ACTTTGGTACTTATGACTTAAGTGTTTTAAATTCTAACGTAATTTTAGGCGGCGGTACTA
Ar3-Lb             ACTTTGGTACTTATGACTTAAGTGTTTTAAATTCTAACGTAATTTTAGGCGGCGGTACTA
As4-Lb             ACTTTGGTACTTATGACTTAAGTGTTTTAAATTCTAACGTAATTTTAGGCGGCGGTACTA
Te2-Lb             ------------------------------------------------------------

Cal2               CTGCTATTAACGGTGAAATCGATCTTCTTACAAATACCTTAACATTTGCAAGCGGTACTT
LSU                CTGCTATTAACGGTGAAATCGATCTTCTTACAAATACCTTAACATTTGCAAGCGGTACTT
LSU-Lb             CTGCTATTAACGGTGAAATCGATCTTCTTACAAATACCTTAACATTTGCAAGCGGTACTT
Ar3-Lb             CTGCTATTAACGGTGAAATCGATCTTCTTACAAATACCTTAACATTTGCAAGCGGTACTT
As4-Lb             CTGCTATTAACGGTGAAATCGATCTTCTTACAAATACCTTAACATTTGCAAGCGGTACTT
Te2-Lb             ------------------------------------------------------------

Cal2               CAACATGGGGAAGCAATACTTCTATTGAAACTACTTTAACAGTAGCAAACGGTAATATAG
LSU                CAACATGGGGAAGCAATACTTCTATTGAAACTACTTTAACAGTAGCAAACGGTAATATAG
LSU-Lb             CAACATGGGGAAGCAATACTTCTATTGAAACTACTTTAACAGTAGCAAACGGTAATATAG
Ar3-Lb             CAACATGGGGAAGCAATACTTCTATTGAAACTACTTTAACAGTAGCAAACGGTAATATAG
As4-Lb             CAACATGGGGAAGCAATACTTCTATTGAAACTACTTTAACAGTAGCAAACGGTAATATAG
Te2-Lb             ------------------------------------------------------------

Cal2               GTCACATCGTTATTGCAGAAAATGCTCAAGTTAATGCAACAACTACGGGAACAACAACTA
LSU                GTCACATCGTTATTGCAGAAAATGCTCAAGTTAATGCAACAACTACGGGAACAACAACTA
LSU-Lb             GTCACATCGTTATTGCAGAAAATGCTCAAGTTAATGCAACAACTACGGGAACAACAACTA
Ar3-Lb             GTCACATCGTTATTGCAGAAAATGCTCAAGTTAATGCAACAACTACGGGAACAACAACTA
As4-Lb             GTCACATCGTTATTGCAGAAAATGCTCAAGTTAATGCAACAACTACGGGAACAACAACTA
Te2-Lb             ------------------------------------------------------------
```

```
Cal2          TTAACGTACAAGATAATGCCAATGCAAATTTCAGCGATACGCAAACTTATACTTTAATCC
LSU           TTAACGTACAAGATAATGCCAATGCAAATTTCAGCGATACGCAAACTTATACTTTAATCC
LSU-Lb        TTAACGTACAAGATAATGCCAATGCAAATTTCAGCGATACGCAAACTTATACTTTAATCC
Ar3-Lb        TTAACGTACAAGATAATGCCAATGCAAATTTCAGCGATACGCAAACTTATACTTTAATCC
As4-Lb        TTAACGTACAAGATAATGCCAATGCAAATTTCAGCGATACGCAAACTTATACTTTAATCC
Te2-Lb        ------------------------------------------------------------

Cal2          AAGGTGGTGCTAGATTTAACGGTACTTTAGGAGGTCCTAACTTTGCTGTAACCGGAAGTA
LSU           AAGGTGGTGCTAGATTTAACGGTACTTTAGGAGGTCCTAACTTTGCTGTAACCGGAAGTA
LSU-Lb        AAGGTGGTGCTAGATTTAACGGTACTTTAGGAGGTCCTAACTTTGCTGTAACCGGAAGTA
Ar3-Lb        AAGGTGGTGCTAGATTTAACGGTACTTTAGGAGGTCCTAACTTTGCTGTAACCGGAAGTA
As4-Lb        AAGGTGGTGCTAGATTTAACGGTACTTTAGGAGGTCCTAACTTTGCTGTAACCGGAAGTA
Te2-Lb        ------------------------------------------------------------

Cal2          ATCGTTTCGTTAATTACGGTTTAATACGTGCTGCTAACCAAGATTATGTAATAACACGTA
LSU           ATCGTTTCGTTAATTACGGTTTAATACGTGCTGCTAACCAAGATTATGTAATAACACGTA
LSU-Lb        ATCGTTTCGTTAATTACGGTTTAATACGTGCTGCTAACCAAGATTATGTAATAACACGTA
Ar3-Lb        ATCGTTTCGTTAATTACGGCTTAATACGTGCTGCTAACCAAGATTATGTAATAACACGTA
As4-Lb        ATCGTTTCGTTAATTACGGCTTAATACGTGCTGCTAACCAAGATTATGTAATAACACGTA
Te2-Lb        ------------------------------------------------------------

Cal2          CTAACAATGCAGCAAACGTAGTTACTAATGATATCGCAAATAGCCCATTTGCAAGTGCAC
LSU           CTAACAATGCAGCAAACGTAGTTACTAATGATATCGCAAATAGCCCATTTGCAAGTGCAC
LSU-Lb        CTAACAATGCAGCAAACGTAGTTACTAATGATATCGCAAATAGCCCATTTGCAAGTGCAC
Ar3-Lb        CTAACAATGCAGCAAACGTAGTTACTAATGATATCGCAAATAGCCCATTTGCAAGTGCAC
As4-Lb        CTAACAATGCAGCAAACGTAGTTACTAATGATATCGCAAATAGCCCATTTGCAAGTGCAC
Te2-Lb        ------------------------------------------------------------

Cal2          CGGGTGTAGGTCAGAACGTTACAACATTTGTAAATTCAACTAATACTGCGGCGTATAATA
LSU           CGGGTGTAGGTCAGAACGTTACAACATTTGTAAATTCAACTAATACTGCGGCGTATAATA
LSU-Lb        CGGGTGTAGGTCAGAACGTTACAACATTTGTAAATTCAACTAATACTGCGGCGTATAATA
Ar3-Lb        CGGGTGTAGGTCAGAACGTTACAACATTTGTAAATTCAACTAATACTGCGGCGTATAATA
As4-Lb        CGGGTGTAGGTCAGAACGTTACAACATTTGTAAATTCAACTAATACTGCGGCGTATAATA
Te2-Lb        ------------------------------------------------------------

Cal2          ATCTTCTTTTAGCTAAAAATAGTGCTGATTCTGCTAACTTTGTTGGAGCTATTACTACCG
LSU           ATCTTCTTTTAGCTAAAAATAGTGCTGATTCTGCTAACTTTGTTGGAGCTATTACTACCG
LSU-Lb        ATCTTCTTTTAGCTAAAAATAGTGCTGATTCTGCTAACTTTGTTGGAGCTATTACTACCG
Ar3-Lb        ATCTTCTTTTAGCTAAAAATAGTGCTGATTCTGCTAACTTTGTTGGAGCTATTACTACCG
As4-Lb        ATCTTCTTTTAGCTAAAAATAGTGCTGATTCTGCTAACTTTGTTGGAGCTATTACTACCG
Te2-Lb        ------------------------------------------------------------

Cal2          ATACAAGTGCAGCCATAACTAATGCACAATTAGATGTAGCAAAAGACATCCAAGCTCAAC
LSU           ATACAAGTGCAGCCATAACTAATGCACAATTAGATGTAGCAAAAGACATCCAAGCTCAAC
LSU-Lb        ATACAAGTGCAGCCATAACTAATGCACAATTAGATGTAGCAAAAGACATCCAAGCTCAAC
Ar3-Lb        ATACAAGTGCAGCCATAACTAATGCACAATTAGATGTAGCAAAAGACATCCAAGCTCAAC
As4-Lb        ATACAAGTGCAGCCATAACTAATGCACAATTAGATGTAGCAAAAGACATCCAAGCTCAAC
Te2-Lb        ------------------------------------------------------------

Cal2          TTGGTAACAGATTAGGTGCTCTTAGATATTTAGGTACTCCTGAAACTGCTGAAATGGCTG
LSU           TTGGTAACAGATTAGGTGCTCTTAGATATTTAGGTACTCCTGAAACTGCTGAAATGGCTG
LSU-Lb        TTGGTAACAGATTAGGTGCTCTTAGATATTTAGGTACTCCTGAAACTGCTGAAATGGCTG
Ar3-Lb        TTGGTAACAGATTAGGTGCTCTTAGATATTTAGGTACTCCTGAAACTGCTGAAATGGCTG
As4-Lb        TTGGTAACAGATTAGGTGCTCTTAGATATTTAGGTACTCCTGAAACTGCTGAAATGGCTG
Te2-Lb        ------------------------------------------------------------

Cal2          GACCTGAAGCTGGAGCAATACCGGCTGCGGTTGCTGCAGGTGACGAAGCTGTTGACAATG
LSU           GACCTGAAGCTGGAGCAATACCGGCTGCGGTTGCTGCAGGTGACGAAGCTGTTGACAATG
LSU-Lb        GACCTGAAGCTGGAGCAATACCGGCTGCGGTTGCTGCAGGTGACGAAGCTGTTGACAATG
Ar3-Lb        GACCTGAAGCTGGAGCAATACCGGCTGCGGTTGCTGCAGGTGACGAAGCTGTTGACAATG
As4-Lb        GACCTGAAGCTGGAGCAATACCGGCTGCGGTTGCTGCAGGTGACGAAGCTGTTGACAATG
Te2-Lb        ------------------------------------------------------------
```

```
Cal2            TAGCTTACGGTATATGGGCAAAACCTTTCTATACTGATGCACATCAAAGTAAGAAAGGCG
LSU             TAGCTTACGGTATATGGGCAAAACCTTTCTATACTGATGCACATCAAAGTAAGAAAGGCG
LSU-Lb          TAGCTTACGGTATATGGGCAAAACCTTTCTATACTGATGCACATCAAAGTAAGAAAGGCG
Ar3-Lb          TAGCTTACGGTATATGGGCAAAACCTTTCTATACTGATGCACATCAAAGTAAGAAAGGCG
As4-Lb          TAGCTTACGGTATATGGGCAAAACCTTTCTATACTGATGCACATCAAAGTAAGAAAGGCG
Te2-Lb          ------------------------------------------------------------

Cal2            GTTTAGCTGGTTATAAAGCTAAAACCACCGGTGTCGTAATAGGTTTAGACACGCTAGCTA
LSU             GTTTAGCTGGTTATAAAGCTAAAACCACCGGTGTCGTAATAGGTTTAGACACGCTAGCTA
LSU-Lb          GTTTAGCTGGTTATAAAGCTAAAACCACCGGTGTCGTAATAGGTTTAGACACGCTAGCTA
Ar3-Lb          GTTTAGCTGGTTATAAAGCTAAAACCACCGGTGTCGTAATAGGTTTAGACACGCTAGCTA
As4-Lb          GTTTAGCTGGTTATAAAGCTAAAACCACCGGTGTCGTAATAGGTTTAGACACGCTAGCTA
Te2-Lb          ------------------------------------------------------------

Cal2            ACGATAACTTAATGATCGGTGCTGCTATCGGTATTACTAAAACCGATATAAAACACCAGG
LSU             ACGATAACTTAATGATCGGTGCTGCTATCGGTATTACTAAAACCGATATAAAACACCAGG
LSU-Lb          ACGATAACTTAATGATCGGTGCTGCTATCGGTATTACTAAAACCGATATAAAACACCAGG
Ar3-Lb          ACGATAACTTAATGATCGGTGCTGCTACCGGTATTACTAAAACCGATATAAAACACCAGG
As4-Lb          ACGATAACTTAATGATCGGTGCTGCTACCGGTATTACTAAAACCGATATAAAACACCAGG
Te2-Lb          ------------------------------------------------------------

Cal2            ATTATAAGAAAGGTGATAAAACCGACGTTAACGGTTTCACATTCTCTCTATATGGTGCCC
LSU             ATTATAAGAAAGGTGATAAAACCGACGTTAACGGTTTCACATTCTCTCTATATGGTGCCC
LSU-Lb          ATTATAAGAAAGGTGATAAAACCGACGTTAACGGTTTCACATTCTCTCTATATGGTGCCC
Ar3-Lb          ATTATAAGAAAGGTGATAAAACCGACGTTAACGGTTTCACATTCTCTCTATATGGTGCCC
As4-Lb          ATTATAAGAAAGGTGATAAAACCGACGTTAACGGTTTCACATTCTCTCTATATGGTGCCC
Te2-Lb          ------------------------------------------------------------

Cal2            AGCAGCTTGTTGAGAACTTCTTTGCTCAAGGTAGTGCAATATTTAGCTTAAACCAAGTGA
LSU             AGCAGCTTGTTGAGAACTTCTTTGCTCAAGGTAGTGCAATATTTAGCTTAAACCAAGTGA
LSU-Lb          AGCAGCTTGTTGAGAACTTCTTTGCTCAAGGTAGTGCAATATTTAGCTTAAACCAAGTGA
Ar3-Lb          AGCAGCTTGTTGAGAACTTCTTTGCTCAAGGTAGTGCAATATTTAGCTTAAACCAAGTGA
As4-Lb          AGCAGCTTGTTGAGAACTTCTTTGCTCAAGGTAGTGCAATATTTAGCTTAAACCAAGTGA
Te2-Lb          ------------------------------------------------------------

Cal2            AGAACAAAAGTCAGCGTTACTTCTTCGATGCTAACGGTAAGATGAATAAGCAAATTGCTG
LSU             AGAACAAAAGTCAGCGTTACTTCTTCGATGCTAACGGTAAGATGAATAAGCAAATTGCTG
LSU-Lb          AGAACAAAAGTCAGCGTTACTTCTTCGATGCTAACGGTAAGATGAATAAGCAAATTGCTG
Ar3-Lb          AGAACAAAAGTCAGCGTTACTTCTTCGATGCTAACGGTAAGATGAATAAGCAAATTGCTG
As4-Lb          AGAACAAAAGTCAGCGTTACTTCTTCGATGCTAACGGTAAGATGAATAAGCAAATTGCTG
Te2-Lb          ------------------------------------------------------------

Cal2            CCGGTAATTACGATAACATGACATTCGGTGGTAACTTAATGGTCGGTTATGATTACAATG
LSU             CCGGTAATTACGATAACATGACATTCGGTGGTAACTTAATGGTCGGTTATGATTACAATG
LSU-Lb          CCGGTAATTACGATAACATGACATTCGGTGGTAACTTAATGGTCGGTTATGATTACAATG
Ar3-Lb          CCGGTAATTACGATAACATGACATTCGGTGGTAACTTAATGGTCGGTTATGATTACAATG
As4-Lb          CCGGTAATTACGATAACATGACATTCGGTGGTAACTTAATGGTCGGTTATGATTACAATG
Te2-Lb          ------------------------------------------------------------

Cal2            CAATGCAAGGTGTATTAGTAACTCCAATGGCAGGACTTAGCTACTTAAAGTCTTCTGACG
LSU             CAATGCAAGGTGTATTAGTAACTCCAATGGCAGGACTTAGCTACTTAAAGTCTTCTGACG
LSU-Lb          CAATGCAAGGTGTATTAGTAACTCCAATGGCAGGACTTAGCTACTTAAAGTCTTCTGACG
Ar3-Lb          CAATGCAAGGTGTATTAGTAACTCCAATGGCAGGACTTAGCTACTTAAAGTCTTCTGACG
As4-Lb          CAATGCAAGGTGTATTAGTAACTCCAATGGCAGGACTTAGCTACTTAAAGTCTTCTGACG
Te2-Lb          ------------------------------------------------------------

Cal2            AAAACTACAAAGAAACCGGTACAACAGTTGCAAACAAGCAAGTTAACAGCAAGTTTAGCG
LSU             AAAACTACAAAGAAACCGGTACAACAGTTGCAAACAAGCAAGTTAACAGCAAGTTTAGCG
LSU-Lb          AAAACTACAAAGAAACCGGTACAACAGTTGCAAACAAGCAAGTTAACAGCAAGTTTAGCG
Ar3-Lb          AAAACTACAAAGAAACCGGTACAACAGTTGCAAACAAGCAAGTTAACAGCAAGTTTAGCG
As4-Lb          AAAACTACAAAGAAACCGGTACAACAGTTGCAAACAAGCAAGTTAACAGCAAGTTTAGCG
Te2-Lb          ------------------------------------------------------------
```

```
Cal2            ATAGAACTGATTTAATAGTAGGTGTTAAAGTAGCCGGTGGTACTATGAATATAACTGATC
LSU             ATAGAACTGATTTAATAGTAGGTGTTAAAGTAGCCGGTGGTACTATGAATATAACTGATC
LSU-Lb          ATAGAACTGATTTAATAGTAGGTGTTAAAGTAGCCGGTGGTACTATGAATATAACTGATC
Ar3-Lb          ATAGAACTGATTTAATAGTAGGTGTTAAAGTAGCCGGTGGTACTATGAATATAACTGATC
As4-Lb          ATAGAACTGATTTA----------------------------------------------
Te2-Lb          ------------------------------------------------------------

Cal2            TTGCGGTATACCCGGAAGCTCACGCTTTTGTAGTTCACAAAGTAAATGGTAGATTATCTA
LSU             TTGCGGTATACCCGGAAGCTCACGCTTTTGTAGTTCACAAAGTAAATGGTAGATTATCTA
LSU-Lb          TTGCGGTATACCCGGAAGCTCACGCTTTTGTAGTTCACAAAGTAAATGGTAGATTATCTA
Ar3-Lb          TTGCGGTATACCCGGAAGCTCACGCTTTTGTAGTTCACAAAGTAAATGGTAGATTATCTA
As4-Lb          ------------------------------------------------------------
Te2-Lb          ------------------------------------------------------------

Cal2            AAACTCAGTCTCAGTTAGATGGACAAGTTACTCCGTTCATCAGCCAGCCTGACAGAACTG
LSU             AAACTCAGTCTCAGTTAGATGGACAAGTTACTCCGTTCATCAGCCAGCCTGACAGAACTG
LSU-Lb          AAACTCAGTCTCAGTTAGATGGACAAGTTACTCCGTTCATCAGCCAGCCTGACAGAACTG
Ar3-Lb          AAACTCAGTCTCAGTTAGATGGACAAGTTACTCCGTTCATCAGCCAGCCTGACAGAACTG
As4-Lb          ------------------------------------------------------------
Te2-Lb          ------------------------------------------------------------

Cal2            CTAAAACATCTTACAATATAGGTTTAAGTGCAAGCATAAGACCTGATGCTAAGATGGAAT
LSU             CTAAAACATCTTACAATATAGGTTTAAGTGCAAGCATAAGACCTGATGCTAAGATGGAAT
LSU-Lb          CTAAAACATCTTACAATATAGGTTTAAGTGCAAGCATAAGACCTGATGCTAAGATGGAAT
Ar3-Lb          CTAAAACATCTTACAATATAGGTTTAAGTGCAAGCATAAGACCTGATGCTAAGATGGAAT
As4-Lb          ------------------------------------------------------------
Te2-Lb          ------------------------------------------------------------

Cal2            ACGGAATCGGTTATGATTTCAATGCTGCAAGTAAGTATACTGCACATCAAGGTACTTTAA
LSU             ACGGAATCGGTTATGATTTCAATGCTGCAAGTAAGTATACTGCACATCAAGGTACTTTAA
LSU-Lb          ACGGAATCGGTTATGATTTCAATGCTGCAAGTAAGTATACTGCACATCAAGGTACTTTAA
Ar3-Lb          ACGGAATCGGTTATGATTTCAATGCTGCAAGTAAGTATACTGCACATCAAGGTACTTTAA
As4-Lb          ------------------------------------------------------------
Te2-Lb          ------------------------------------------------------------

Cal2            AAGTACGTGTAAACTTCTAA
LSU             AAGTACGTGTAAACTTCTAA
LSU-Lb          AAGTACGTGTAAACTTCTAA
Ar3-Lb          AAGTCCGTGTAAACTTCTAA
As4-Lb          --------------------
Te2-Lb          --------------------
```

Literature Cited

Edgar RC 2004. MUSCLE: multiple sequence alignment with high accuracy and high throughput. Nucleic Acids Res 32: 1792-1797. doi: 10.1093/nar/gkh340
32/5/1792 [pii]

Supplementary fig. S5. Synteny analysis of four *R. felis* pRF plasmids.

Alignment was performed with the complete plasmid pRF sequences of *Rickettsia felis* str. URRWXCal2 (NC_007109), *R. felis* str. “AUS-Lb” (GQ329881) and *R. felis* str. LSU, plus eight stitched contigs from the *R. felis* str. LSU-Lb assembly. Genome sequence alignments were performed using Mauve v.2.3.1, utilizing the “progressiveMauve” option (Darling, et al. 2010). NOTE: some pRF genes of *R. felis* str. LSU-Lb were identified subsequently on small scaffolds and later included in the comparative analyses (see text for more details).

Literature Cited

Darling AE, Mau B, Perna NT 2010. progressiveMauve: multiple genome alignment with gene gain, loss and rearrangement. PLos One 5: e11147. doi: 10.1371/journal.pone.0011147

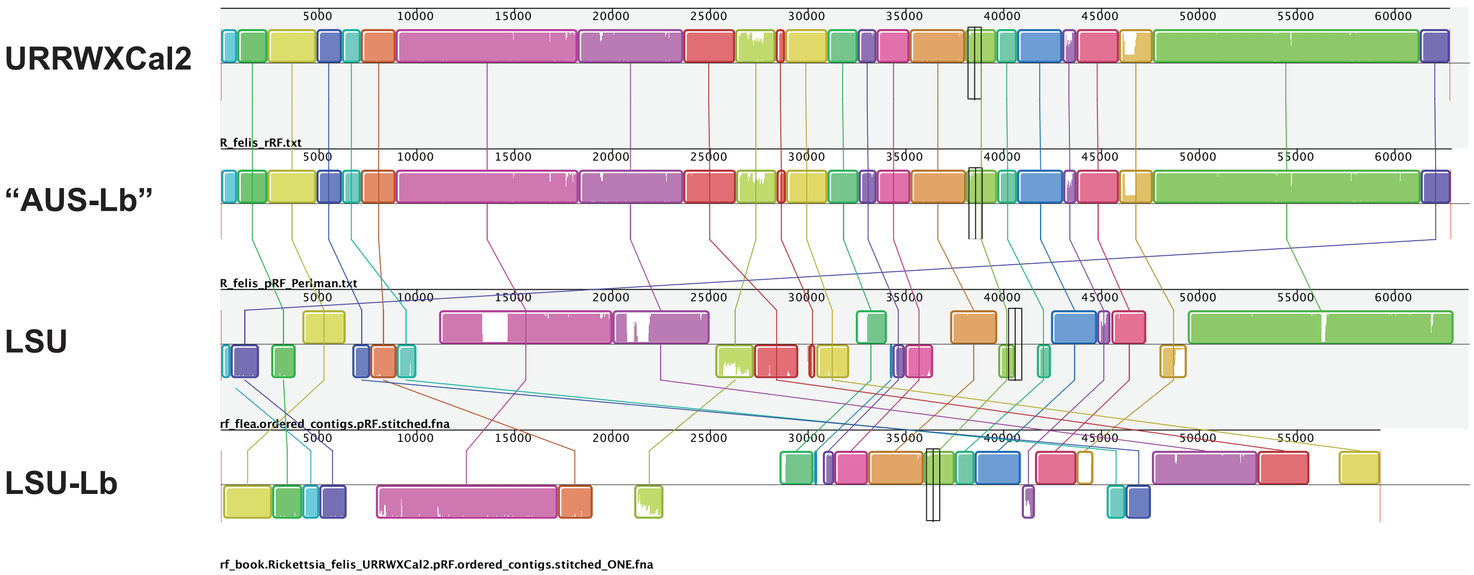

URRWXCal2
5000 10000 15000 20000 25000 30000 35000 40000 45000 50000 55000 60000
R_felis_rRF.txt
“AUS-Lb”
5000 10000 15000 20000 25000 30000 35000 40000 45000 50000 55000 60000
R_felis_pRF_Perlman.txt
LSU
5000 10000 15000 20000 25000 30000 35000 40000 45000 50000 55000 60000
rf_flea.ordered_contigs.pRF.stitched.fna
LSU-Lb
5000 10000 15000 20000 25000 30000 35000 40000 45000 50000 55000
rf_book.Rickettsia_felis_URRWXCal2.pRF.ordered_contigs.stitched_ONE.fna

Supplementary fig. S6. Comparative analysis of diverse pRF plasmids.

(A) Plasmid pRF of *R. felis* str. URRWXCal2 was used as a reference to evaluate the conservation of its 68 CDS across the pRF plasmids of strains LSU, "AUS-Lb" and LSU-Lb. See text for details on orthologous group generation and analysis. Outer ring depicts the 68 CDS beginning at 12 o'clock and continuing clockwise. CDS denoted with asterisks depict truncated ORFs relative to larger homologs from other bacterial genomes. Ring 2: scale with coordinates (5 Kb intervals shown in larger numbers) as originally defined for pRF of *R. felis* str. URRWXCal2 [(Ogata, et al. 2005)]. Rings 3-6: CDS encoded on plasmid pRF of *R. felis* strains URRWXCal2, LSU, "AUS-Lb" and LSU-Lb. Inset (gray box) describes the CDS color scheme. CDS with significant similarity are connected by thick gray arcs across the interior of the plot. Thin blue arcs depict split ORFs that are conserved across all four plasmids. Thin red arcs depict two instances where adjacent CDS are fused as one CDS in strain LSU-Lb. Plot made using Circos [(Krzywinski, et al. 2009)] with manual adjustment.

(B) Annotations for the 68 proteins encoded on plasmid pRF. Proteins highlighted yellow illustrate the 15 CDS that are conserved in length across pRF plasmids from *R. felis* strains URRWXCal2, LSU, "AUS-Lb" and LSU-Lb. CDS denoted with asterisks depict truncated ORFs relative to larger homologs from other bacterial genomes. Annotations are further described elsewhere [(Gillespie, et al. 2007)].

Literature Cited

Gillespie JJ, et al. 2007. Plasmids and rickettsial evolution: insight from *Rickettsia felis*. PLos One 2: e266. doi: 10.1371/journal.pone.0000266

Krzywinski M, et al. 2009. Circos: an information aesthetic for comparative genomics. Genome research 19: 1639-1645. doi: gr.092759.109 [pii]
10.1101/gr.092759.109

Ogata H, et al. 2005. The genome sequence of *Rickettsia felis* identifies the first putative conjugative plasmid in an obligate intracellular parasite. PLoS Biol 3: e248. doi: 05-PLBI-RA-0181R2 [pii]
10.1371/journal.pbio.0030248

A

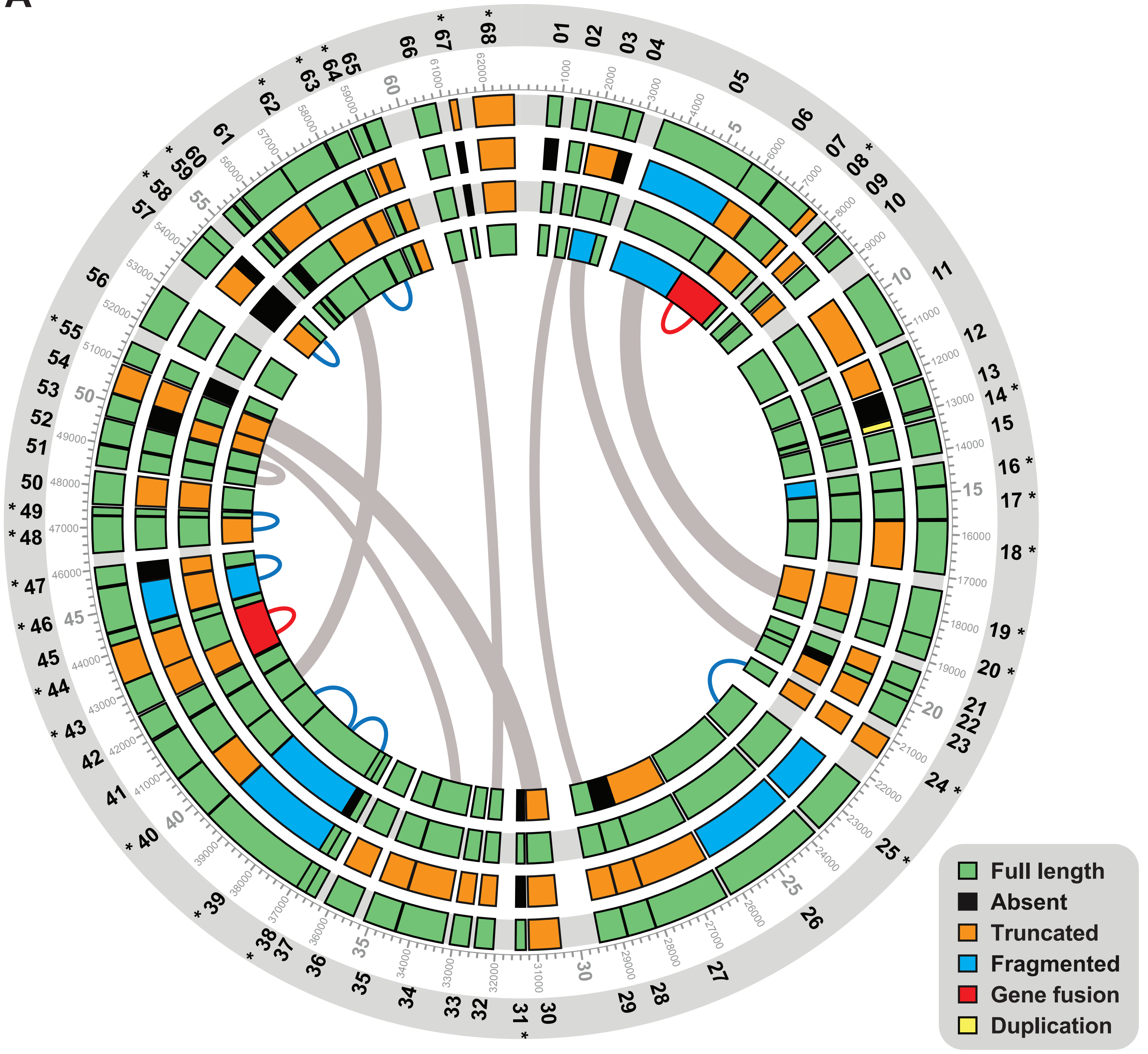

B

01: YP_247417: TNP
02: YP_247418: HTH_26
03: YP_247419: ParA
04: YP_247420: HP
05: YP_247421: DnaA_N
06: YP_247422: Hsd_R
07: YP_247423: Hsd_M
08: YP_247424: PleD *
09: YP_247425: HP
10: YP_247426: HP
11: YP_247427: Pat1B
12: YP_247428: HP
13: YP_247429: TMPK
14: YP_247430: ANK *
15: YP_247431: HP
16: YP_247432: TPR *
17: YP_247433: TPR *
18: YP_247434: TPR *
19: YP_247435: DnaA_N
20: YP_247436: DnaA_N
21: YP_247437: HTH_XRE
22: YP_247438: HP
23: YP_247439: ParA
24: YP_247440: Sca12 *
25: YP_247441: Sca12 *
26: YP_247442: Lon
27: YP_247443: SMC-like
28: YP_247444: HP
29: YP_247445: HTH_26
30: YP_247446: TNP
31: YP_247447: TNP *
32: YP_247448: PinR
33: YP_247449: HP
34: YP_247450: DnaQ
35: YP_247451: ParB
36: YP_247452: HP
37: YP_247453: TraD (Ti)
38: YP_247454: TraA (Ti) *
39: YP_247455: TraA (Ti) *
40: YP_247456: TraA (Ti) *
41: YP_247457: TNP
42: YP_247458: ANK
43: YP_247459: TraD (F) *
44: YP_247460: TraD (F) *
45: YP_247461: HP
46: YP_247462: TraG_C *
47: YP_247463: TraG_N *
48: YP_247464: ISRpe1 *
49: YP_247465: ISRpe1 *
50: YP_247466: HP
51: YP_247467: IbpA
52: YP_247468: IbpA
53: YP_247469: DnaQ *
54: YP_247470: TNP
55: YP_247471: TNP *
56: YP_247472: NAGidase
57: YP_247473: TNP
58: YP_247474: TNP *
59: YP_247475: HP
60: YP_247476: TNP
61: YP_247477: TNP
62: YP_247478: TraA2 (Ti) *
63: YP_247479: TraA2 (Ti) *
64: YP_247480: TNP *
65: YP_247481: HP
66: YP_247482: PinR
67: YP_247483: TNP *
68: YP_247484: HP

Supplementary fig. S7. *R. felis* str. LSU-Lb carries a novel rickettsial plasmid, pLbaR.

(A) Schema depicting the characteristics for plasmid of *Liposcelis bostrychophila* associated *Rickettsia* (pLbaR). The plasmid is shown circularized, though attempts to close the single contig at its ends (12 o'clock) with PCR failed (see text for more information). Plot made using Circos (Krzywinski, et al. 2009) with manual adjustment. The details for the seven rings are described below the plot.

(B) Annotations and other information for the 43 proteins encoded on plasmid pLbaR.

(C) Top BLASTP hits to each of the 43 proteins encoded on plasmid pLbaR. BLASTP searches were performed against the Rickettsiales database (*left*) and the nr database excluding Rickettsiales (*right*).

Literature Cited

Krzywinski M, et al. 2009. Circos: an information aesthetic for comparative genomics. Genome research 19: 1639-1645. doi: gr.092759.109 [pii]
10.1101/gr.092759.109

A

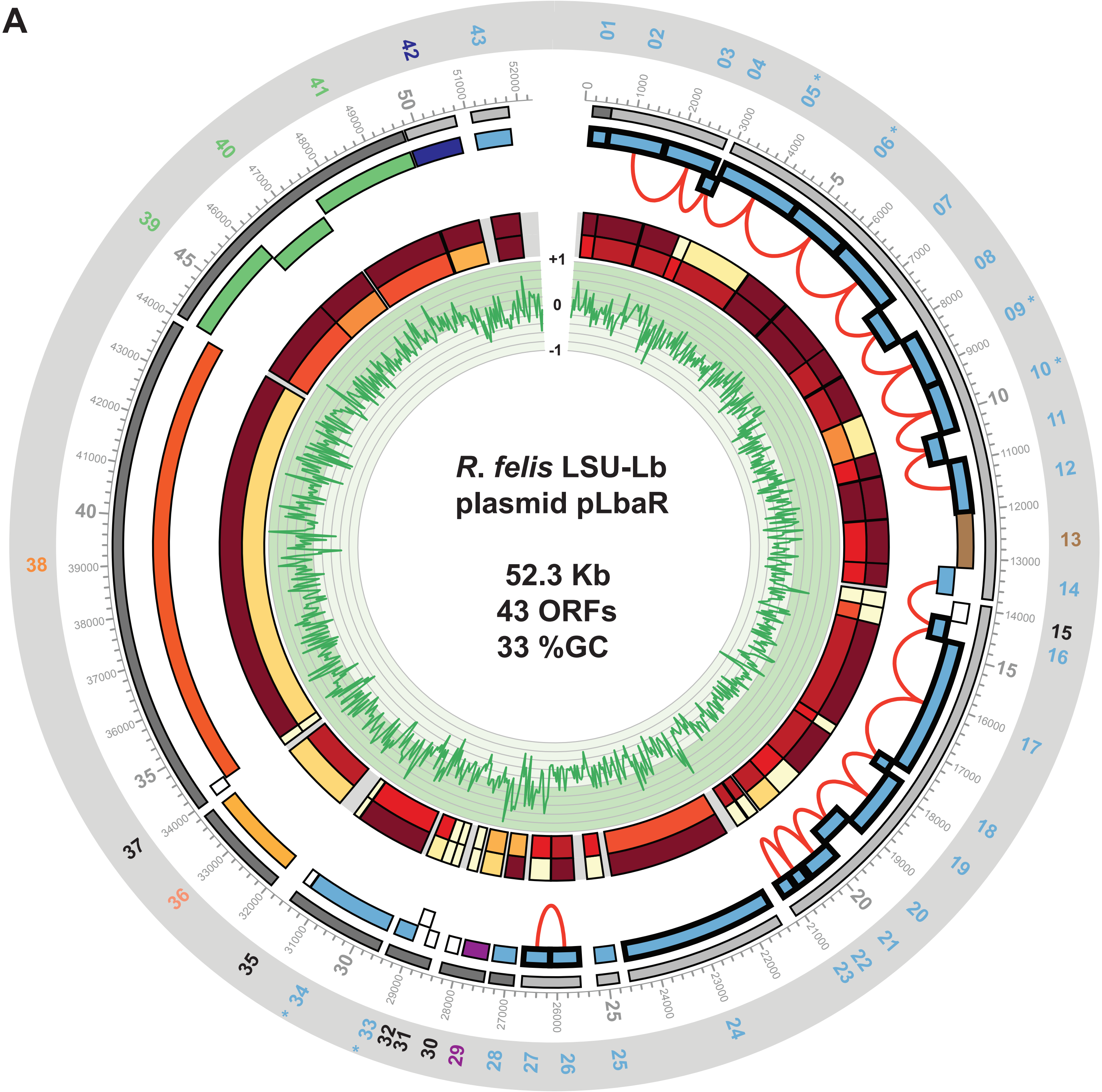

Ring	Description
1	CDS 01-43; annotations provided in panel B, BLASTP results provided in panel C. Asterisks, split ORFs.
2	Coordinates noted every 1000 nt, with 5 Kb intervals shown in larger numbers.
3	Predicted transcriptional units and operons: dark gray, forward strand; light gray, reverse strand.
4	Predicted CDS, colored by taxonomy of top BLASTP match: light blue, *Rickettsia* (heavy border = REIS specifically); brown, Cyanobacteria; white, no significant match; purple, *Deltaproteobacteria*; light orange, Rickettsiaceae bacterium Os18; dark orange, *Orientia*; green, *Gammaproteobacteria*; dark blue, Bacteroidetes. Red arcs depict genes syntenic with regions of the REIS genome.
5	Heatmap depicting number of BLASTP matches (yellow = 0, red = 50).
6	Heatmap depicting %ID of top BLASTP match (yellow = 0, red = 100).
7	GC skew, (G-C)/(G+C), calculated on a fixed 50 nt window. Spectra ranges from -1 (G = 0) to +1 (C = 0).

B

ID	Size (aa)	Description	Brief comment
01	119	transposase	Transposase (Mutator family, pfam00872), proliferated in REIS.
02	389	conjugative transfer protein TraD	RAGE F-T4SS, coupling protein TraD; truncated at CT.
03	362	lytic transglycosylase, catalytic	Fusion: NT (1-220) hits nothing, CT (221-362) MltE lytic transglycosylase.
04	105	hypothetical protein	Truncated 109 aa at NT relative to REIS homolog.
05	562	conserved hypothetical protein	RAGE F-T4SS, TraG; corresponds to 409-972 of TraG of pREIS3).
06	400	conserved hypothetical protein	RAGE F-T4SS, TraG; corresponds to 1-397 of TraG (pREIS3).
07	485	hypothetical protein	RAGE F-T4SS, TraH.
08	268	hypothetical protein	RAGE F-T4SS, TraF.
09	359	conserved hypothetical protein	RAGE F-T4SS, TraN; contains central and CT of TraN proteins.
10	340	hypothetical protein	RAGE F-T4SS, TraN; contains partial sequence of NT of TraN proteins.
11	196	type-F conjugative transfer system pilin assembly protein TrbC	RAGE F-T4SS, TrbC.
12	360	sex pilus assembly	RAGE F-T4SS, TraU.
13	376	hypothetical protein RF_1220	DUF1016: uncharacterized proteins, potentially nucleases.
14	196	type-F conjugative transfer system protein TraW	RAGE F-T4SS, TraW.
15	133	Tolq transport protein [SPURIOUS]	Hypothetical protein, no significant matches.
16	157	hypothetical protein	Hypothetical protein, only significant match is pREIS_2273.
17	926	inner-membrane protein TraC	RAGE F-T4SS, TraC.
18	91	hypothetical protein	RAGE F-T4SS, TraV.
19	428	TraB pilus assembly	RAGE F-T4SS, TraB.
20	240	hypothetical protein	RAGE F-T4SS, TraK; full length as on pREIS3 (split in all other RAGEs).
21	220	hypothetical protein	RAGE F-T4SS, TraE.
22	94	putative lipoprotein	Hypothetical protein known only from pREIS3.
23	104	hypothetical protein	Hypothetical protein known only from pREIS3.
24	1041	tetratricopeptide repeat domain protein	Fusion: NT LuxR-like domain, CT tetratricopeptide repeat domain.
25	140	hypothetical protein RPR_p19	Truncation of Sca4 variant encoded on pRPR, pREIS1 and pREIS3.
26	197	guanosine polyphosphate pyrophosphohydrolase/synthetase	SpoT synthetase.
27	179	cell surface antigen Sca12	Fragment corresponding to NT region of Sca12 of pREIS3.
28	158	hypothetical protein RMA_p12	HTH_26 [pfam13443]; Cro/C1-type HTH DNA-binding domain.
29	172	chromosome partitioning protein-like protein	ParA protein encoded on many *Rickettsia* plasmids.
30	84	hypothetical protein CRE_18161	Hypothetical protein, no significant matches.
31	69	small GTP-binding domain protein [SPURIOUS]	Hypothetical protein, no significant matches.
32	84	hypothetical protein	Hypothetical protein, only significant match is *R. australis* (WP_017208726).
33	133	DnaA-like protein	Split ORF: corresponds to 1-130 of DnaA (pRPR of *R. peacockii*).
34	592	DnaA-like protein	Split ORF: corresponds to 188-771 of DnaA (pRPR of *R. peacockii*).
35	54	hypothetical protein	Fragment of possible phage protein (DUF4065).
36	614	hypothetical protein	Present in Rickettsiaceae bacterium Os18 and *Wolbachia* species.
37	75	hypothetical protein Ftrac_1873	Hypothetical protein, no significant matches.
38	3241	hypothetical protein OTBS_0978	Multi-domains: OTU-like cysteine protease, Tox-SGS, ANK.
39	695	metal ABC transporter permease	T1SS C39-like ABC transporter; *Rickettsia*/*Cardinium* LGT.
40	454	hemolysin secretion protein D	T1SS membrane fusion protein; *Rickettsia*/*Cardinium* LGT.
41	710	cyclolysin secretion/processing ATP-binding protein CyaB	T1SS C39-like ABC transporter; *Rickettsia*/*Cardinium* LGT.
42	334	hypothetical protein rsib_orf696	Known mostly from Cytophaga-Flavobacterium-Bacteroides species.
43	242	chromosome segregation ATPase [SPURIOUS]	PD-(D/E)XK nuclease family transposase; *Rickettsia*/*Cardinium* LGT.

RAGE illustrated in **FIG 3**.

RTX-like T1SS illustrated in **FIG 4**.

Split ORFs.

C

ID	Rickettsiales Top hit	E-value	%ID		Non-Rickettsiales Top hit	E-value	%ID
01	*Rickettsia* endosymbiont (*Ixodes scapularis*)	4.00E-43	75.45		*Chlorobium phaeobacteroides* DSM 266	3.00E-34	58.65
02	*Rickettsia* endosymbiont (*Ixodes scapularis*)	0	86.65		*Candidatus* Protochlamydia amoebophila UWE25	1.00E-36	31.15
03	*Rickettsia* endosymbiont (*Ixodes scapularis*)	6.00E-63	83.33		*Mesorhizobium* sp. LSHC424B00	1.00E-20	43.9
04	*Rickettsia* endosymbiont (*Ixodes scapularis*)	5.00E-25	67.31		*Mycobacterium* sp. 360MFTsu5.1	0.47	29
05	*Rickettsia* endosymbiont (*Ixodes scapularis*)	0	87.23	*split*	*Fluoribacter dumoffii*	9.00E-05	34.58
06	*Rickettsia* endosymbiont (*Ixodes scapularis*)	0	91.94		uncultured bacterium	8.00E-34	31.98
07	*Rickettsia* endosymbiont (*Ixodes scapularis*)	0	89.13		uncultured bacterium	4.00E-26	26.74
08	*Rickettsia* endosymbiont (*Ixodes scapularis*)	7.00E-124	83.08		*Mariprofundus ferrooxydans*	6.00E-15	25.44
09	*Rickettsia* endosymbiont (*Ixodes scapularis*)	3.00E-168	81.23	*split*	*Escherichia coli*	3.00E-35	33.01
10	*Rickettsia* endosymbiont (*Ixodes scapularis*)	7.00E-96	53.91		*Clostridium symbiosum* WAL-14673	2.9	30
11	*Rickettsia* endosymbiont (*Ixodes scapularis*)	9.00E-76	73.47		uncultured bacterium	6.00E-09	35.71
12	*Rickettsia* endosymbiont (*Ixodes scapularis*)	0	89.17		*Sphingomonas* sp. KA1	2.00E-87	46.69
13	*Rickettsia felis* URRWXCal2	5.00E-56	38.54		uncultured bacterium	6.00E-136	68.53
14	*Candidatus* Rickettsia amblyommii AaR/SC	7.00E-79	76.44		*Gallionella capsiferriformans* ES-2	2.00E-23	34.25
15	*Candidatus* Midichloria mitochondrii IricVA	8.60E+00	55		*Clostridium* sp. CAG:58	4.30E-01	28
16	*Rickettsia* endosymbiont (*Ixodes scapularis*)	1.00E-41	63.45		*Nannochloropsis gaditana*	1.5	26
17	*Rickettsia* endosymbiont (*Ixodes scapularis*)	0	88.66		uncultured bacterium	2.00E-72	25.96
18	*Rickettsia* endosymbiont (*Ixodes scapularis*)	1.00E-28	77.63		Lachnospiraceae bacterium 3-1	8.00E-06	31
19	*Rickettsia* endosymbiont (*Ixodes scapularis*)	0	84.11		*Thiobacillus denitrificans*	2.00E-27	37.02
20	*Rickettsia* endosymbiont (*Ixodes scapularis*)	1.00E-103	77.5		*Thiobacillus denitrificans*	0.002	24.21
21	*Rickettsia* endosymbiont (*Ixodes scapularis*)	3.00E-103	86.82		*Aromatoleum aromaticum* EbN1	3.00E-05	22.22
22	*Rickettsia* endosymbiont (*Ixodes scapularis*)	9.00E-38	82.98		*Dictyostelium discoideum* AX4	3.5	38
23	*Rickettsia* endosymbiont (*Ixodes scapularis*)	3.00E-38	85.86		*Streptomyces acidiscabies*	0.26	44
24	*Rickettsia* endosymbiont (*Ixodes scapularis*)	0	56.03		uncultured bacterium	6.00E-139	30.6
25	*Rickettsia peacockii* Rustic	7.00E-37	76.7		*Schizophyllum commune* H4-8	0.42	36
26	*Rickettsia* endosymbiont (*Ixodes scapularis*)	2.00E-85	78.68		*Genlisea aurea*	4.00E-32	58
27	*Rickettsia* endosymbiont (*Ixodes scapularis*)	5.00E-20	68.69		*Sclerotinia sclerotiorum* 1980	4	34
28	*Rickettsia massiliae* MTU5	4.00E-26	38.99		*Peptoclostridium difficile*	4.00E-04	38
29	*Candidatus* Rickettsia amblyommii GAT-30V	0.056	42.62		SAR324 cluster bacterium JCVI-SC AAA005	2.00E-22	36.94
30	------	------	------		*Caenorhabditis remanei*	2.20E+00	35
31	------	------	------		*Tetrahymena thermophila* SB210	6.70E+00	48
32	*Rickettsia australis*	8.00E-04	33		*Deinococcus peraridilitoris* DSM 19664	5.10E+00	35
33	*Rickettsia peacockii* Rustic	1.00E-42	66.92	*split*	*Psychrobacter* sp. JCM 18903	0.12	38
34	*Rickettsia peacockii* Rustic	0	68.13		*Ixodes scapularis*	4.00E-94	73
35	------	------	------		------	------	------
36	Rickettsiaceae bacterium Os18	0	79.13		*Alligator sinensis*	0.23	25
37	------	------	------		*Marivirga tractuosa* DSM 4126	0.67	32
38	*Orientia tsutsugamushi* Boryong	3.00E-162	31.68		*Diplorickettsia massiliensis*	5.00E-38	27.06
39	*Candidatus* Odyssella thessalonicensis	2.00E-75	32		*Moritella* sp. PE36	0	57.58
40	*Rickettsia canadensis*	3.00E-20	29.14		*Photorhabdus luminescens* subsp. laumondii TTO1	8.00E-102	45.67
41	*Rickettsia* endosymbiont (*Ixodes scapularis*)	4.00E-96	92.5		*Vibrio nigripulchritudo*	0	58
42	*Rickettsia sibirica* 246	2.60E-01	25.96		*Proteiniphilum acetatigenes*	4.00E-45	35.83
43	*Rickettsia monacensis*	3.00E-130	95.02		*Cardinium* endosymbiont cEper1 (*Encarsia pergandiella*)	3.00E-107	78.84

(yellow) Illustrtaes the stronger hit across the two databases.

(pink) Split ORFs.

Supplementary fig. S8. Phylogeny estimation of DUF1016 proteins.

All proteins are putative members of the PD-(D/E)XK phosphodiesterase superfamily. The YhcG protein of *Escherichia coli*, as well as putative uncharacterized phage proteins of some Rickettsiales, are noted. All other proteins are annotated as 'hypothetical protein'. The sequence encoded on the pLbaR plasmid of *Rickettsia felis* str. LSU-Lb is highlighted yellow (see **fig. 3** for more information). Sequences were obtained from NCBI prior to April 20th, 2014 (accession numbers provided after species names) and aligned using MUSCLE v3.6 (default parameters) (Edgar 2004). Phylogeny was estimated under maximum likelihood using RAxML v.7.2.8 (Stamatakis, et al. 2008). Tree is final optimization likelihood: (-32464.484679) using WAG substitution model with GAMMA and proportion of invariant sites estimated. Brach support is from 1000 bootstrap pseudoreplications.

Literature Cited

Edgar RC 2004. MUSCLE: multiple sequence alignment with high accuracy and high throughput. Nucleic acids research 32: 1792-1797. doi: 10.1093/nar/gkh340
32/5/1792 [pii]

Stamatakis A, Hoover P, Rougemont J 2008. A rapid bootstrap algorithm for the RAxML Web servers. Syst Biol 57: 758-771. doi: 904007647 [pii]
10.1080/10635150802429642

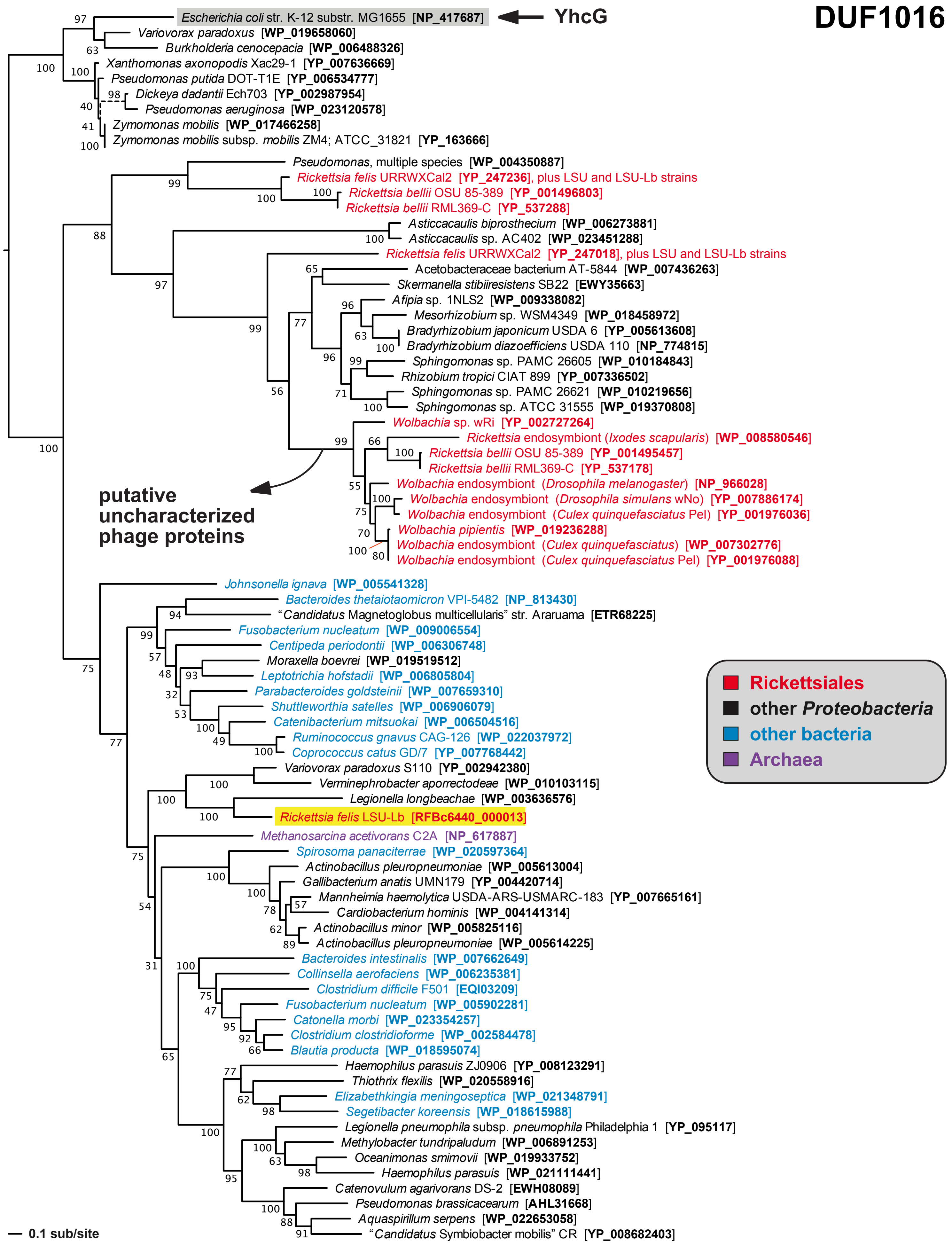
DUF1016
YhcG
Escherichia coli str. K-12 substr. MG1655 [NP_417687]
Variovorax paradoxus [WP_019658060]
Burkholderia cenocepacia [WP_006488326]
Xanthomonas axonopodis Xac29-1 [YP_007636669]
Pseudomonas putida DOT-T1E [YP_006534777]
Dickeya dadantii Ech703 [YP_002987954]
Pseudomonas aeruginosa [WP_023120578]
Zymomonas mobilis [WP_017466258]
Zymomonas mobilis subsp. mobilis ZM4; ATCC_31821 [YP_163666]
Pseudomonas, multiple species [WP_004350887]
Rickettsia felis URRWXCal2 [YP_247236], plus LSU and LSU-Lb strains
Rickettsia bellii OSU 85-389 [YP_001496803]
Rickettsia bellii RML369-C [YP_537288]
Asticcacaulis biprosthecium [WP_006273881]
Asticcacaulis sp. AC402 [WP_023451288]
Rickettsia felis URRWXCal2 [YP_247018], plus LSU and LSU-Lb strains
Acetobacteraceae bacterium AT-5844 [WP_007436263]
Skermanella stibiiresistens SB22 [EWY35663]
Afipia sp. 1NLS2 [WP_009338082]
Mesorhizobium sp. WSM4349 [WP_018458972]
Bradyrhizobium japonicum USDA 6 [YP_005613608]
Bradyrhizobium diazoefficiens USDA 110 [NP_774815]
Sphingomonas sp. PAMC 26605 [WP_010184843]
Rhizobium tropici CIAT 899 [YP_007336502]
Sphingomonas sp. PAMC 26621 [WP_010219656]
Sphingomonas sp. ATCC 31555 [WP_019370808]
Wolbachia sp. wRi [YP_002727264]
Rickettsia endosymbiont (Ixodes scapularis) [WP_008580546]
Rickettsia bellii OSU 85-389 [YP_001495457]
Rickettsia bellii RML369-C [YP_537178]
Wolbachia endosymbiont (Drosophila melanogaster) [NP_966028]
Wolbachia endosymbiont (Drosophila simulans wNo) [YP_007886174]
Wolbachia endosymbiont (Culex quinquefasciatus Pel) [YP_001976036]
Wolbachia pipientis [WP_019236288]
Wolbachia endosymbiont (Culex quinquefasciatus) [WP_007302776]
Wolbachia endosymbiont (Culex quinquefasciatus Pel) [YP_001976088]
putative uncharacterized phage proteins
Johnsonella ignava [WP_005541328]
Bacteroides thetaiotaomicron VPI-5482 [NP_813430]
"Candidatus Magnetoglobus multicellularis" str. Araruama [ETR68225]
Fusobacterium nucleatum [WP_009006554]
Centipeda periodontii [WP_006306748]
Moraxella boevrei [WP_019519512]
Leptotrichia hofstadii [WP_006805804]
Parabacteroides goldsteinii [WP_007659310]
Shuttleworthia satelles [WP_006906079]
Catenibacterium mitsuokai [WP_006504516]
Ruminococcus gnavus CAG-126 [WP_022037972]
Coprococcus catus GD/7 [YP_007768442]
Variovorax paradoxus S110 [YP_002942380]
Verminephrobacter aporrectodeae [WP_010103115]
Legionella longbeachae [WP_003636576]
Rickettsia felis LSU-Lb [RFBc6440_000013]
Methanosarcina acetivorans C2A [NP_617887]
Spirosoma panaciterrae [WP_020597364]
Actinobacillus pleuropneumoniae [WP_005613004]
Gallibacterium anatis UMN179 [YP_004420714]
Mannheimia haemolytica USDA-ARS-USMARC-183 [YP_007665161]
Cardiobacterium hominis [WP_004141314]
Actinobacillus minor [WP_005825116]
Actinobacillus pleuropneumoniae [WP_005614225]
Bacteroides intestinalis [WP_007662649]
Collinsella aerofaciens [WP_006235381]
Clostridium difficile F501 [EQI03209]
Fusobacterium nucleatum [WP_005902281]
Catonella morbi [WP_023354257]
Clostridium clostridioforme [WP_002584478]
Blautia producta [WP_018595074]
Haemophilus parasuis ZJ0906 [YP_008123291]
Thiothrix flexilis [WP_020558916]
Elizabethkingia meningoseptica [WP_021348791]
Segetibacter koreensis [WP_018615988]
Legionella pneumophila subsp. pneumophila Philadelphia 1 [YP_095117]
Methylobacter tundripaludum [WP_006891253]
Oceanimonas smirnovii [WP_019933752]
Haemophilus parasuis [WP_021111441]
Catenovulum agarivorans DS-2 [EWH08089]
Pseudomonas brassicacearum [AHL31668]
Aquaspirillum serpens [WP_022653058]
"Candidatus Symbiobacter mobilis" CR [YP_008682403]
Rickettsiales
other Proteobacteria
other bacteria
Archaea
0.1 sub/site

Supplementary fig. S9. Plasmid pLbaR of *R. felis* str. LSU-Lb encodes an RHS-like toxin adjacent to an RTX-like T1SS operon.

(A) Schema of region 34173-49728 of plasmid pLbaR, which depicts the genes encoding the recombination hot spot (RHS) toxin (pLbaR_38) and the RTX-like T1SS (pLbaR39-41). The gene models are drawn to scale. See **fig. 4** for further description of the RTX-like T1SS.

(B) Characteristics of the RHS-like toxin encoded on pLbaR. Three distinct domains, colored brown, were identified: 1) an NT (pos. 22-180) OTU-like cysteine protease domain, pfam02338 [(Makarova, et al. 2000)], 2) an internal (pos. 2813-2924) salivary gland secreted toxin (Tox-SGS) domain, pfam15651 [(Arca, et al. 2005; Korochkina, et al. 2006)], and 3) a CT ankyrin (ANK) repeat domain (pos. 3079-3161), which contains two ANK repeats. The top BLASTP hits to pLbaR_38 are shown below the schema, with regions of similarity color coded according to taxa. NOTE: BLASTP hits to the ANK domain are not shown.

(C) pLbaR_38 encodes an OTU-like cysteine protease domain. pfam02338 is comprised of cysteine proteases that are similar to the Ovarian tumor (OTU) protein of *Drosophila* spp. [(Makarova, et al. 2000)]. Residues within the active site (Asp, Cys and His) are highlighted yellow across the alignment of pLbaR_38 and eight diverse OTU and OTU-like proteins. These proteins have been shown to play a role in deubiquitinization [(Balakirev, et al. 2003)].

(D) pLbaR_38 encodes a Salivary Gland Secreted protein domain toxin. pfam15651 (Tox-SGS) contains proteins with an alpha+beta fold domain, which contains four conserved Cys residues and a conserved [DE}xx[ND] motif [(Arca, et al. 2005; Korochkina, et al. 2006)]. These residues are highlighted yellow across the alignment of pLbaR_38 and eight diverse proteins from various mosquito species. The Tox-SGS domain is mainly present at the C-terminus of RHS proteins from arthropods and crustaceans. While few bacterial homologs have been identified, the domain architecture indicates an origin from bacterial polymorphic toxin systems [(Zhang, et al. 2012)].

Literature Cited

Arca B, et al. 2005. An updated catalogue of salivary gland transcripts in the adult female mosquito, *Anopheles gambiae*. The Journal of experimental biology 208: 3971-3986. doi: 10.1242/jeb.01849

Balakirev MY, Tcherniuk SO, Jaquinod M, Chroboczek J 2003. Otubains: a new family of cysteine proteases in the ubiquitin pathway. EMBO reports 4: 517-522. doi: 10.1038/sj.embor.embor824

Korochkina S, et al. 2006. A mosquito-specific protein family includes candidate receptors for malaria sporozoite invasion of salivary glands. Cellular microbiology 8: 163-175. doi: 10.1111/j.1462-5822.2005.00611.x

Makarova KS, Aravind L, Koonin EV 2000. A novel superfamily of predicted cysteine proteases from eukaryotes, viruses and *Chlamydia pneumoniae*. Trends in biochemical sciences 25: 50-52.

Zhang D, de Souza RF, Anantharaman V, Iyer LM, Aravind L 2012. Polymorphic toxin systems: Comprehensive characterization of trafficking modes, processing, mechanisms of action, immunity and ecology using comparative genomics. Biology direct 7: 18. doi: 10.1186/1745-6150-7-18

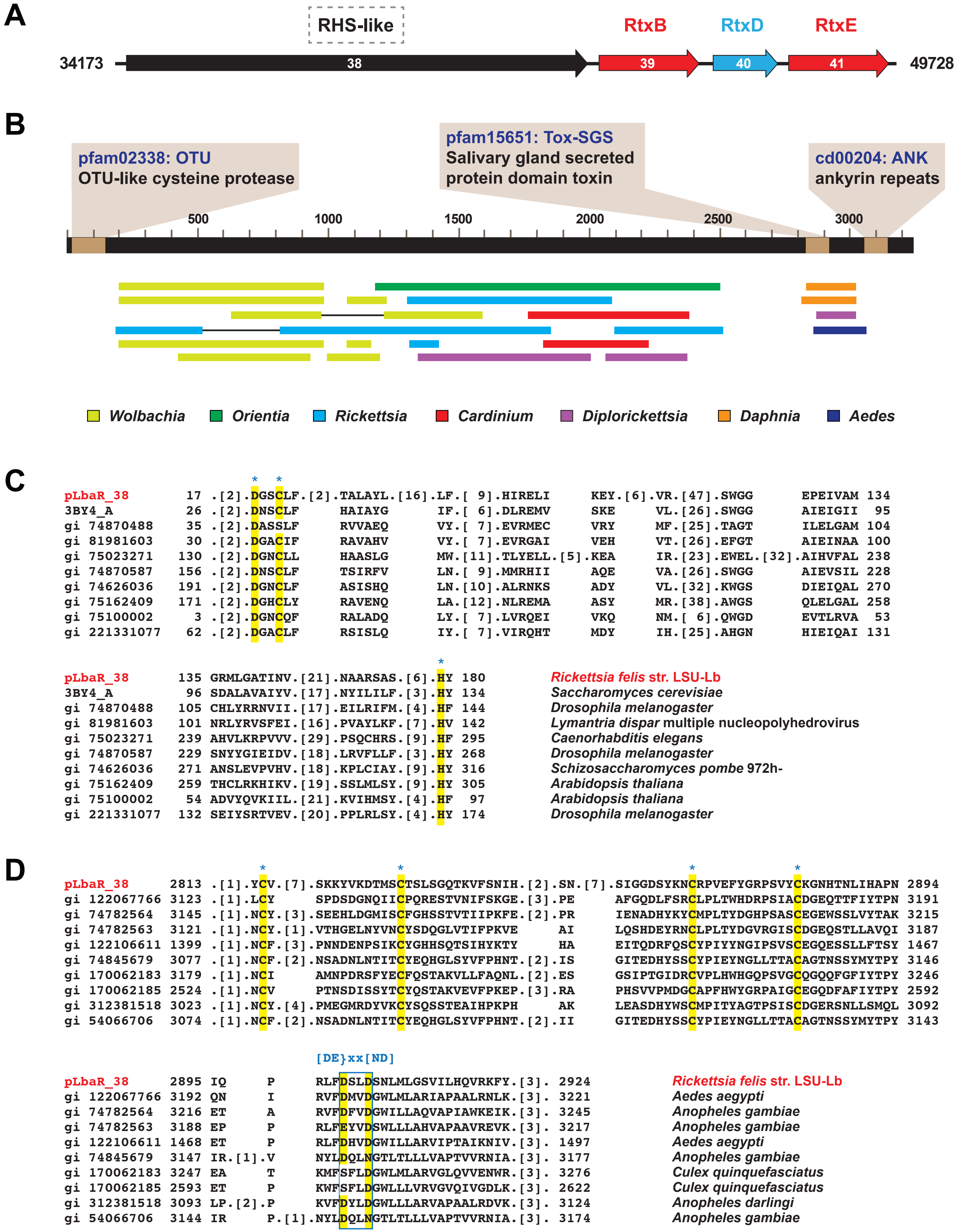
A
RHS-like
RtxB
RtxD
RtxE
34173
38
39
40
41
49728
B
pfam02338: OTU
OTU-like cysteine protease
pfam15651: Tox-SGS
Salivary gland secreted
protein domain toxin
cd00204: ANK
ankyrin repeats
500
1000
1500
2000
2500
3000
Wolbachia
Orientia
Rickettsia
Cardinium
Diplorickettsia
Daphnia
Aedes
C
pLbaR_38 17 .[2].DGSCLF.[2].TALAYL.[16].LF.[9].HIRELI KEY.[6].VR.[47].SWGG EPEIVAM 134
3BY4_A 26 .[2].DNSCLF HAIAYG IF.[6].DLREMV SKE VL.[26].SWGG AIEIGII 95
gi 74870488 35 .[2].DASSLF RVVAEQ VY.[7].EVRMEC VRY MF.[25].TAGT ILELGAM 104
gi 81981603 30 .[2].DGACIF RAVAHV VY.[7].EVRGAI VEH VT.[26].EFGT AIEINAA 100
gi 75023271 130 .[2].DGNCLL HAASLG MW.[11].TLYELL.[5].KEA IR.[23].EWEL.[32].AIHVFAL 238
gi 74870587 156 .[2].DNSCLF TSIRFV LN.[9].MMRHII AQE VA.[26].SWGG AIEVSIL 228
gi 74626036 191 .[2].DGNCLF ASISHQ LN.[10].ALRNKS ADY VL.[32].KWGS DIEIQAL 270
gi 75162409 171 .[2].DGHCLY RAVENQ LA.[12].NLREMA ASY MR.[38].AWGS QLELGAL 258
gi 75100002 3 .[2].DGNCQF RALADQ LY.[7].LVRQEI VKQ NM.[6].QWGD EVTLRVA 53
gi 221331077 62 .[2].DGACLF RSISLQ IY.[7].VIRQHT MDY IH.[25].AHGN HIEIQAI 131
pLbaR_38 135 GRMLGATINV.[21].NAARSAS.[6].HY 180 Rickettsia felis str. LSU-Lb
3BY4_A 96 SDALAVAIYV.[17].NYILILF.[3].HY 134 Saccharomyces cerevisiae
gi 74870488 105 CHLYRRNVII.[17].EILRIFM.[4].HF 144 Drosophila melanogaster
gi 81981603 101 NRLYRVSFEI.[16].PVAYLKF.[7].HV 142 Lymantria dispar multiple nucleopolyhedrovirus
gi 75023271 239 AHVLKRPVVV.[29].PSQCHRS.[9].HF 295 Caenorhabditis elegans
gi 74870587 229 SNYYGIEIDV.[18].LRVFLLF.[3].HY 268 Drosophila melanogaster
gi 74626036 271 ANSLEVPVHV.[18].KPLCIAY.[9].HY 316 Schizosaccharomyces pombe 972h-
gi 75162409 259 THCLRKHIKV.[19].SSLMLSY.[9].HY 305 Arabidopsis thaliana
gi 75100002 54 ADVYQVKIIL.[21].KVIHMSY.[4].HF 97 Arabidopsis thaliana
gi 221331077 132 SEIYSRTVEV.[20].PPLRLSY.[4].HY 174 Drosophila melanogaster
D
pLbaR_38 2813 .[1].YCV.[7].SKKYVKDTMSCTSLSGQTKVFSNIH.[2].SN.[7].SIGGDSYKNCRPVEFYGRPSVYCKGNHTNLIHAPN 2894
gi 122067766 3123 .[1].LCY SPDSDGNQIICPQRESTVNIFSKGE.[3].PE AFGQDLFSRCLPLTWHDRPSIACDGEQTTFIYTPN 3191
gi 74782564 3145 .[1].NCY.[3].SEEHLDGMISCFGHSSTVTIIPKFE.[2].PR IENADHYKYCMPLTYDGHPSASCEGEWSSLVYTAK 3215
gi 74782563 3121 .[1].NCY.[1].VTHGELNYVNCYSDQGLVTIFPKVE AI LQSHDEYRNCLPLTYDGVRGISCDGEQSTLLAVQI 3187
gi 122106611 1399 .[1].NCF.[3].PNNDENPSIKCYGHHSQTSIHYKTY HA EITQDRFQSCYPIYYNGIPSVSCEGEQESSLLFTSY 1467
gi 74845679 3077 .[1].NCF.[2].NSADNLNTITCYEQHGLSYVFPHNT.[2].IS GITEDHYSSCYPIEYNGLLTTACAGTNSSYMYTPY 3146
gi 170062183 3179 .[1].NCI AMNPDRSFYECFQSTAKVLLFAQNL.[2].ES GSIPTGIDRCVPLHWHGQPSVGCQGQQFGFIYTPY 3246
gi 170062185 2524 .[1].NCV PTNSDISSYTCYQSTAKVEVFPKEP.[3].RA PHSVVPMDGCAPFHWYGRPAIGCEGQDFAFIYTPY 2592
gi 312381518 3023 .[1].NCY.[4].PMEGMRDYVKCYSQSSTEAIHPKPH AK LEASDHYWSCMPITYAGTPSISCDGERSNLLSMQL 3092
gi 54066706 3074 .[1].NCF.[2].NSADNLNTITCYEQHGLSYVFPHNT.[2].II GITEDHYSSCYPIEYNGLLTTACAGTNSSYMYTPY 3143
[DE}xx[ND]
pLbaR_38 2895 IQ P RLFDSLDSNLMLGSVILHQVRKFY.[3]. 2924 Rickettsia felis str. LSU-Lb
gi 122067766 3192 QN I RVFDMVDGWLMLARIAPAALRNLK.[3]. 3221 Aedes aegypti
gi 74782564 3216 ET A RVFDFVDGWILLAQVAPIAWKEIK.[3]. 3245 Anopheles gambiae
gi 74782563 3188 EP P RLFEYVDSWLLLAHVAPAAVREVK.[3]. 3217 Anopheles gambiae
gi 122106611 1468 ET P RLFDHVDGWILLARVIPTAIKNIV.[3]. 1497 Aedes aegypti
gi 74845679 3147 IR.[1].V NYLDQLNGTLTLLLVAPTVVRNIA.[3]. 3177 Anopheles gambiae
gi 170062183 3247 EA T KMFSFLDGWLMLARVGLQVVENWR.[3]. 3276 Culex quinquefasciatus
gi 170062185 2593 ET P KWFSFLDGWLLLVRVGVQIVGDLK.[3]. 2622 Culex quinquefasciatus
gi 312381518 3093 LP.[2].P KVFDYLDGWLLLARVAPAALRDVK.[3]. 3124 Anopheles darlingi
gi 54066706 3144 IR P.[1].NYLDQLNGTLTLLLVAPTVVRNIA.[3]. 3174 Anopheles gambiae
