# Supplementary material for: Genomic Diversification in Strains of Rickettsia felis Isolated from Different Arthropods
Source: Genome Biol Evol. 2014 Dec 4;7(1):35–56. doi: 10.1093/gbe/evu262 (PMC4316617; doi:10.1093/gbe/evu262)
Supplement: Supplementary Data [file supp_evu262_Supp_Mat_Tables_1-3.pdf]

Supplementary table S1. Primers used to confirm predicted SNPs.

***R. felis* str. LSU**

Reference [1]	SNP [2]	Primer name	Sequence [3]	Method [4]
YP_246280	1	Flea-GV00001_F<br>Flea-GV00001_R	GATTCCGGTTGCAATGACGG<br>TGATCGATAATACGCCCCGG	Direct
YP_246397	1	Flea-GV00002_F<br>Flea-GV00002_R	GTACCGAGAGTTGCAAGGCA<br>GCCGTTTCCTGTAGTTTGGG	Cloning
YP_246455	1	Flea-GV00003_F<br>Flea-GV00003_R	GGGCTGCTATTGTTCCGAGT<br>CGTTAAGCTGCACTTTGTTTCCT	Cloning
YP_246493	1	Flea-GV00004_F<br>Flea-GV00004_R	AGCGTTGTTTCTTCGGGGAT<br>TGCAGTTTGATCGCCCGTAT	Direct
YP_246703	1	Flea-GV00005_F<br>Flea-GV00005_R	TGCTTACGCAACAACACAGG<br>CCACCCTCTGCAACGGTAAA	Direct
YP_246853	2	Flea-GV00006-7_F<br>Flea-GV00006-7_R	ATGCAGGACCTAAGCCATGG<br>AATCAATGACCTCCTGCCCG	Direct
YP_246866-<br>YP_246867	10	Flea-GV00008-17_F<br>Flea-GV00008-17_R	TCTCGACTAAACTCCAAACAGCT<br>AGAGACGCAGCAGTTAGAACT	Direct
YP_246867	1	Flea-GV000018_F<br>Flea-GV000018_R	AGTTCTAACTGCTGCGTCTCT<br>TCCCTAGCTTAGCAAAAGAAGC	Direct
YP_246869	3	Flea-GV000019-21_F<br>Flea-GV000019-21_R	TCCGATAAATAGCCGCTGTCT<br>AAAGCAGAAGAAACGGGCGA	Direct
YP_246869	1	Flea-GV000022_F<br>Flea-GV000022_R	TGCTTTGAGCTAACAAGTGCA<br>AGGCTGGGATAGTGAGGGAA	Direct
YP_247161	1	Flea-GV000023_F<br>Flea-GV000023_R	ATAGGACGTCCGTTTGCAGG<br>GGTCGTTTCCGTCAGAACCT	Direct

***R. felis* str. LSU-Lb**

Reference [1]	SNP [2]	Primer name	Sequence [3]	Method [4]
YP_246042	1	Lice-GV00002_F<br>Lice-GV00002_R	AGGCAATCATCCTAAGGTTGCT<br>CCGGAGCGTTTTTCTCAAGG	Direct
YP_246394	1	Lice-GV00015_F<br>Lice-GV00015_R	ACTGGAGTTAGAGACGGCGA<br>AGACCGCGGGATCCATTTTT	Direct
YP_246425	1	Lice-GV00019_F<br>Lice-GV00019_R	AAAGCCCCAGGTTTTGGTGA<br>CACCGTTTTCACCGGCATTT	Direct
rRNA	1	Lice-GV00022_F<br>Lice-GV00022_R	CCGACGATCTGTAGCTGGTC<br>AGCTGCGAAACCGAAAGAGA	Direct
YP_246641	1	Lice-GV00040_F<br>Lice-GV00040_R	GGTGGCTGTTTACAACAATGGG<br>ACCATACACAACCACCTCCT	Direct

R. felis str. LSU-Lb (Cont'd.)

Reference [1]	SNP [2]	Primer name	Sequence [3]	Method [4]
YP_246654	1	Lice-GV00041_F<br>Lice-GV00041_R	AAGCAAATCGCCTGTACCGT<br>TCTTGGAGTCGGAACAACGG	Cloning
YP_246658	1	Lice-GV00044_F<br>Lice-GV00044_R	AGTGCCGCTATTTGGGTCAA<br>AGGAGCGGCTGAAACTTACC	Cloning
YP_246683	1	Lice-GV00047_F<br>Lice-GV00047_R	CTGCTTGCGGAGTTTGCTTT<br>TATCTGGCTAGCCCCCTCAG	Direct
YP_246887	1	Lice-GV00057_F<br>Lice-GV00057_R	GCACCTCTTGACCGCATTTC<br>TGCAAATGCCAGCCAAACAA	Direct
YP_246894	1	Lice-GV00058_F<br>Lice-GV00058_R	AGCGATGTAGCAGTACAGTCA<br>TGCAACGACTTGTGGAGGTT	Direct
YP_247229	1	Lice-GV00074_F<br>Lice-GV00074_R	GACCATCTAAGTCCTCCGCG<br>GGCACTTGGGATGCTTGCTA	Cloning
YP_247265	1	Lice-GV00078_F<br>Lice-GV00078_R	ATTTCAGCATGCACGCAAGG<br>GTTGCGAAAGACTCTTCGGC	Direct
YP_247304	1	Lice-GV00079_F<br>Lice-GV00079_R	GCCATTGCTTACTACTTGGTCC<br>GATGCCCTCCATGAGAAGCA	Direct
RR	1	Lice-GV00086_F<br>Lice-GV00086_R	GCATCTGTCCTTCCTGACCC<br>TTAGAAGCGGTGGAAAGCGT	Cloning
YP_247455	1	Lice-GV00091_F<br>Lice-GV00091_R	AGTAGAGTACAGGCAGGCGA<br>ACATGAGCACCAAAAACGCC	Cloning

[1] Protein accession number (NCBI) for *R. felis* str. URRWXCal2. CDS flanking SNPs are underlined. Protein names and other information is provided in **supplementary table S4**. RR, repeat region (position 1446564).

[2] Number of SNPs within each region targeted by primers.

[3] 5' to 3' orientation.

[4] Direct sequencing of PCR amplicons, or sequencing after cloning into vectors (see text for further details).

Supplementary table S2. Rickettsiaceae genomes utilized for phylogenomics analysis.

Taxon [1]	ID [2]	No [3].	%GC	L (bases)	CDS [4]	Pl. [5]
**Scrub Typhus Group**						
* Rickettsiaceae bacterium Os18 [NZ_CANJ00000000]	148284	301	29.05	1469247	1310	0
Orientia tsutsugamushi Ikeda [NC_009488]	129072	1	30.51	2008987	2197	0
Orientia tsutsugamushi Boryong [NC_010793]	83812	1	30.53	2127051	2364	0
**Ancestral Group**						
* *Rickettsia* sp. MEAM1 (*Bemisia tabaci*) [NA]	241202	607	32.13	1105415	1036	0
Rickettsia bellii RML369-C [NC_007940]	102610	1	31.65	1522076	1612	0
Rickettsia bellii OSU 85-389 [NC_009883]	35792	1	31.63	1528980	1657	0
Rickettsia canadensis CA410 [NC_009879]	238964	1	31.01	1150228	1130	0
Rickettsia canadensis McKiel [NC_016929]	89738	1	31.05	1159772	1230	0
Incertae sedis						
Rickettsia helvetica C9P9 [NZ_CM001467, NZ_CM001468]	217856	1	32.20	1369827	1739	1
**Transitional Group**						
Rickettsia felis URRWXCal2 [NC_007109, NC_007110]	64634	1	32.40	1485148	1810	1
* *Rickettsia felis* LSU [NA]	(new here)	21	32.35	1483097	2194	1
* *Rickettsia felis* LSU-Lb [NA]	(new here)	35	32.45	1467654	1831	2
Rickettsia akari Hartford [NC_009881]	50705	1	32.33	1231060	1437	0
Rickettsia australis Cutlack [NC_017058]	231019	1	32.25	1296670	1565	1
* *Rickettsia australis* Phillips [NA]	AKVZ01	45	32.21	1274508	1525	2**?**

Taxon [1]	ID [2]	No [3].	%GC	L (bases)	CDS [4]	Pl. [5]
**Typhus Group**						
Rickettsia typhi TH1527 [NC_006142]	188083	1	28.92	1112372	875	0
Rickettsia typhi B9991CWPP [NC_017062]	187203	1	28.92	1112957	877	0
Rickettsia typhi Wilmington [NC_017066]	34752	1	28.92	1111496	892	0
Rickettsia prowazekii GvV257 [NC_017048]	231244	1	28.99	1111969	902	0
Rickettsia prowazekii RpGvF24 [NC_017057]	237744	1	28.99	1112101	897	0
* *Rickettsia prowazekii* GvF12 [NA]	264402	10	29.00	1109257	966	0
Rickettsia prowazekii BuV67-CWPP [NC_017056]	229008	1	29.00	1111445	901	0
Rickettsia prowazekii Katsinyian [NC_017050]	230392	1	29.00	1111454	902	0
* *Rickettsia prowazekii* NMRC Madrid E [NC_020992]	275611	1	29.00	1111520	926	0
Rickettsia prowazekii Madrid E [NC_000963]	72556	1	29.00	1111523	924	0
Rickettsia prowazekii Rp22 [NC_017560]	89493	1	29.00	1111612	910	0
* *Rickettsia prowazekii* Cairo 3 [NA]	275463	20	29.07	1113960	900	0
Rickettsia prowazekii Chernikova [NC_017049]	240232	1	29.01	1109804	892	0
Rickettsia prowazekii Dachau [NC_017051]	238803	1	29.01	1108946	907	0
* *Rickettsia prowazekii* Breinl [NC_020993]	269054	1	29.01	1109301	914	0
**Spotted Fever Group**						
Rickettsia endosymbiont of *Ixodes scapularis* [CM000770, CM000771-3]	40569	16	32.39	1776032	2404	4
* *Rickettsia monacensis* IrR/Munich [NA]	274770	88	32.39	1268385	1684	1
**“***Candidatus* Rickettsia amblyommii ” GAT-30V [NC_017028, NC_017020, NC_017029, NC_017021]	232978	1	32.43	1407796	1846	3

**Taxon** [1]	**ID** [2]	**No** [3]**.**	**%GC**	**L (bases)**	**CDS** [4]	**Pl.** [5]
Rickettsia rhipicephali 3-7-female6-CWPP [NC_017042]	233851	1	32.41	1290368	1621	1
Rickettsia massiliae AZT80 [NC_016931, NC_016939]	238520	1	32.54	1263659	1601	1
Rickettsia massiliae MTU5 [NC_009900, NC_009897]	83254	1	32.61	1360898	1721	1
Rickettsia montanensis OSU 85-930 [NC_017043]	232555	1	32.57	1279798	1513	0
* "*Candidatus* Rickettsia gravesii" BWI-1 [NA]	302479	28	32.24	1327625	1687	0
Rickettsia japonica YH [NC_016050]	83739	1	32.35	1283087	1575	0
Rickettsia heilongjiangensis 054 [NC_015866]	193551	1	32.32	1278468	1562	0
* *Rickettsia honei* RB [NA]	229666	11	32.42	1268758	1614	0
Rickettsia peacockii Rustic [NC_012730, NC_012732]	48268	1	32.56	1288492	1558	1
Rickettsia philipii 364D [NC_016930]	124131	1	32.47	1287740	1570	0
Rickettsia rickettsii Hlp#2 [NC_016915]	236174	1	32.47	1270751	1574	0
Rickettsia rickettsii Colombia [NC_016908]	228121	1	32.46	1270083	1560	0
Rickettsia rickettsii Brazil [NC_016913]	239281	1	32.45	1255681	1547	0
Rickettsia rickettsii 'Sheila Smith' [NC_009882]	5337	1	32.47	1257710	1577	0
Rickettsia rickettsii Arizona [NC_016909]	236594	1	32.44	1267197	1580	0
Rickettsia rickettsii Hauke [NC_016911]	233768	1	32.45	1269721	1570	0
Rickettsia rickettsii Iowa [NC_010263]	59104	1	32.45	1268175	1595	0
Rickettsia rickettsii Hino [NC_016914]	235723	1	32.45	1269837	1593	**?**
Rickettsia slovaca D-CWPP [NC_017065]	233215	1	32.50	1275720	1598	0
Rickettsia slovaca 13-B [NC_016639]	180092	1	32.50	1275089	1611	0

Taxon [1]	ID [2]	No [3].	%GC	L (bases)	CDS [4]	Pl. [5]
Rickettsia parkeri Portsmouth [NC_017044]	233447	1	32.43	1300386	1604	0
Rickettsia africae ESF-5 [NC_012633, NC_012634]	6986	1	32.40	1278530	1545	1
Rickettsia sibirica 246 [NZ_AABW01000001]	27963	1	32.47	1250020	1554	0
Rickettsia sibirica subsp. *sibirica* BJ-90 [NA]	238733	8	32.45	1254734	1588	0
Rickettsia sibirica subsp. *mongolitimonae* HA-91 [NA]	225156	21	32.42	1252337	1616	0
Rickettsia conorii Malish 7 [NC_003103]	45613	1	32.44	1268755	1578	0
Rickettsia conorii subsp. *indica* ITTR [NA]	229600	10	32.44	1249482	1601	0
* *Rickettsia conorii* subsp *israelensis* ISTT CDC1 [NA]	230737	33	32.49	1252815	1640	0
* *Rickettsia conorii* subsp *caspia* A-167 [NA]	297	25	32.50	1260331	1657	0

[1] Taxa are listed from the **top to the bottom** as shown in the tree (**fig. 1*a***). Classification follows previous studies (Driscoll, et al. 2013; Gillespie, et al. 2007; Gillespie, et al. 2012). The term "*Candidatus*" is used as originally suggested (Murray and Stackebrandt 1995). *Incertae sedis*, "of uncertain placement", refers to taxa with unknown or undefined broader relationships. Asterisks denote genomes added since our last study (Driscoll, et al. 2013). Information pertaining to the genome sequences for *R. felis* strains LSU and LSU-Lb is provided in **Table 2**. Available GenBank accession numbers are listed under each taxon.

[2] PATRIC (Gillespie, et al. 2011; Wattam, et al. 2014) genome IDs. WGS project ID is provided for *Rickettsia australis* Phillips (this genome was not released on PATRIC as of 08/17/14.

[3] Total number of contigs.

[4] Total number of CDS predicted and annotated using RAST (Aziz, et al. 2008). For *R. felis* genomes, fgenesb (Tyson, et al. 2004) was also utilized to predict CDS: str. URRWXCal2 (1499), str. LSU (1859) and str. LSU-Lb (1576).

[5] Number of unique plasmids. We did not analyze plasmid sequences for *Rickettsia australis* Phillips, though this genome purportedly carries two plasmids (Dong, et al. 2012). The plasmid sequence released for *R. rickettsii* str. Hino was determined to be a contamination of likely Vero cell DNA.

Literature Cited

Aziz RK, et al. 2008. The RAST Server: rapid annotations using subsystems technology. BMC Genomics 9: 75. doi: 1471-2164-9-75 [pii]
10.1186/1471-2164-9-75

Dong X, El Karkouri K, Robert C, Raoult D, Fournier PE 2012. Genome sequence of *Rickettsia australis*, the agent of Queensland tick typhus. Journal of bacteriology 194: 5129. doi: 10.1128/JB.01117-12

Driscoll T, Gillespie JJ, Nordberg EK, Azad AF, Sobral BW 2013. Bacterial DNA sifted from the *Trichoplax adhaerens* (Animalia: Placozoa) genome project reveals a putative rickettsial endosymbiont. Genome Biol Evol 5: 621-645. doi: 10.1093/gbe/evt036
evt036 [pii]

Gillespie JJ, et al. 2007. Plasmids and rickettsial evolution: insight from *Rickettsia felis*. PLos One 2: e266. doi: 10.1371/journal.pone.0000266

Gillespie JJ, Nordberg EK, Azad AF, Sobral BW. 2012. Phylogeny and Comparative Genomics: The Shifting Landscape in the Genomics Era. In: Azad AF, Palmer GH, editors. Intracellular Pathogens II: Rickettsiales. Boston: American Society of Microbiology. p. 84-141.

Gillespie JJ, et al. 2011. PATRIC: the comprehensive bacterial bioinformatics resource with a focus on human pathogenic species. Infect Immun 79: 4286-4298. doi: 10.1128/IAI.00207-11
IAI.00207-11 [pii]

Murray RG, Stackebrandt E 1995. Taxonomic note: implementation of the provisional status Candidatus for incompletely described procaryotes. Int J Syst Bacteriol 45: 186-187.

Tyson GW, et al. 2004. Community structure and metabolism through reconstruction of microbial genomes from the environment. Nature 428: 37-43. doi: 10.1038/nature02340
nature02340 [pii]

Wattam AR, et al. 2014. PATRIC, the bacterial bioinformatics database and analysis resource. Nucleic Acids Res 42: D581-591. doi: 10.1093/nar/gkt1099
gkt1099 [pii]

Supplementary table S3. Twenty *Rickettsia* plasmids utilized for comparative analyses.

**Plasmid** [1]	**Taxon** [2]	**Classification** [3]	**ID** [4]
pREIS3	*Rickettsia* endosymbiont (*Ixodes scapularis*)	SFG	CM000773
pRM	*Rickettsia monacensis* IrR-Munich	SFG	NC_010927
pRAF	*Rickettsia africae* ESF-5	SFG	NC_012634
pREIS1	*Rickettsia* endosymbiont (*Ixodes scapularis*)	SFG	CM000771
pRPR	*Rickettsia peacockii* Rustic	SFG	NC_012732
pLbaR	*Rickettsia felis* LSU-Lb	TRG	(new here)
pREIS2	*Rickettsia* endosymbiont (*Ixodes scapularis*)	SFG	CM000772
pREIS4	*Rickettsia* endosymbiont (*Ixodes scapularis*)	SFG	CM000770
pRF	*Rickettsia felis* URRWXCal2	TRG	NC_007110
pRF	*Rickettsia felis* LSU	TRG	(new here)
pRF	*Rickettsia felis* "AUS-Lb"	TRG	GQ329881
pRF	*Rickettsia felis* LSU-Lb	TRG	(new here)
pMCE_3	"*Candidatus* Rickettsia amblyommii" GAT-30V	SFG	NC_017021
pMC5_1	*Rickettsia australis* Cutlack	TRG	NC_017041
pRhe	*Rickettsia helvetica* C9P9	*Incertae sedis*	AICO01000000
pMCE_1	"*Candidatus* Rickettsia amblyommii" GAT-30V	SFG	NC_017020
pMCE_2	"*Candidatus* Rickettsia amblyommii" GAT-30V	SFG	NC_017029
pMCC_1	*Rickettsia rhipicephali* 3-7-female6-CWPP	SFG	NC_017055
pRMB	*Rickettsia massiliae* AZT80	SFG	NC_016939
pRMA	*Rickettsia massiliae* MTU5	SFG	NC_009897

[1] Taxa are listed from the **top to the bottom** as shown in the tree (**fig. 2**). Asterisks denote genomes added since our last study (Driscoll, et al. 2013). Information pertaining to the genome sequences for *R. felis* strains LSU and LSU-Lb is provided in **Table 2**.

[2] The term "*Candidatus*" is used as originally suggested (Murray and Stackebrandt 1995).

[3] Classification follows previous studies (Driscoll, et al. 2013; Gillespie, et al. 2007; Gillespie, et al. 2012). *Incertae sedis*, "of uncertain placement", refers to taxa with unknown or undefined broader relationships.

[4] NCBI accession numbers.

Literature Cited

Driscoll T, Gillespie JJ, Nordberg EK, Azad AF, Sobral BW 2013. Bacterial DNA sifted from the *Trichoplax adhaerens* (Animalia: Placozoa) genome project reveals a putative rickettsial endosymbiont. Genome Biol Evol 5: 621-645. doi: 10.1093/gbe/evt036
evt036 [pii]

Gillespie JJ, et al. 2007. Plasmids and rickettsial evolution: insight from *Rickettsia felis*. PLos One 2: e266. doi: 10.1371/journal.pone.0000266

Gillespie JJ, Nordberg EK, Azad AF, Sobral BW. 2012. Phylogeny and Comparative Genomics: The Shifting Landscape in the Genomics Era. In: Azad AF, Palmer GH, editors. Intracellular Pathogens II: Rickettsiales. Boston: American Society of Microbiology. p. 84-141.

Murray RG, Stackebrandt E 1995. Taxonomic note: implementation of the provisional status Candidatus for incompletely described procaryotes. Int J Syst Bacteriol 45: 186-187.
