# Supplementary material for: Genomic Diversification in Strains of Rickettsia felis Isolated from Different Arthropods
Source: Genome Biol Evol. 2014 Dec 4;7(1):35–56. doi: 10.1093/gbe/evu262 (PMC4316617; doi:10.1093/gbe/evu262)
Supplement: Supplementary Data [file supp_evu262_New_Microsoft_Office_Word_Document.docx]

**Supplementary Material**

**Supplementary table S1.** Primers used to confirm predicted SNPs.

**Supplementary table S2.** Rickettsiaceae genomes utilized for phylogenomics analysis.

**Supplementary table S3.** Twenty *Rickettsia* plasmids utilized for comparative analyses.

**Supplementary table S4.** Description of estimated polymorphism across *Rickettsia* *felis* strains.

**Supplementary fig. S1**. Characteristics of the *Wolbachia* 16S rRNA gene concurrently sequence and assembled with *R*. *felis* str. LSU.

**Supplementary fig. S2.** Generation of orthologous groups (OGs) of proteins across three *R*. *felis* genomes.

**Supplementary fig. S3.** Synteny analysis across three *R*. *felis* genomes (chromosomes).

**Supplementary fig. S4.** Comparison of 16S rDNA, *gltA*, *sca0*, *sca4*, and *sca5* sequences across selected *R*. *felis* strains.

**Supplementary fig. S5.** Synteny analysis of four *R*. *felis* pRF plasmids.

**Supplementary fig. S6**. Comparative analysis of diverse pRF plasmids.

**Supplementary fig. S7.** *R*. *felis* str. LSU-Lb carries a novel rickettsial plasmid, pLbaR.

**Supplementary fig. S8.** Phylogeny estimation of DUF1016 proteins.

**Supplementary fig. S9.** Plasmid pLbaR of *R*. *felis* str. LSU-Lb encodes an RHS-like toxin adjacent to an RTX-like T1SS operon.
